# Four New Compounds Obtained from Cultured Cells of *Artemisia annua*

**DOI:** 10.3390/molecules22122264

**Published:** 2017-12-18

**Authors:** Jianhua Zhu, Peijie Xiao, Minghua Qian, Chang Chen, Chuxin Liang, Jiachen Zi, Rongmin Yu

**Affiliations:** 1 Biotechnological Institute of Chinese Materia Medical, College of Pharmacy, Jinan University, Guangzhou 510632, China; peijiexiao@163.com (P.X.); tzjc@jnu.edu.cn (J.Z.); 2 Department of Natural Products Chemistry, College of Pharmacy, Jinan University, Guangzhou 510632, China; qianmhlw@163.com (M.Q.); imchenchang@163.com (C.C.); lcxliangcx@163.com (C.L.)

**Keywords:** *Artemisia annua* cultured cells, arteannuin I, arteannuin J, biosynthesis, selective hydroxylation

## Abstract

Four new compounds obtained from cultured cells of *Artemisia annua* were reported. Products were detected by HPLC-ELSD/GC-MS and isolated by chromatographic methods. The structures of four new compounds, namely 6-hydroxy arteannuin I (**1**), 1-hydroxy arteannuin I (**2**), 2-hydroxy arteannuin J (**3**), and 14-hydroxy arteannuin J (**4**), were elucidated using their physico-chemical properties by NMR and MS data analyses. The results from the spontaneous oxidative experiment indicated that the biosynthesis of the new compounds was enzyme-catalyzed. Interestingly, the enzymes in the cultured cells of *A. annua* showed the abilities of substrate-selective and region-selective hydroxylation of the sesquiterpene lactone. Furthermore, the artemisinin contents were increased by 50% and 80% compared to the control group after the addition of arteannuin I/J to the suspension-cultured cells of *A. annua* under light and dark culture conditions, respectively.

## 1. Introduction

Malaria is one of the most prevalent and devastating parasitic diseases worldwide, occurring mostly in tropical and subtropical Africa, Asia and South America. In 2015, approximately 212 million malaria-related diseases were reported, and nearly 429,000 people were killed by the disease. Most of these deaths occurred in South African children under the age of five [1]. The World Health Organization recommended the use of artemisinin-based combination therapies (ACTs) for the treatment of uncomplicated malaria caused by the parasite *Plasmodium falciparum* [2]. 

Artemisinin is a sesquiterpene endoperoxide isolated from *Artemisia annua* with potent antimalarial properties. The relatively low yield (0.01–0.8%) of artemisinin in *A. annua* is a serious limitation to its commercialization [3]. In 2012, the Cook group published an approach to the synthesis of artemisinin [4]. The synthesis proceeded in five steps from cyclohexanone. Although the total chemical synthesis of artemisinin has been a success, its complex steps, by-products, low yield (3.87–13.31%), environmental damage, and high cost of production highly restrict its industrial application [5]. Attempts to produce artemisinic acid (one of the proposed precursors of artemisinin) in developed strains of *Saccharomyces cerevisiae* have been successful, and chemical processes for the conversion of artemisinic acid to artemisinin have also been established [6,7]. However, artemisinin is not directly produced in any engineered yeast. Therefore, considerable interest in recent years has focused on understanding the natural biosynthetic pathways of artemisinin [8].

It is well known that the biosynthesis of artemisinin includes the following three stages: the formation of farnesyl-pyrophosphate (FPP) from acetyl-CoA, the synthesis of sesquiterpene, and the formation of artemisinin via lactonization and peroxidation. The mechanism of enzyme catalysis in the former two stages has been studied, but the mechanism of the last step, involving the conversion of dihydroartemisinic acid to artemisinin, remains unclear [9,10,11].

Precursor feeding is one of the most effective strategies employed to increase the production of artemisinin in cells and organ cultures [12]. Further research could be performed to determine more direct precursors associated with artemisinin biosynthesis. Arteannuin I/J are parts of an isomer pair derived from the secondary metabolites of *A. annua*, with a content of only 0.011% [13]. Arteannuin I/J are the metabolites of dihydroartemisinic acid isolated from the feeding experiment of [15-^13^C_2_H_3_]-dihydroartemisinic acid to *A. annua* [14]. The production of arteannuin I is approximately 2-fold higher than that of dihydroarteannuin B, and 10-fold higher than that of arteannuin K and arteannuin L. In addition, it has the same conversion rate as deoxyartemisinin. We previously reported that arteannuin I was a lactone metabolite of dihydroartemisinic acid, and might play an important role in artemisinin biosynthesis [15]. Thus, arteannuin I/J could be used as intermediates in the biosynthetic pathway of dihydroartemisinic acid to artemisinin in *A. annua*. The present paper describes the biotransformation of arteannuin I/J in suspension-cultured cells of *A. annua*. Four new compounds (**1**–**4**) were isolated, and their structures were found to be 6-hydroxy arteannuin I (**1**), 1-hydroxy arteannuin I (**2**), 2-hydroxy arteannuin J (**3**), and 14-hydroxy arteannuin J (**4**) on the basis of their physicochemical properties and spectral data analyses. 

## 2. Results and Discussion

### 2.1. Detection of the New Products by HPLC-ELSD and GC-MS

**Figure 1:**
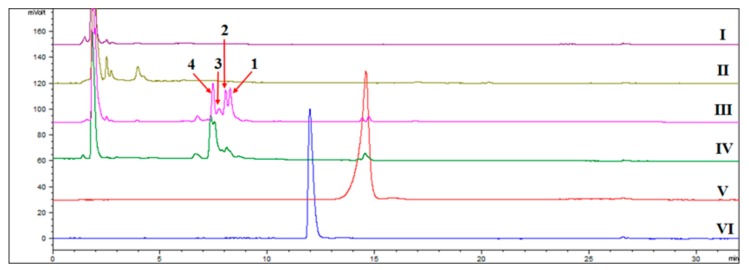
HPLC-ELSD detection of new products in suspension-cultured cells of *A. annua*. (**I**): The extract of the control group cultured under dark conditions; (**II**): The extract of the control group cultured under light conditions; (**III**): The extract of suspension-cultured cells in the presence of arteannuin I/J cultured under dark conditions; (**IV**): The extract of cultured cells in the presence of arteannuin I/J cultured under light conditions; (**V**): arteannuin I/J; (**VI**): artemisinin; **1**: 6-hydroxy arteannuin I; **2**: 1-hydroxy arteannuin I; **3**: 2-hydroxy arteannuin J; **4**: 14-hydroxy arteannuin J.

**Figure 2:**
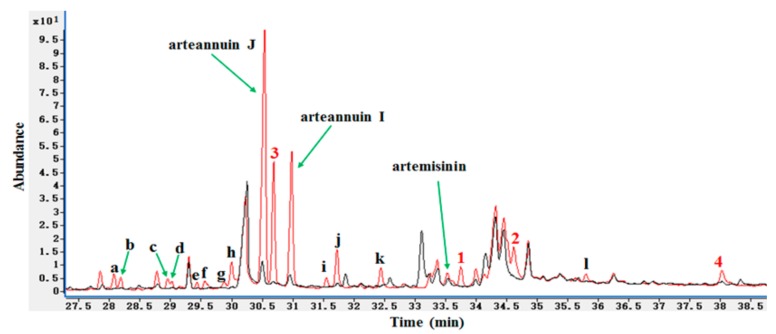
GC-MS detection of new products in cultured cells of A. annua. Black curve: The medium extract of *A. annua* cultured cells without the treatment of arteannuin I/J; Red curve: The medium extract of *A. annua* cultured cells with the treatment of arteannuin I/J; **a**–**l**: 12 unknown new peaks.

HPLC-ELSD revealed several significant new peaks at 6–9.5 min in the biotransformation products of arteannuin I/J, including the isolated products **1**–**4** (Figure 1). At least 16 new peaks were identified by GC-MS after the addition of arteannuin I/J to the suspension-cultured cells of *A. annua*, suggesting that enzyme-catalyzed or autoxidation reactions occurred (Figure 2). As shown in GC-MS, the same products were formed in cultured cells under light and dark conditions (Supplementary Materials). Among them, four peaks were structurally determined to be compounds **1**–**4**. 

### 2.2. Effects of Arteannuin I/J Dosage on Cell Growth and the Content of New Products

**Figure 3:**
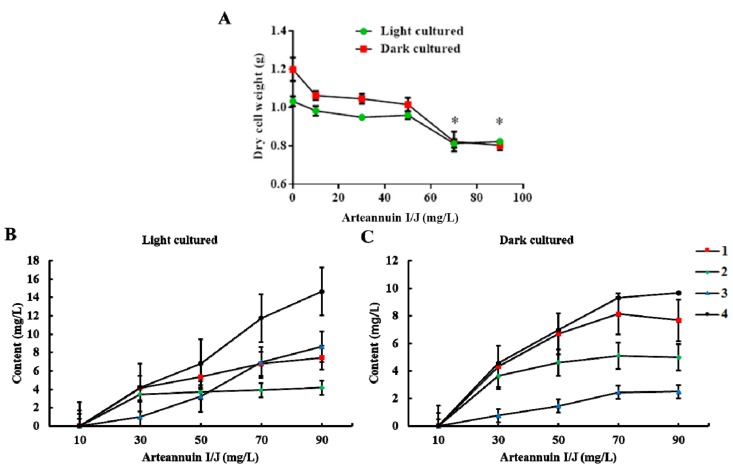
Effects of arteannuin I/J dosage on cell growth and the content of new products. All values present the mean of three cultures replicated three times (* *p* < 0.05 compared with the control group by Tukey’s test). (**A**) Effects of arteannuin I/J dosage on cell growth under light and dark cultured conditions; (**B**) Effects of arteannuin I/J dosage on the content of 1–4 under light cultured condition; (**C**) Effects of arteannuin I/J dosage on the content of 1–4 under dark cultured condition.

The dry cell weight of *A. annua* decreased with the increasing dose of arteannuin I/J (Figure 3A). When the concentration of arteannuin I/J reached 70 mg/L, the growth of suspension-cultured cells of *A. annua* was inhibited. A concentration of 50 mg/L of arteannuin I/J led to a maximal conversion efficiency of **1**–**4** (44.9%, 24.8%, 10.8% and 22.9% under light conditions, and 45.9%, 31.6%, 5.0% and 23.9% under dark conditions, respectively) (Figure 3B,C and Supplementary Materials). The difference in the conversion of **2** and **3** between light and dark conditions might be related to the varying levels of enzyme expression in *A. annua* cells. The artemisinin content increased from 83.0 μg/L and 62.1 μg/L to 125.1 μg/L and 116.8 μg/L, respectively, after the addition of 50 mg/L arteannuin I/J to the two culture systems. These results indicated that arteannuin I/J play key roles as precursors in artemisinin biosynthesis. 

### 2.3. Time Course of Biosynthesis Reaction

**Figure 4:**
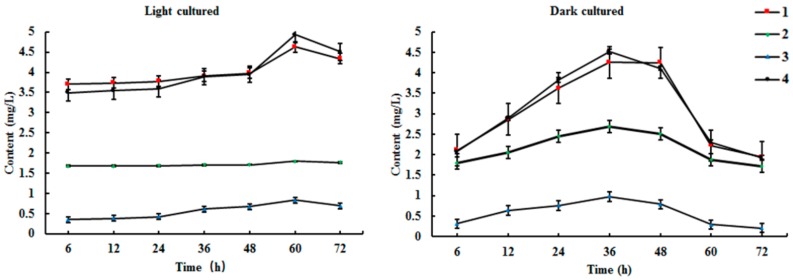
Time course of biotransformation of arteannuin I/J in cultured cells of *A. annua* (*n* = 3).

Results from HPLC-ELSD showed that the new products were mostly present in the ethyl acetate extract of the medium. However, artemisinin was mostly found in the cell extracts. Biosynthesis reaction occurred within 6 h in the cell culture system under light conditions, and the product contents reached a maximum at 60 h (Figure 4 and Supplementary Materials). Similarly, the content of products increased significantly, and reached a maximum at 36 h in cultured cells under dark conditions. Therefore, the optimal times for the conversion of arteannuin I/J under light and dark conditions were 60 h and 36 h, respectively.

The new products did not appear in the control experiments using a cell-free medium, suggesting that the reaction was enzyme-catalyzed. Since no product was found in the ethyl acetate extract of cells, it was supposed that products were synthesized on the cell membrane. Otherwise, the different content of biosynthesis products indicated that the enzymes in *A. annua* cells possess the abilities of substrate-selective and region-selective hydroxylation of arteannuin I/J.

### 2.4. Structural Elucidation of the New Products

The results of High-resolution mass spectra (HRESIMS) analysis for compound **1** showed a molecular formula of C_15_H_22_O_3_ (*m*/*z* 273.1444 for [M + Na]^+^). The structure of **1** was deduced from the interpretation of 1D and 2D NMR experiments (Table 1 and Supplementary Materials). The presence of functional groups such as the terminal double bond (δ_C_ 145.35 (C-4), δ_C_ 105.77 (C-15); δ_H_ 5.08 (s, H-15), δ_H_ 4.85 (d, *J* = 1.7 Hz, H-15)) and the endoperoxide lactone (δ_C_ 172.79 (C-12), δ_C_ 76.79 (C-5); δ_H_ 4.76 (d, *J* = 12.1 Hz, H-5)) were found in the 1-D NMR spectra (^1^H, ^13^C/DEPT) [13]. ^1^H and ^13^C-NMR spectra as well as the Heteronuclear Singular Quantum Correlation (HSQC) spectrum demonstrated the presence of two methyl groups (δ_C_ 8.90, δ_H_ 1.29 (d, *J* = 7.2 Hz, 3H); δ_C_ 19.90, δ_H_ 0.94 (d, *J* = 6.2 Hz, 3H)), four methylenes (δ_C_ 28.22, δ_H_ 2.02 (m, 1H)/1.52 (d, *J* = 3.8 Hz, 1H); δ_C_ 28.44, δ_H_ 1.42 (m, 1H)/1.69(m, 1H); δ_C_ 29.85, δ_H_ 2.29 (m, 1H)/1.24 (m, 1H); δ_C_ 30.15, δ_H_ 2.20 (m, 1H)/1.73 (dt, *J* = 5.6, 2.8 Hz, 1H)), four methines (δ_C_ 27.75, δ_H_ 1.76 (m, 1H); δ_C_ 38.64, δ_H_ 2.00 (m, 1H); δ_C_ 48.73, δ_H_ 2.57 (d, *J* = 7.1 Hz, 1H); δ_C_ 51.20, δ_H_ 1.82 (d, *J* = 4.2 Hz, 1H)), and one quaternary carbon (δ_C_ 71.71). The δ_H_ 1.97 (m, H-6) of arteannuin I upshifted to δ_H_ 1.58 (s, 1H) in the ^1^H-NMR spectrum of **1**. The ^13^C-NMR spectrum showed that a methine (δ_C_ 45.50, C-6) changed to an sp^3^-hybridized quaternary carbon (δ_C_ 71.71). Based on the ^1^H-NMR and ^13^C-NMR spectra, a hydroxyl group had been substituted at C-6 of artannuin **I** in the structure of **1**. Therefore, we could infer the structure of **1** by the relevant carbon and hydrogen chemical shift values at the changed site.

**Figure 5:**
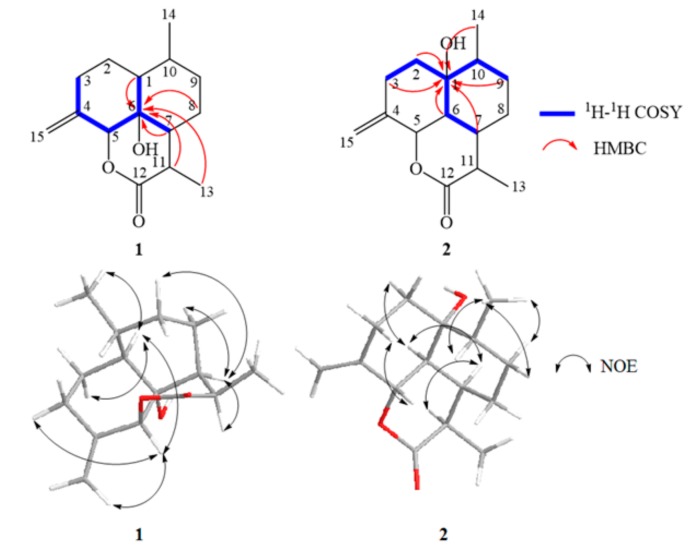
Key COSY, HMBC and NOE correlations of compounds **1** and **2**.

The COSY correlations between δ_H_ 4.76/δ_H_ 1.76, δ_H_ 4.76/δ_H_ 1.82, and δ_H_ 4.76/δ_H_ 2.29 and protons H-1, H-7, H-8, H-11, and H-13 to δ_C_ 71.71 observed in HMBC suggested a carbon atom connection of the ternary ring of **1** (Figure 5). The NOE correlations between H-5/H-1, H-5/H-3 (δ_H_ 2.29), H-5/H-15 (δ_H_ 5.08), H-1/H-2 (δ_H_ 1.42), H-1/Me-14, H-7/H-11, H-7/H-8 (δ_H_ 2.02), and H-7/H-9 (δ_H_ 2.20) indicated the α-orientation of Me-14, H-1, H-5, H-7, and H-11, but the β-orientation of H-10. Furthermore, the relative configuration of OH-6 was α-oriented. Based on the results, the structure of **1** was determined to be (3*R*,3*aS*,3*a*^1^*R*,6*R*,6*aS*,9*aS*)-3a1-hydroxy-3,6-dimethyl-9-methylenedeca-hydrobenzo [*de*] chromen-2 (*3H*)-one (Figure 7). 

The molecular formula of compound **2** determined by HRESIMS data was C_15_H_22_O_3_, indicating that it is an isomer of **1** with five degrees of unsaturation. Functional groups such as the terminal double bond (δ_C_ 144.39 (C-4), δ_C_ 106.69 (C-15); δ_H_ 5.13 (d, *J* = 1.2 Hz, H-15), δ_H_ 4.88 (d, *J* = 1.5 Hz, H-15)) and the endoperoxide lactone (δ_C_ 174.33 (C-12), δ_C_ 76.17 (C-5); δ_H_ 4.91 (d, *J* = 12.3 Hz, H-5)) were proposed for the structure of **2** based on the 1-D NMR spectra (^1^H, ^13^C/DEPT) (Table 1 and Supplementary Materials) [13].

^1^H- and ^13^C-NMR spectra as well as the HSQC data indicated the presence of two methyl groups (δ_C_ 13.54, δ_H_ 1.24 (d, *J* = 7.2 Hz, 3H); δ_C_ 14.36, δ_H_ 0.92 (d, *J* = 6.6 Hz, 3H)), four methylenes (δ_C_ 22.51, δ_H_ 1.78 (m, 1 H)/1.30 (m, 1H); δ_C_ 29.57, δH 2.34 (m, 1H)/2.06 (m, 1H); δ_C_ 29.82, δ_H_ 1.46 (dd, *J* = 7.6, 5.1 Hz, 1H)/1.68 (dt, *J* = 12.9, 3.4 Hz, 1H); δ_C_ 37.74, δ_H_ 1.40 (m, 1H)/2.16 (m, 1H)), four methane groups (δ_C_ 31.56, δ_H_ 1.93 (d, *J* = 6.5 Hz, 1H); δ_C_ 34.74, δ_H_ 2.39 (dd, *J* = 9.4, 3.6 Hz, 1H); δ_C_ 40.23, δ_H_ 2.64 (p, *J* = 7.2 Hz, 1H); δ_C_ 51.29, δ_H_ 1.87 (dd, *J* = 11.8, 3.9 Hz, 1H)), and one quaternary carbon (δ_C_ 73.36). The δ_H_ 1.76 (m, H-1) of arteannuin I upshifted to δ_H_ 1.60 (s, 1H) in the ^1^H-NMR spectrum of **2**. The ^13^C-NMR spectrum showed that a methine (δ_C_ 43.70, C-1) changed to a sp^3^-hybridized quaternary carbon (δ_C_ 73.36). Based on the ^1^H-NMR and ^13^C-NMR spectra, a hydroxyl group is substituted at the C-1 of arteannuin I in the structure of **2**.

The COSY correlations between δ_H_ 1.68/δ_H_ 1.93, δ_H_ 1.87/δ_H_ 1.40, δ_H_ 1.87/δ_H_ 2.34, δ_H_ 1.87/δ_H_ 2.39, and δ_H_ 1.87/δ_H_ 1.46 and protons H-2, H-3, H-6, H-7, H-9, and H-14 correlated to δ_C_ 73.36 observed in HBMC suggested a carbon atom connection of the ternary ring of 2 (Figure 5). The NOE correlations between Me-14/H-9 (δ_H_ 1.40), Me-14/H-10, H-2 (δ_H_ 1.40)/H-6, H-6/H-7, H-7/H-11 and H-3 (δ_H_ 2.06)/H-5 indicated the α-orientation of Me-14, H-6, H-7, and H-11, but the β-orientation of H-5 and H-10. Furthermore, the relative configuration of OH-1 was α-oriented. Based on the results, the structure of **2** was determined to be (3*R*,3*aR*,3*a*^1^*S*,6*R*,6*aR*,9*aS*)-6a-hydroxy-3,6-dimethyl-9 methylene-decahydrobenzo [*de*] chromen-2 (*3H*)-one (Figure 7). 

The molecular formula of compound **3** determined by HRESIMS data was C_15_H_22_O_3_ with five unsaturated degrees. The NMR spectra (^1^H, ^13^C/DEPT) detected functional groups such as the double bond (δ_C_ 145.35 (C-4), δ_C_ 124.16 (C-3); δ_H_ 5.57 (m, 1H) and the endoperoxide lactone (δ_C_ 175.14 (C-12), δ_C_ 74.92 (C-5); δ_H_ 4.84 (d, *J* = 11.1 Hz, H-5)) (Table 2 and Supplementary Materials) [13].

^1^H- and ^13^C-NMR spectra as well as the HSQC spectrum showed the presence of three methyl groups (δ_C_ 13.45, δ_H_ 1.25 (d, *J* = 7.3 Hz, 3H); δ_C_ 18.44, δ_H_ 1.86 (overlap, 3H), δ_C_ 20.17, δ_H_ 0.98 (d, *J* = 6.2 Hz, 3H)), two methylenes (δ_C_ 23.11, δ_H_ 1.76 (m, 1H)/1.33 (dd, *J* = 9.9, 4.1 Hz, 1H); δ_C_ 34.96, δ_H_ 1.07 (dd, *J* = 12.0, 4.2 Hz, 1H)/1.86 (overlap, 1H)), and six methines (δ_C_ 28.09, δ_H_ 1.29 (m, 1H); δ_C_ 35.88, δH 2.38 (dt, *J* = 11.0, 3.6 Hz, 1H); δ_C_ 38.64, δ_H_ 1.94 (m, 1H); δ_C_ 40.87, δ_H_ 2.78 (p, *J* = 7.2 Hz, 1H); δ_C_ 50.27, δ_H_ 1.58 (d, *J* = 4.0 Hz, 1H); and δ_C_ 65.48, δ_H_ 4.18 (d, *J* = 11.1 Hz, 1H)). The δ_H_ 2.15 and δ_H_ 2.25 of arteannuin J shifted to δ_H_ 1.60 (s, 1H) and δ_H_ 4.18 (d, *J* = 4.1 Hz, 1H) in the ^1^H-NMR spectrum of **3**. The ^13^C-NMR spectrum demonstrated that a methylene (δ_C_ 27.20, C-2) changed to an oxygen methine (δ_C_ 65.48). Based on the ^1^H-NMR and ^13^C-NMR spectra, a hydroxyl group is substituted at C-2 of arteannuin J in the structure of **3**.

**Figure 6:**
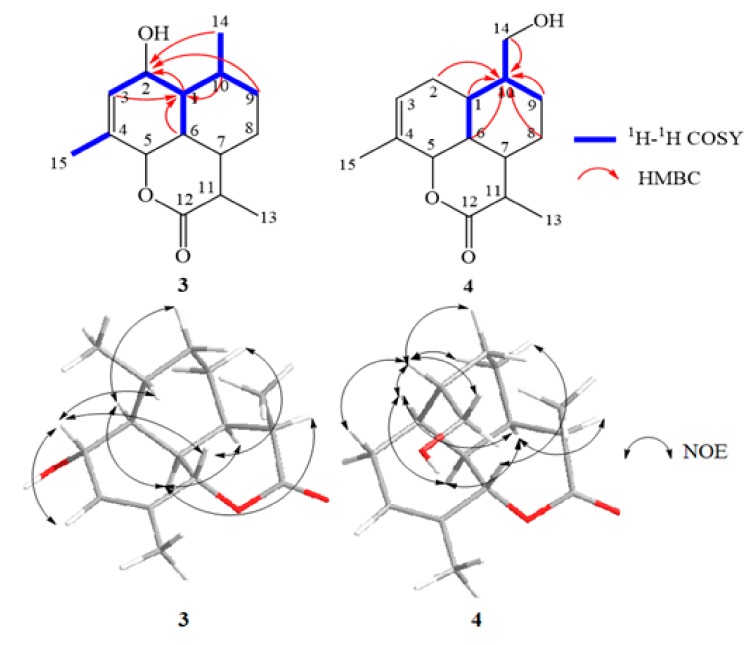
Key COSY, HMBC and NOE correlations of compounds **3** and **4**.

**Figure 7:**
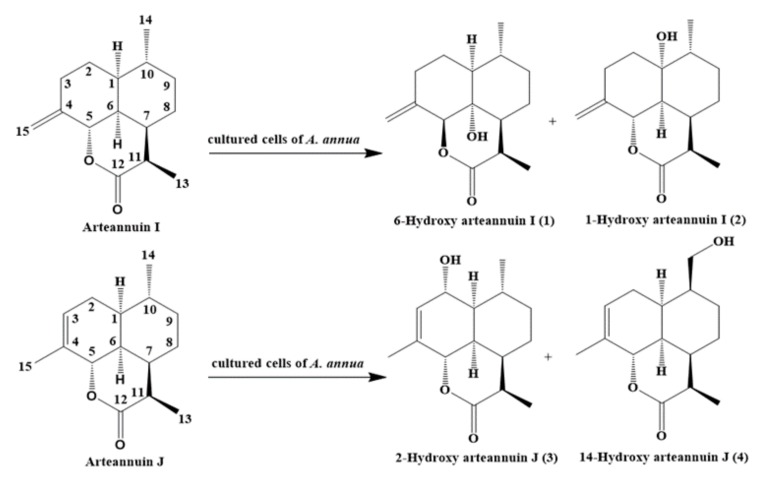
Proposed biotransformation scheme of arteannuin I/J in cultured cells of *A. annua*.

The correlations between H-2 (δ_H_ 4.18)/H-1, H-2/H-3, H-2/Me-15, H-1/H-6, H-1/H-9 (δ_H_ 1.07), H-1/Me-14, and H-9 (δ_H_ 1.86)/H-10 in the ^1^H-^1^H COSY spectrum and the HMBC correlation from H-1, H-9, and Me-14 to δ_C_ 65.48 as well as H-3, H-6, and H-10 to δ_C_ 50.27 (C-1) proposed the structure of **3** in Figure 6. The NOE correlations between H-2/H-3, H-2/H-5, H-2/H-10, H-5/H-8 (δ_H_ 1.33), H-1/H-6, H-1/H-9 (δ_H_ 1.07), H-6/H-7, and H-6/H-11 indicated the β-orientation of H-2, H-5 and H-10 but the α-orientation of OH-2, H-1, H-6, H-7 and H-11. Based on the results, the structure of **3** was shown to be (3*R*,3*aR*,3*a*^1^*R*,6*R*,6*aS*,7*R*,9*aS*)-7-hydroxy-3,6,9-trimethyl-3*a*,3*a*^1^,4,5,6,6*a*,7,9*a*-octahydrobenzo [*de*] chromen-2 (*3H*)-one (Figure 7). 

Compound **4** was isolated as yellow oil. Its molecular formula, C_15_H_22_O_3_, was evident from the HRESIMS data. The 1-D NMR spectra (^1^H, ^13^C/DEPT) detected functional groups such as the double bond (δ_C_ 132.08 (C-4), δ_C_ 122.77 (C-3); δ_H_ 5.39 (m, H-3)) and the endoperoxide lactone (δ_C_ 175.43 (C-12), δ_C_ 75.28 (C-5); δ_H_ 4.95 (d, *J* = 10.8 Hz, H-5)) in **4** (Table 2 and Supplementary Materials) [13].

^1^H- and ^13^C-NMR spectra as well as the HSQC experiment showed the presence of two methyl groups (δ_C_ 13.48, δ_H_ 1.25 (d, *J* = 7.3 Hz, 3H); δ_C_ 18.43, δ_H_ 1.79 (dd, *J* = 2.4, 1.2 Hz, 3H)), four methylenes (δ_C_ 22.76, δ_H_ 1.83 (m, 1H)/1.39 (dd, *J* = 13.2, 3.8 Hz, 1H); δ_C_ 27.12, δ_H_ 2.25 (overlap, 2H); δ_C_ 29.31, δ_H_ 1.25 (d, *J* = 7.3 Hz, 1H)/2.01(m, 1H)); δ_C_ 65.67, δ_H_ 3.69 (dd, *J* = 10.8, 3.3 Hz, 1H)/3.53 (dd, *J* = 10.8, 5.8 Hz, 1H)), and five methines (δ_C_ 36.23, δ_H_ 1.87 (m, 1H); δ_C_ 36.23, δ_H_ 1.52 (m, 1H); δ_C_ 38.73, δ_H_ 1.96 (m, 1H); δ_C_ 40.40, δ_H_ 2.19 (dt, *J* = 10.8, 3.7 Hz, 1H); δ_C_ 40.80, δ_H_ 2.77 (p, *J* = 7.2 Hz, 1H)) in **4**. δ_H_ 0.88 (m, H-6) of arteannuin J shifted to δ_H_ 3.69 and δ_H_ 3.53, and an overlapping δ_H_ 2.25 was observed in the ^1^H-NMR spectrum. The ^13^C-NMR spectrum indicated that a methyl (δ_C_ 20.20, C-14) changed to an oxygen methylene (δ_C_ 65.67). Based on the ^1^H-NMR and ^13^C-NMR spectra, a hydroxyl group is substituted at C-14 of arteannuin J in the structure of **4**.

The COSY correlations between H-14/H-10, H-10/H-1, H-10/H-6, and H-10/H-9 (δ_H_ 1.25) and protons H-1, H-2, H-6, H-8, H-9, H-10, and H-14 correlated to δ_C_ 65.67 observed in the HMBC spectrum indicated a connection between the following carbons: C-1/C-2/C-6/C-8/C-9/C-10/C-14 (Figure 6). The NOE correlations between H-10/H-1, H-10/H-2, H-10/H-8 (δ_H_ 1.83), H-10/H-9 (δ_H_ 1.25), H-10/H-14, H-1/H-6, H-1/H-7, H-6/H-7, H-7/H-11, and H-5/H-8 (δ_H_ 1.39) demonstrated an α-orientation of H-1, H-6, H-7, H-10, and H-11 but the β-orientation of H-5. Therefore, compound **4** was proposed to be (3*R*,3*aR*,3*a*^1^*R*,6*R*,6*aS*,7*R*,9*aS*)-6-(hydroxymethyl)-3,9-dimethyl-3*a*,3*a*^1^,4,5,6,6*a*,7,9*a*-octahydrobenzo [*de*] chromen-2 (*3H*)-one (Figure 7).

### 2.5. Detection of Spontaneous Autoxidation Products of Arteannuin I/J

The natural product dihydroartemisinic acid undergoes spontaneous autoxidation either as a solid or in organic solution, yielding artemisinin [16]. It is interesting to note that arteannuin I/J underwent spontaneous autoxidation during storage. The reaction process was slow and appeared to proceed according to the theory for singlet oxygen. 

**Figure 8:**
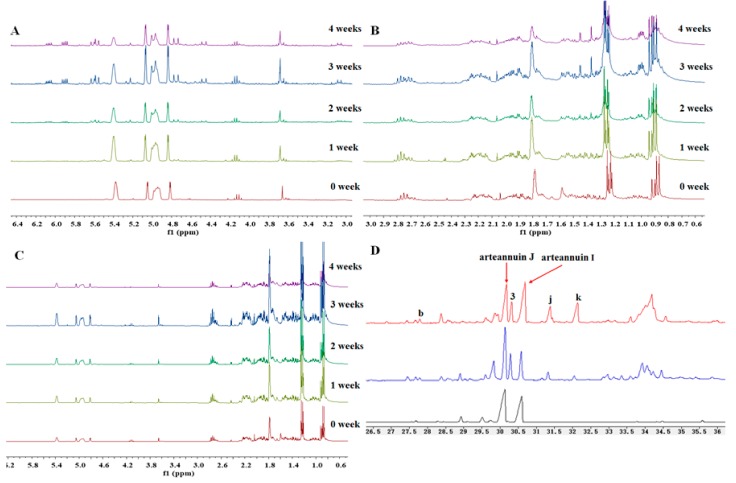
Detection of spontaneous autoxidation products of arteannuin I/J by NMR and GC-MS analyses. (**A**,**B**): ^1^H-NMR spectra of spontaneous autoxidation products under light conditions in CDCl_3_; (**C**): ^1^H-NMR spectra of spontaneous autoxidation products under dark conditions in CDCl_3_; (**D**): Arteannuin I/J (black), cultured cells in the presence of arteannuin I/J under light conditions (blue), spontaneous autoxidation products of arteannuin I/J (red); **b**, **j**, **k**: Three unknown new peaks; **3**: 2-hydroxy arteannuin J.

The CDCl_3_ solution of arteannuin I/J was maintained in an NMR tube under laboratory conditions for several weeks, and ^1^H-NMR spectra were recorded. Several new peaks appeared within a few weeks and after four weeks, there were obvious changes in arteannuin I/J in the ^1^H-NMR spectra (Figure 8A,B). Although these spectra were complex, some peaks were identified by comparison with the control group. In particular, several resonances appeared at δ_H_ 6.07 (dd, *J* = 9.9, 5.1 Hz), δ_H_ 5.90 (dd, *J* = 10.1, 5.0 Hz), δ_H_ 5.59 (dd, *J* = 13.7, 9.9 Hz), δ_H_ 4.75 (d, *J* = 12.9 Hz), δ_H_ 4.47 (d, *J* = 12.5 Hz), δ_H_ 4.14 (d, *J* = 7.1 Hz), δ_H_ 1.45 (s), δ_H_ 1.33 (s), and δ_H_ 1.00 (dd, *J* = 6.5, 3.2 Hz), with δ_H_ 5.37, δ_H_ 4.93, δ_H_ 2.25, and δ_H_ 2.15 showing obvious weakening. Among these novel peaks, the resonance at δ_H_ 1.00 ppm for the H-14 methyl group was clearly observed and followed the same pattern of change in intensity with time. No products were observed when the NMR solutions of arteannuin I/J were maintained in the dark (Figure 8C), which indicated that the initial conversion of arteannuin J into **3** requires light, and therefore almost certainly involves singlet oxygen (^1^O_2_). 

GC-MS was used to confirm the intermediacy of **3** in the spontaneous autoxidation of arteannuin J (Figure 8D). Furthermore, the other isolated products (**1**, **2** and **4**) were not detected in the GC-MS experiments. Compound **3** was detected by HPLC-ELSD after incubation of arteannuin J with the cultured cells of *A. annua* for 6 h. However, the spontaneous autoxidation experiment involving arteannuin J revealed that it took at least 2 weeks to be oxidized to **3** under natural conditions. These results suggested that it was catalyzed by an enzyme, or at least there was an environment conducive to the oxidation of arteannuin J in the cells of *A. annua*. Quite remarkably, the yield of **3** in cultured cells under light conditions was 2-fold higher than that of cells in the dark when feeding arteannuin I/J to *A. annua* under optimal culture conditions. This further demonstrated that the conversion of arteannuin J into **3** in cultured cells of *A. annua* was catalyzed by an enzyme, and that light promoted its activity. Cytochrome P_450s_ (CYPs) catalyze a wide variety of oxygenation/hydroxylation reactions that facilitate diverse metabolic functions in plants. Such CYPs have been shown to be involved in hydroxylation reactions resulting in inactivation of the actions of terpenoids [17,18,19]. Therefore, we hypothesized that the conversion of arteannuin I/J into **1**–**4** in cultured cells of *A. annua* was catalyzed by CYPs.

## 3. Materials and Methods

### 3.1. General

The results of high-resolution mass spectra (HRESIMS) analysis for compound **1** showed a molecular formula of C_15_H_22_O_3_. NMR spectra were recorded on a Bruker Avance III 300/500 apparatus using CDCl_3_ as solvents. High-resolution mass spectra were recorded on an Agilent 6210 LC/MSD TOF spectrometer. Silica gel (100–200 and 200–300 mesh) was used for column chromatography (CC), and silica GF 254 (10–40 μ) for Thin-Layer Chromatography (TLC) was supplied by the Qingdao Marine Chemical Factory, China. The HPLC/ELSD was run on an HPLC 1200-Agilent coupled to Alltech ELSD 2000ES using Agilent Hypersil ODS column (φ 4.6 × 250 mm, 5 μm) and guard column (4.6 mm × 12.5 mm, 5 μm). A binary gradient elution system consisted of water (A) and acetonitrile (B) and a separation procedure was achieved using the following gradient program: 0–20 min 30–100% B, 20–23 min 100% B, 23–25 min 100–30% B, 25–32 min 30% B. The flow rate was 0.8 mL/min, and the system was operated at 30 °C. The HPLC-ELSD detection conditions were as follows: drift tube temperature: 45 °C; carrier gas flow rate: 2.0 L/min; Impactor: On; amplification factor: 2. Detection wavelength was set at 230 nm. GC-MS was performed in a gas chromatographer GC 7890B-5977A MSD (Agilent, Santa Clara, CA, USA). The GC was set at the following conditions: the column: HP-5ms Ultra Inert (30 m × 0.25 mm, 0.25 μm); the carrier gas: helium; pressure: 10.795 psi; average linear velocity: 39.135 cm/s; column flow: 1.1208 mL/min; purge flow: 3.0 mL/min, and not shunt. Column temperature was set at 80 °C for 1 min, then 4 °C/min to 206 °C, 3 °C /min to 230 °C, and finally 15 °C /min to 300 °C for 20 min. The inlet heater was set at 280 °C, solvent delay time was 2 min, and mass scan parameter was from 35 to 500. 

### 3.2. Chemical Synthesis of Arteannuin I/J from Dihydroartemisinic Acid

The synthesis of dihydroartemisilacton was described by Zhang et al. [20]. Dihydroartemisilacton was synthesized from dihydroartemisinic acid. A solution of (COCl)_2_ (63.6 mmol) in CH_2_Cl_2_ (200 mL) was cooled to −60 °C, and a solution of DMSO (114 mmol) in CH_2_Cl_2_ (20 mL) was added [21]. The mixture was stirred for 10 min at the same temperature, then a solution of dihydroartemisilacton (12 mmol) in CH_2_Cl_2_ (30 mL) was added. The reaction mixture was stirred for 60 min at the same temperature, followed by the addition of Et3N (288 mmol), and the mixture was stirred for additional 20 min (the temperature was gradually increased to room temperature). After that, H_2_O (300 mL) was added to the reaction mixture, and partitioned with ether (3 × 300 mL). The combined ethereal extract was further washed by water (3 × 300 mL), and dried. The solvent was distilled off under vacuo, and the residue was chromatographed on a SiO_2_ column at a ratio of compound: adsorbent 1:25. The reaction products were eluted with mixtures of petroleum ether and ethyl acetate (40:1/25:1/3:1). The structures of substrates were determined by MS and NMR. Arteannuin I**/**J were used in the present experiment.

### 3.3. Plant Cell Cultures

The *A. annua* cells were sub-cultured routinely every 2 weeks using MS medium containing vitamin C (50 mg/L), 1-naphthylacetic acid (NAA, 0.5 mg/L) and 6-benzylaminopurine (6-BA, 1 mg/L), and transplanted to a 500-mL conical flask containing 200 mL of medium, and then cultured on a rotary shaker (120 rpm) for 10 days at 25 °C under the light and dark conditions. 

### 3.4. Detection of Secondary Metabolites after Feeding of Arteannuin I/J

Three groups of experiments were taken out to check whether a biosynthetic process occurred. Group I contained cultured cells and arteannuin I/J; group II was the first control experiment, consisting of cultured cells without arteannuin I/J; group III was the second control experiment, in which only arteannuin I/J existed. The procedure of experiment group I was as follows: arteannuin I/J (1, 2, 3, 5, 7 and 9 mg) in DMSO (0.1 mL) was administered to the flask containing the suspended cells (precultured for 10 days) of *A. annua* and then cocultured at 25 °C on a rotary shaker in the dark (110 rpm) for different times (0, 6, 12, 24, 36, 48, 60 and 72 h). After incubation, cells and medium were separated by filtration with suction. Filtered medium was extracted with EtOAc and concentrated to dryness. The cells were also extracted with EtOAc for 24 h and sonicated for 20 min. The EtOAc fraction was also concentrated to dryness. Two EtOAc fractions were analyzed by TLC, HPLC-ELSD, and GC-MS, respectively. To group II, 0.1 mL of DMSO was added to the medium. For group III, arteannuin I/J in DMSO (0.1 mL) were administered to the medium. Extraction and analysis processes of the two control experiments were the same as those described above. 

### 3.5. Isolation and Structure Elucidation of Compound ***1***–***4***

The biosynthesis products in the medium (200 mL) were extracted with ethyl acetate (3 × 200 mL). Combined extracts were concentrated under reduced pressure yielding F0 1.02 g of a dark brown crude extract. Crude extract was subjected to purification by flash chromatography over silica gel (elution gradient: petroleum ether/ethyl acetate 10:1 → 6:1 → 4:1 → 3:1 → 2:1 → 1:1), giving 5 fractions named F1, F2, F3, F4 and F5. Fraction F1 (216.3 mg) was arteannuin I/J. Fraction F2–F5 were 38.04 mg, 28.6 mg, 50.4 mg and 86.4 mg, respectively. With a similar purification processes, F2–F5 yielded 1 (2.5 mg), 2 (5.2 mg), 3 (6.7 mg) and 4 (9.2 mg) (HPLC sequence, acetonitrile: water = 40/60, 35/65, 35/65, 30/70, respectively). 

*6-Hydroxy arteannuin I* (**1**): Yellow oil, HRESIMS: *m*/*z* 273.1444 [M + Na]^+^ C_15_H_22_O_3_Na. ^1^H-NMR (500 MHz, Chloroform-d) δ 5.08 (s, 1H), 4.85 (d, *J* = 1.7 Hz, 1H), 4.76 (d, *J* = 12.1 Hz, 1H), 2.57 (d, *J* = 7.1 Hz, 1H), 2.32–2.25 (m, 1H), 2.24–2.19 (m, 1H), 2.04–2.01 (m, 1H), 2.01–1.98 (m, 1H), 1.82 (d, *J* = 4.2 Hz, 1H), 1.79–1.75 (m, 1H), 1.73 (dt, *J* = 5.6, 2.8 Hz, 1H), 1.71–1.68 (m, 1H), 1.58 (s, 1H), 1.52 (d, *J* = 3.8 Hz, 1H), 1.44–1.41 (m, 1H), 1.29 (d, *J* = 7.2 Hz, 3H), 1.24 (m, 1H), 0.94 (d, *J* = 6.2 Hz, 3H). ^13^C-NMR (126 MHz, Chloroform-d) δ 172.79 (C-12), 145.35 (C-4), 105.77 (C-15); 76.96 (C-5), 71.71 (C-6), 51.20 (C-7), 48.73 (C-11), 38.64 (C-10), 30.15 (C-9), 29.85 (C-3), 28.44 (C-2), 28.22 (C-8), 27.75 (C-1), 19.90 (C-14), 8.90 (C-13);

*1-Hydroxy arteannuin I* (**2**): Yellow oil, HRESIMS: *m*/*z* 273.1445 [M + Na]^+^ C_15_H_22_O_3_Na. ^1^H-NMR (300 MHz, Chloroform-d) δ 5.13 (d, *J* = 1.2 Hz, 1H), 4.91 (d, *J* = 12.3 Hz, 1H), 4.88 (d, *J* = 1.5 Hz, 1H), 2.64 (p, *J* = 7.2 Hz, 1H), 2.39 (dd, *J* = 9.4, 3.6 Hz, 1H), 2.34 (m, 1H), 2.18–2.10 (m, 1H), 2.10–2.00 (m, 1H), 1.93 (d, *J* = 6.5 Hz, 1H), 1.87 (dd, *J* = 11.8, 3.9 Hz, 1H), 1.78 (m, 1H), 1.68 (dt, *J* = 12.9, 3.4 Hz, 1H), 1.60 (s, 1H), 1.46 (dd, *J* = 7.6, 5.1 Hz, 1H), 1.40 (m, 1H), 1.30 (m, 1H), 1.24 (d, *J* = 7.2 Hz, 3H), 0.92 (d, *J* = 6.6 Hz, 3H). ^13^C-NMR (75 MHz, Chloroform-d) ppm: 174.33 (C-12), 144.39 (C-4), 106.69 (C-15); 76.17 (C-5), 73.36 (C-1), 51.29 (C-6), 40.23 (C-11), 37.74 (C-2), 34.72 (C-7), 31.56 (C-10), 29.82 (C-9), 29.57 (C-3), 22.51 (C-8), 14.36 (C-14), 13.51 (C-13);

*2-Hydroxy arteannuin J* (**3**): Yellow oil, HRESIMS: *m*/*z* 273.1627 [M + Na]^+^ C_15_H_22_O_3_Na. ^1^H-NMR (500 MHz, Chloroform-d) δ 5.57 (m, 1H), 4.84 (d, *J* = 11.1 Hz, 1H), 4.18 (d, *J* = 4.1 Hz, 1H), 2.78 (p, *J* = 7.2 Hz, 1H), 2.38 (dt, *J* = 11.0, 3.6 Hz, 1H), 1.98–1.91 (m, 1H), 1.89–1.83 (overlap, 4H), 1.80–1.74 (m, 1H), 1.60 (s, 1H), 1.58 (d, *J* = 4.0 Hz, 1H), 1.33 (dd, *J* = 9.9, 4.1 Hz, 1H), 1.31–1.28 (m, 1H), 1.25 (d, *J* = 7.3 Hz, 3H), 1.07 (dd, *J* = 12.0, 4.2 Hz, 1H), 0.98 (d, *J* = 6.2 Hz, 3H). ^13^C-NMR (126 MHz, Chloroform-d) δ 175.14 (C-12), 137.94 (C-4), 124.16 (C-3), 74.92 (C-5), 65.48 (C-2), 50.27 (C-1), 40.87 (C-11), 38.46 (C-7), 35.88 (C-6), 34.96 (C-9), 28.09 (C-10), 23.11 (C-8), 20.17 (C-14), 18.44 (C-15), 13.45 (C-13);

*14-Hydroxy arteannuin J* (**4**): Yellow oil, HRESIMS: *m*/*z* 273.1433 [M + Na]^+^ C_15_H_22_O_3_Na. ^1^H-NMR (500 MHz, Chloroform-d) δ 5.39 (m, 1H), 4.95 (d, *J* = 10.8 Hz, 1H), 3.69 (dd, *J* = 10.8, 3.3 Hz, 1H), 3.53 (dd, *J* = 10.8, 5.8 Hz, 1H), 2.77 (p, *J* = 7.2 Hz, 1H), 2.25 (overlap, 2H), 2.19 (dt, *J* = 10.8, 3.7 Hz, 1H), 2.01 (m, 1H), 1.98–1.94 (m, 1H), 1.90–1.85 (m, 1H), 1.85–1.81 (m, 1H), 1.79 (dd, *J* = 2.4, 1.2 Hz, 3H), 1.61–1.57 (m, 1H), 1.52 (m, 1H), 1.39 (dd, *J* = 13.2, 3.8 Hz, 1H), 1.25 (d, *J* = 7.3 Hz, 4H). ^13^C-NMR (126 MHz, Chloroform-d) δ 175.43 (C-12), 132.08 (C-4), 122.77 (C-3), 75.28 (C-5), 65.68 (C-2), 40.80 (C-11), 40.40 (C-1), 38.73 (C-7), 36.77 (C-10), 36.23 (C-6), 29.31 (C-9), 27.12 (C-14), 22.76 (C-8), 18.43 (C-15), 13.48 (C-13).

## 4. Conclusions

In summary, we investigated the biosynthesis reactions of arteannuin I/J in suspension-cultured cells of *A. annua*. Four new compounds, namely, 6-hydroxy arteannuin I (**1**), 1-hydroxy arteannuin I (**2**), 2-hydroxy arteannuin J (**3**), and 14-hydroxy arteannuin J (**4**), were isolated and their structure was elucidated. Results from the spontaneous oxidation experiment indicated that these hydroxylation products of arteannuin I/J were enzyme-catalyzed, and that the enzymes of *A. annua* showed the abilities of substrate-selective and region-selective hydroxylation of the sesquiterpene lactone. Furthermore, enzyme activities were increased under light conditions, which promoted the conversion of arteannuin J to compound **3**. Additionally, the yield of artemisinin was enhanced by 50% and 80% compared to the control group in *A. annua* cultured cells under light and dark conditions, respectively. This indicated that arteannuin I/J might play key roles as precursors in artemisinin biosynthesis.

## Figures and Tables

**Table 1:** ^13^C and ^1^H-NMR data for compounds **1** and **2** (in CDCl_3_).

Position	1 ^a^		2 ^b^	
	δ_H_, mult. (*J* in Hz)	δ_C_	δ_H_, mult. (*J* in Hz)	δ_C_
1	1.76, m	27.75		73.36
2α	1.42, m	28.44	1.40, m	37.74
2β	1.69, m		2.16, m	
3α	2.29, m	29.85	2.34, m	29.57
3β	1.24, m		2.06, m	
4		145.35		144.39
5	4.76, d (12.1)	76.79	4.91, d (12.3)	76.17
6		71.71	1.87, dd (11.8, 3.9)	51.29
7	1.82, d (4.2)	51.20	2.39, dd (9.46, 3.6)	34.72
8α	2.02, m	28.22	1.78, m	22.51
8β	1.52, d (3.8)		1.30, m	
9α	1.73, dt (5.6, 2.8)	30.15	1.46, dd (7.6, 5.1)	29.82
9β	2.20, m		1.68, m	
10	2.00, m	38.64	1.93, d (6.5)	31.56
11	2.57, d (7.1)	48.73	2.64, p (7.2)	40.23
12		172.79		174.33
13	1.29, d (7.2)	8.90	1.24, d (7.2)	13.54
14	0.94, d (6.2)	19.90	0.92, d (6.6)	14.36
15a	5.08, s	105.77	4.88, d (1.5)	106.69
15b	4.85, d (1.7)		5.13, d (1.2)	
OH	1.58, s		1.60, s	

^a 13^C (126 MHz) and ^1^H (500 MHz) NMR data for **1**; ^b 13^C (75 MHz) and ^1^H (300 MHz) NMR data for **2**.

**Table 2:** ^13^C (126 MHz) and ^1^H (500 MHz) NMR data for compounds **3** and **4** (in CDCl_3_).

Position	3		4	
	δ_H_, mult. (*J* in Hz)	δ_C_	δ_H_, mult. (*J* in Hz)	δ_C_
1	1.58, d (4.0)	50.27	2.19, dt (10.8, 3.7)	40.40
2	4.18, d (4.1)	65.48	2.25, (overlap)	27.12
3	5.57, m	124.16	5.39, m	122.77
4		145.35		132.08
5	4.84, d (11.1)	74.92	4.95, d (10.8)	75.28
6	2.38, dt (11.0, 3.6)	35.88	1.87, m	36.23
7	1.94, m	38.64	1.96, m	38.73
8α	1.76, m	23.11	1.83, m	22.76
8β	1.33, dd (9.9, 4.1)		1.39, dd (13.2, 3.8)	
9α	1.07, dd (12.0, 4.2)	34.96	1.25, d (7.3)	29.31
9β	1.86, (overlap)		2.01, m	
10	1.29, m	28.09	1.52, m	36.77
11	2.78, p (7.2)	40.87	2.77, p (7.2)	40.80
12		175.14		175.43
13	1.25, d (7.3)	13.45	1.25, d (7.3)	13.48
14a	0.98, d (6.2)	20.17	3.69, dd (10.8, 3.3)	65.67
14b			3.53, dd (10.8, 5.8)	
15	1.86, (overlap)	18.44	1.79, dd (2.4, 1.2)	18.43
OH	1.60, m		1.60, m	

## Supplementary Materials

Supplementary materials are available online. The ^1^H-NMR, ^13^C-NMR, HRESIMS, DEPT, HSQC, HMBC, and NOESY of four new compounds (Compounds **1**–**4**).
