# Supplementary material for: Four New Compounds Obtained from Cultured Cells of Artemisia annua
Source: Molecules. 2017 Dec 18;22(12):2264. doi: 10.3390/molecules22122264 (PMC6150029; doi:10.3390/molecules22122264)
Supplement: Supplementary file 1 [file molecules-22-02264-s001.pdf]

molecules

Supplementary Materials

Four New Compounds obtained from Cultured Cells of *Artemisia annua*

Jianhua Zhu [1,]*, Peijie Xiao [1], Minghua Qian [2], Chang Chen [2], Chuxin Liang [2], Jiachen Zi [1] and Rongmin Yu [1,2,]*

Figure legends

Figure S 1-18. HRESIMS, 1D and 2D NMR spectra of compound **1**
Figure S 19-36. HRESIMS, 1D and 2D NMR spectra of compound **2**
Figure S 37-54. HRESIMS, 1D and 2D NMR spectra of compound **3**
Figure S 55-72. HRESIMS, 1D and 2D NMR spectra of compound **4**

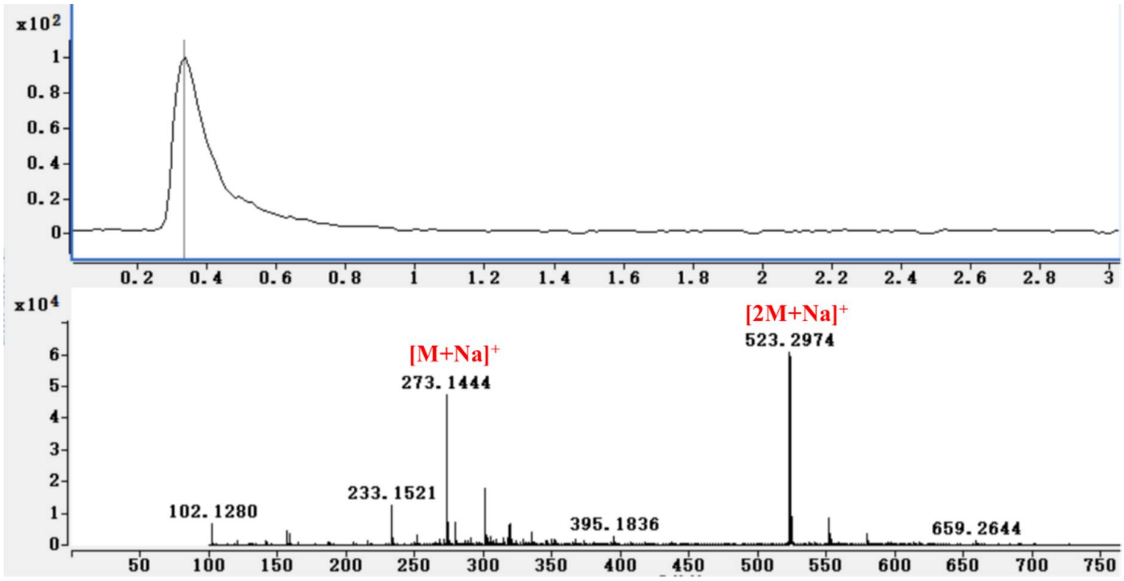

Figure S 1. HRESIMS spectrum of compound 1

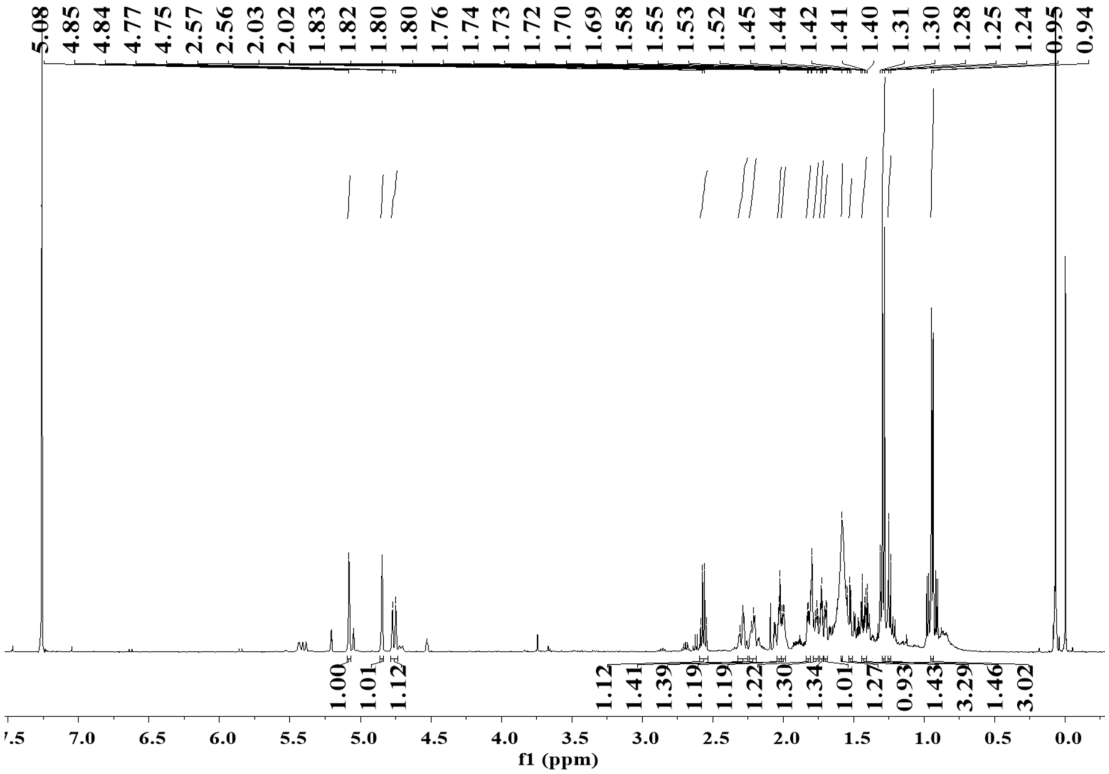

Figure S 2. ^1H NMR spectrum of compound 1 in $CDCl_3$

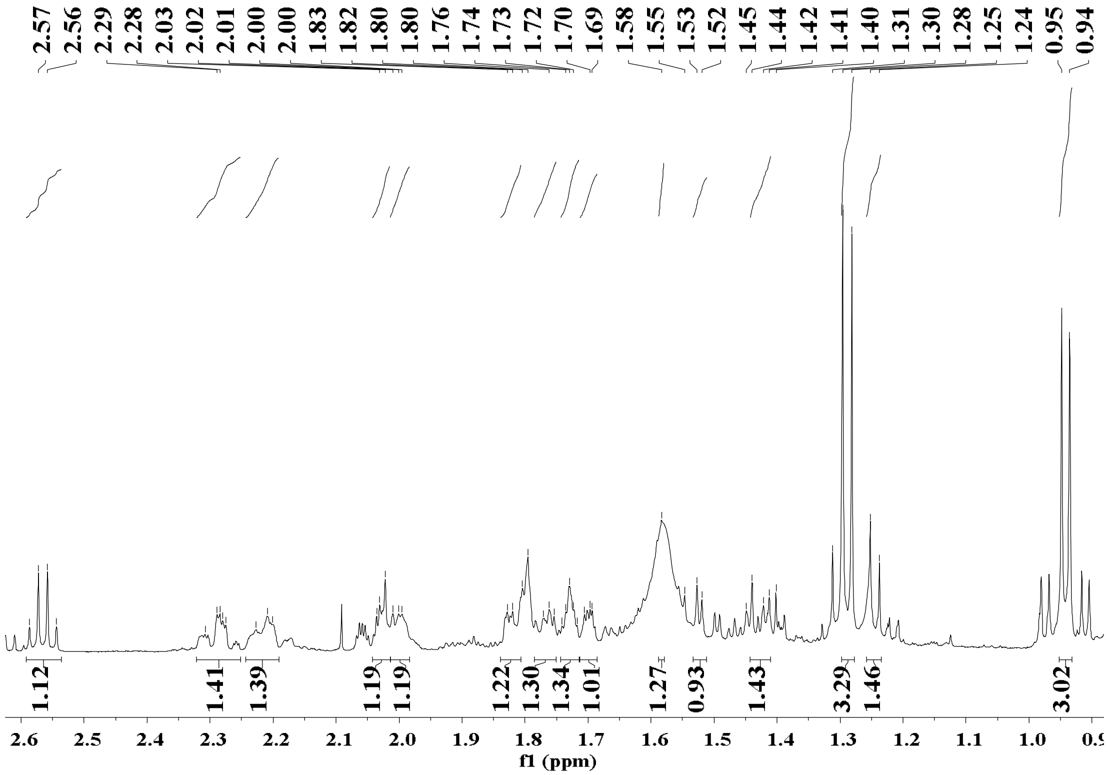

Figure S 3. A Segment of ^1H NMR spectrum of compound 1 in $CDCl_3$

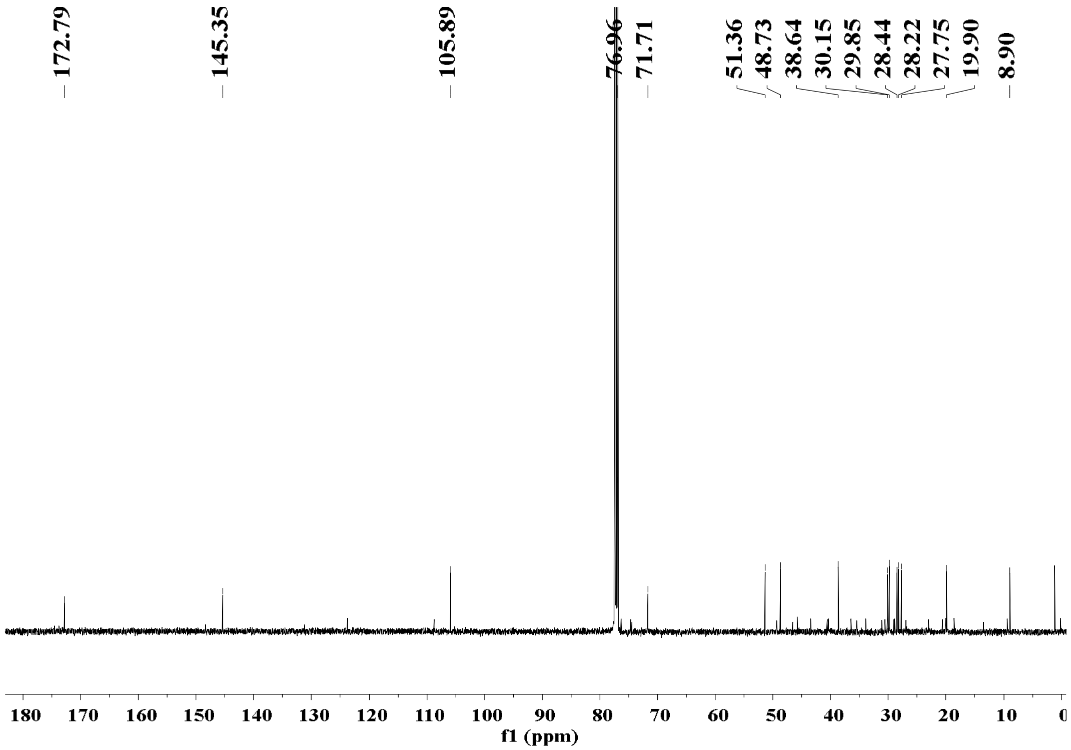

Figure S 4. ^{13}C **NMR spectrum of compound 1 in** $CDCl_3$

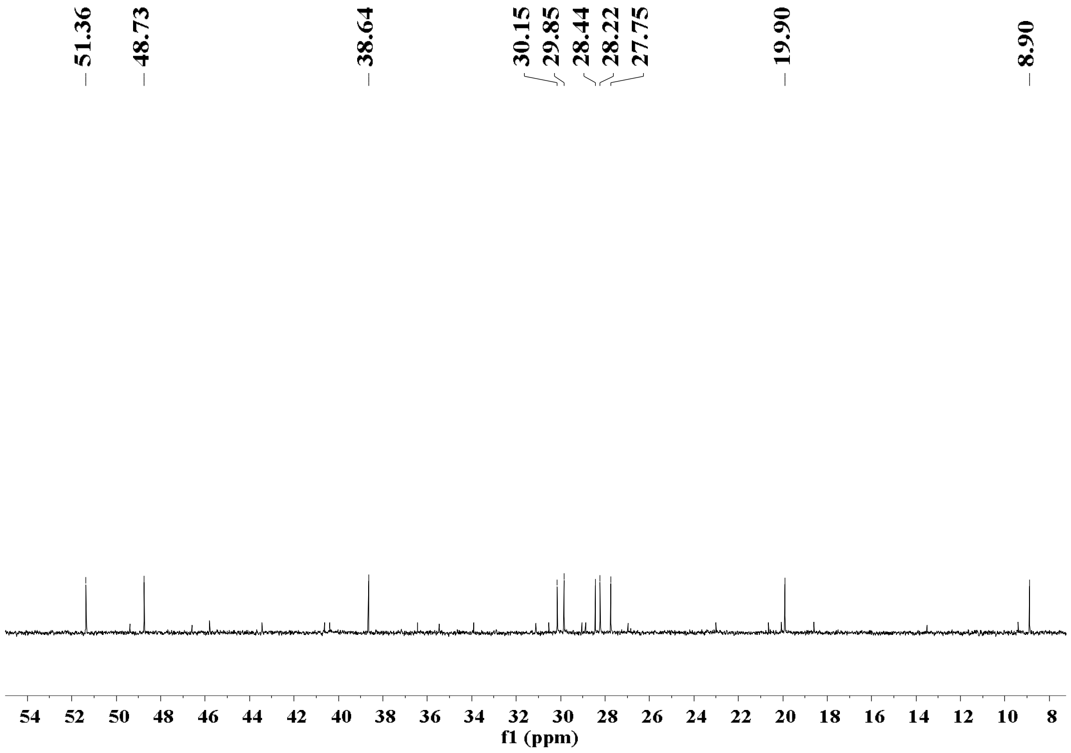

Figure S 5. A Segment of ^{13}C **NMR spectrum of compound 1 in** $CDCl_3$

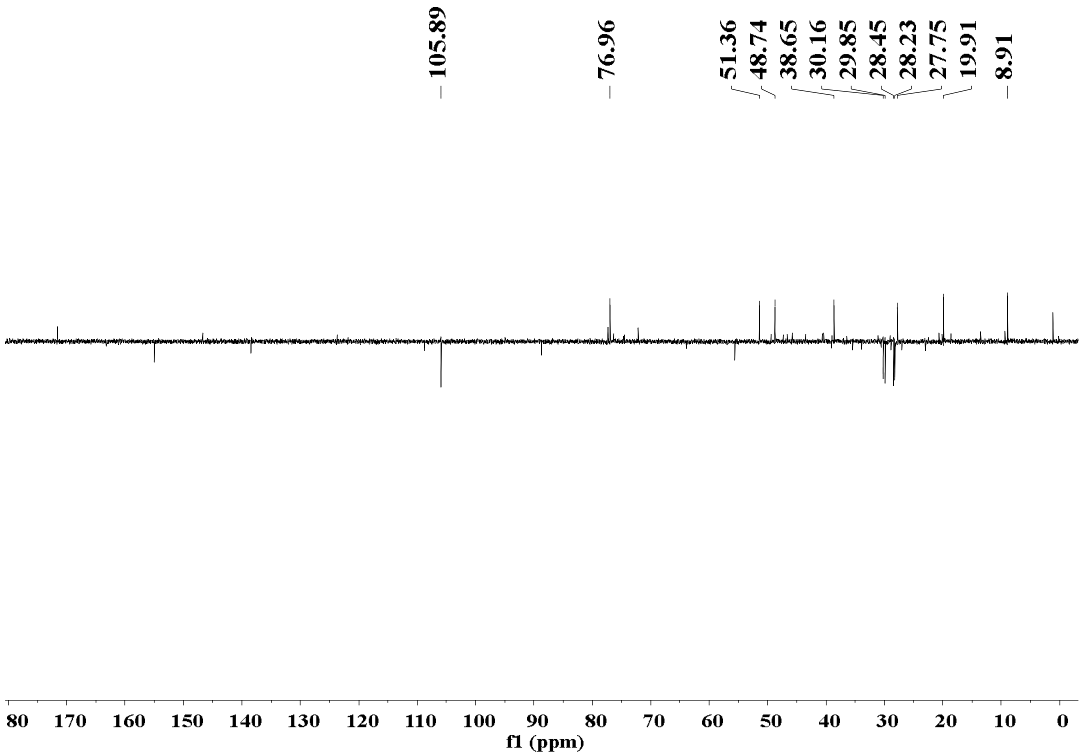

Figure S 6. DEPT135 of compound 1 in $CDCl_3$

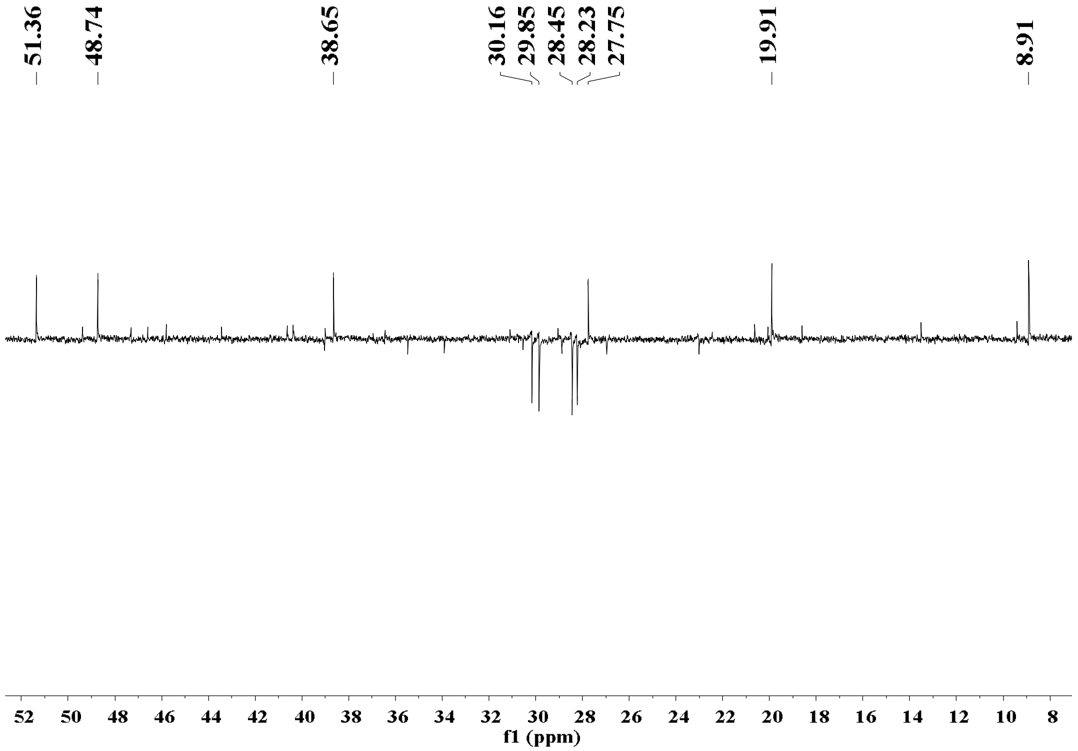

Figure S 7. A Segment of DEPT135 of compound 1

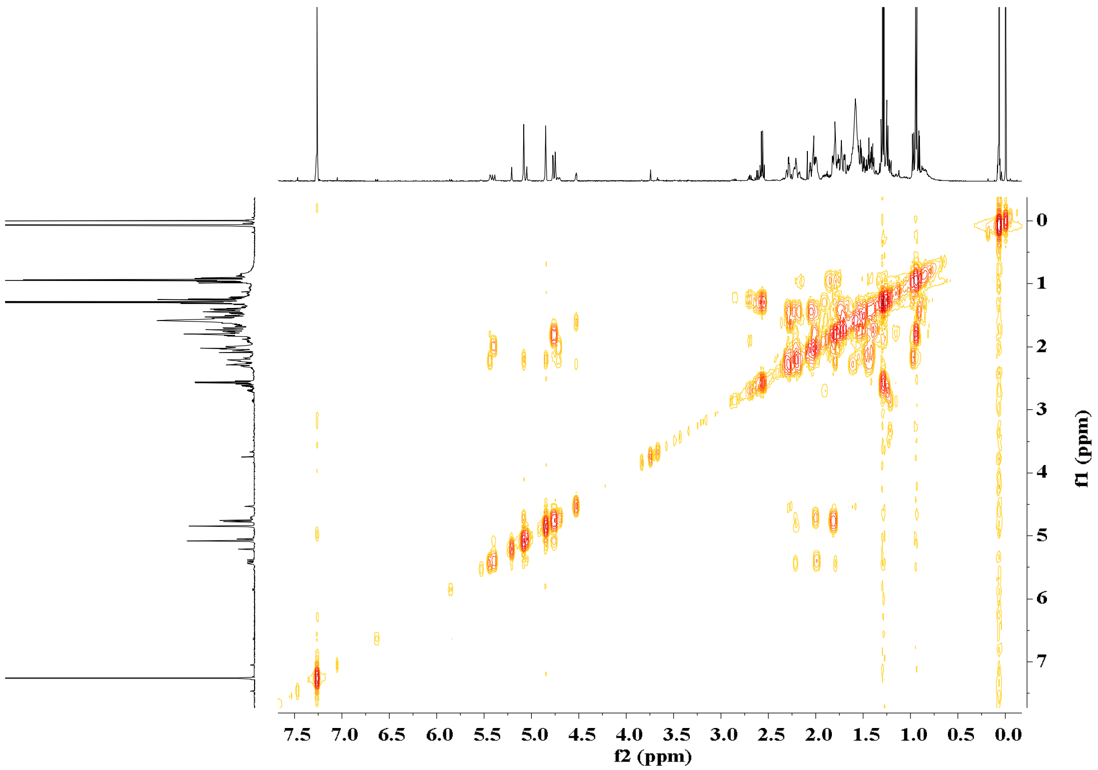

Figure S 8. ^{1}H-^{1}H COSY spectrum of compound 1 in $CDCl_3$

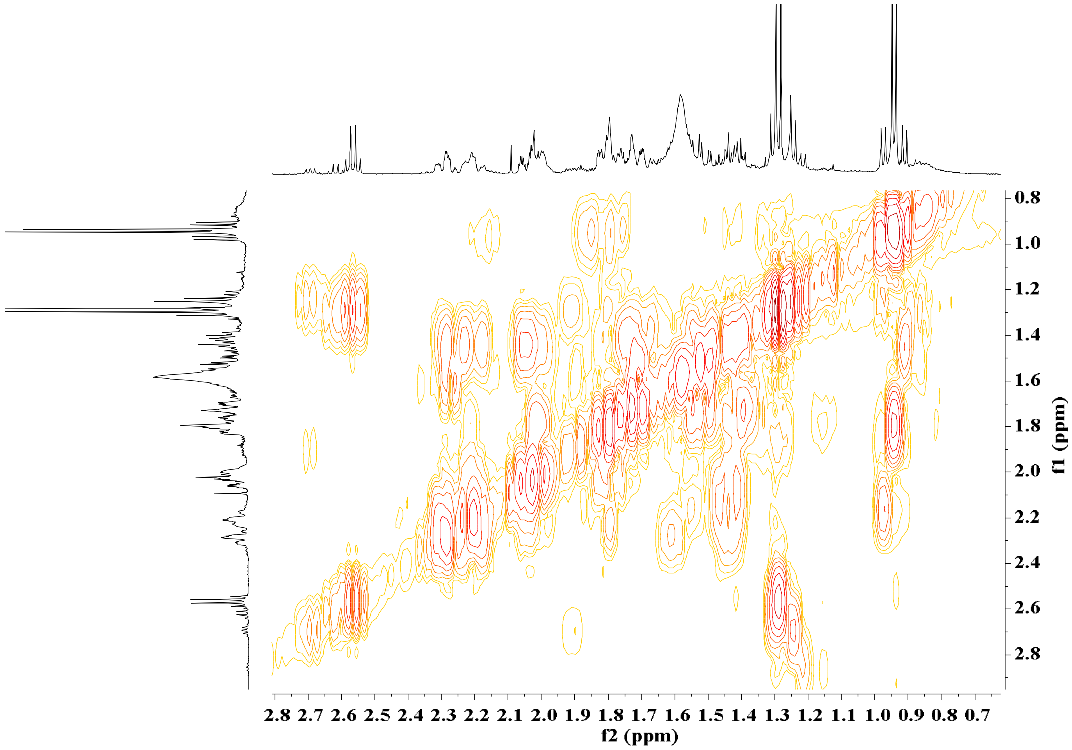

Figure S 9. A Segment of ^{1}H-^{1}H COSY spectrum of compound 1 in $CDCl_3$

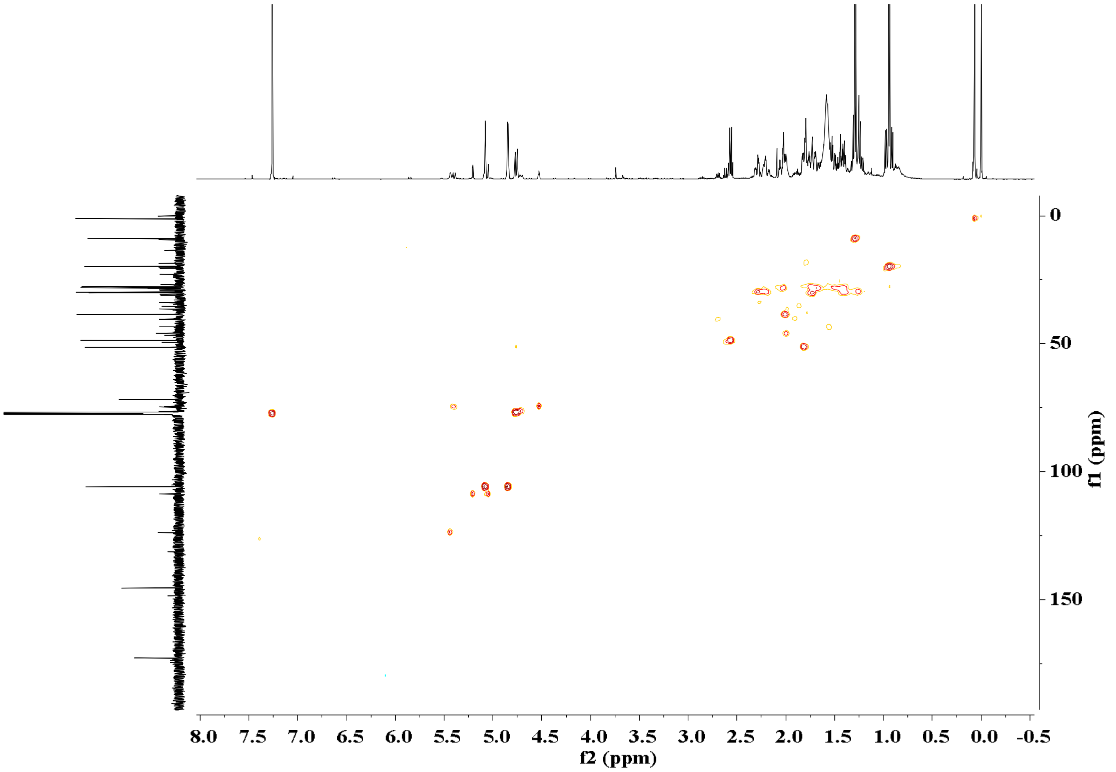

Figure S 10. HSQC spectrum of compound 1 in $CDCl_3$

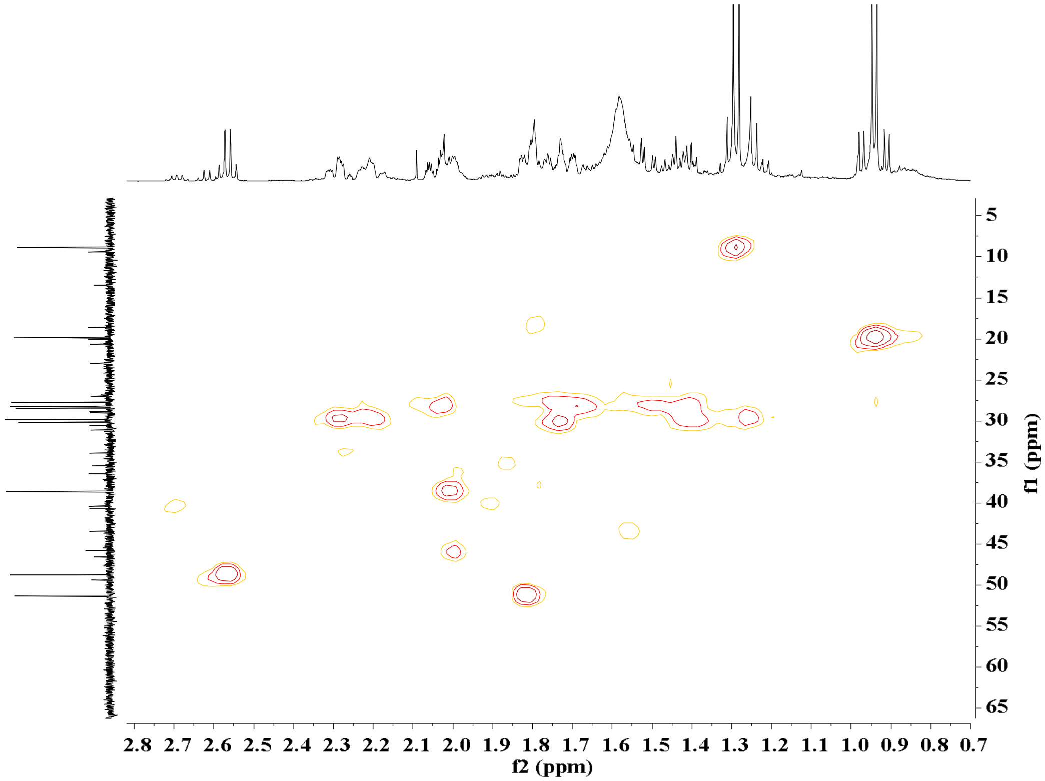

Figure S 11. A Segment of HSQC spectrum of compound 1 in $CDCl_3$

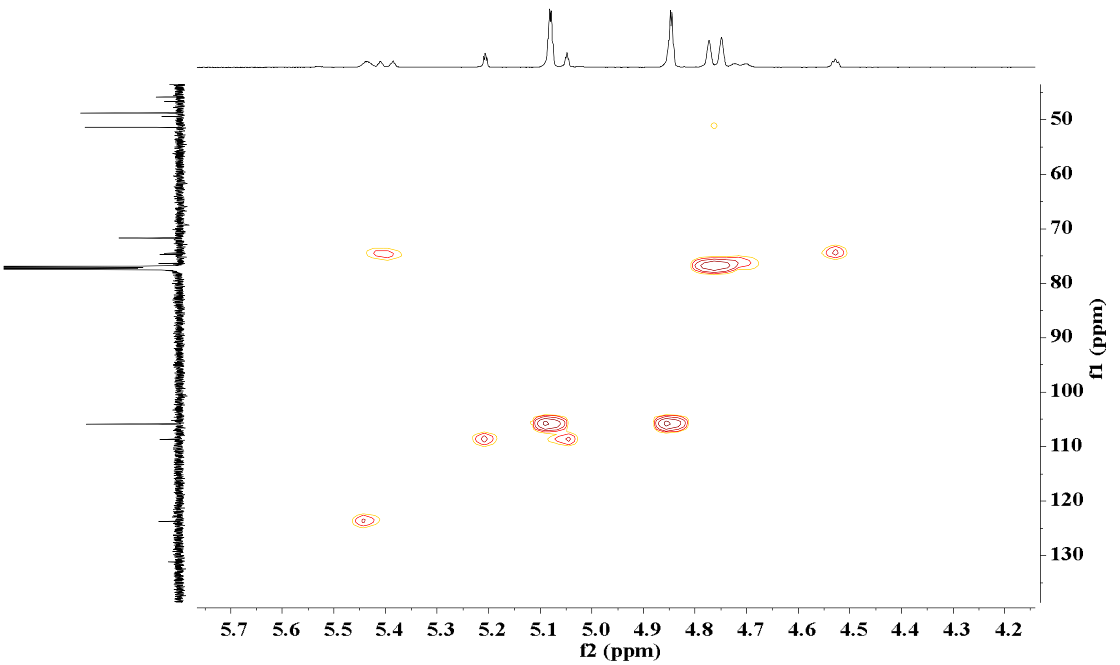

33

34 **Figure S 12. A Segment of HSQC spectrum of compound 1 in $CDCl_3$**

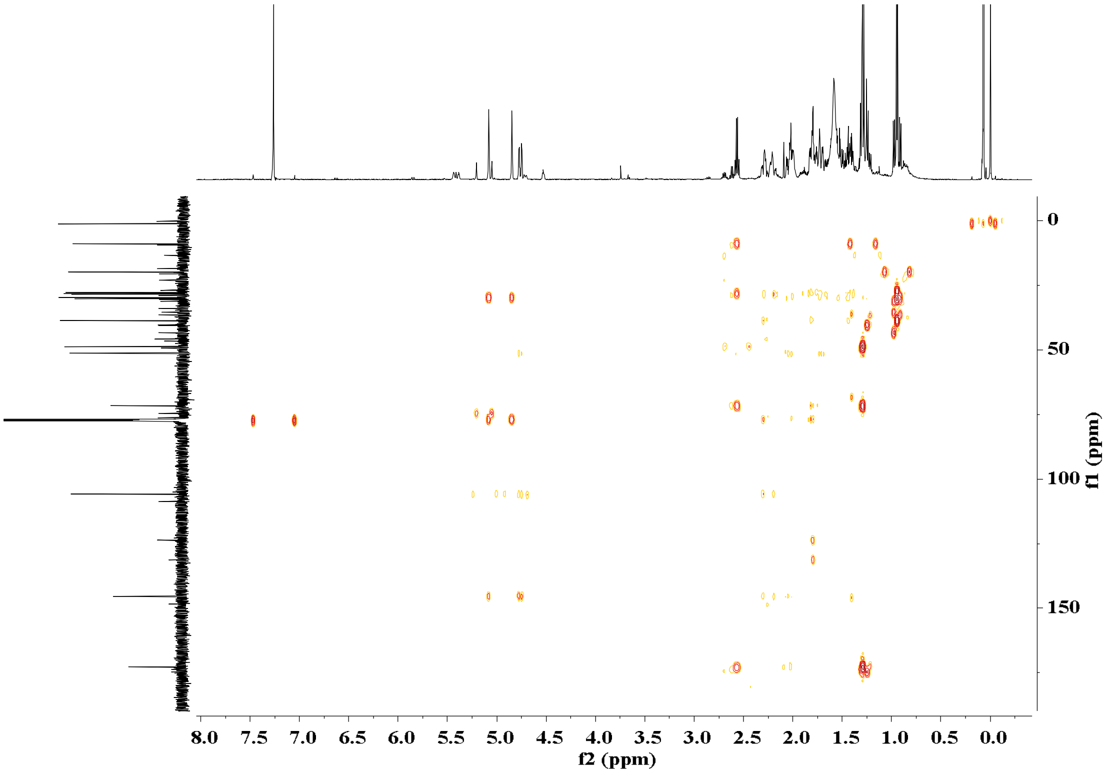

35

36 **Figure S 13. HMBC spectrum of compound 1 in $CDCl_3$**

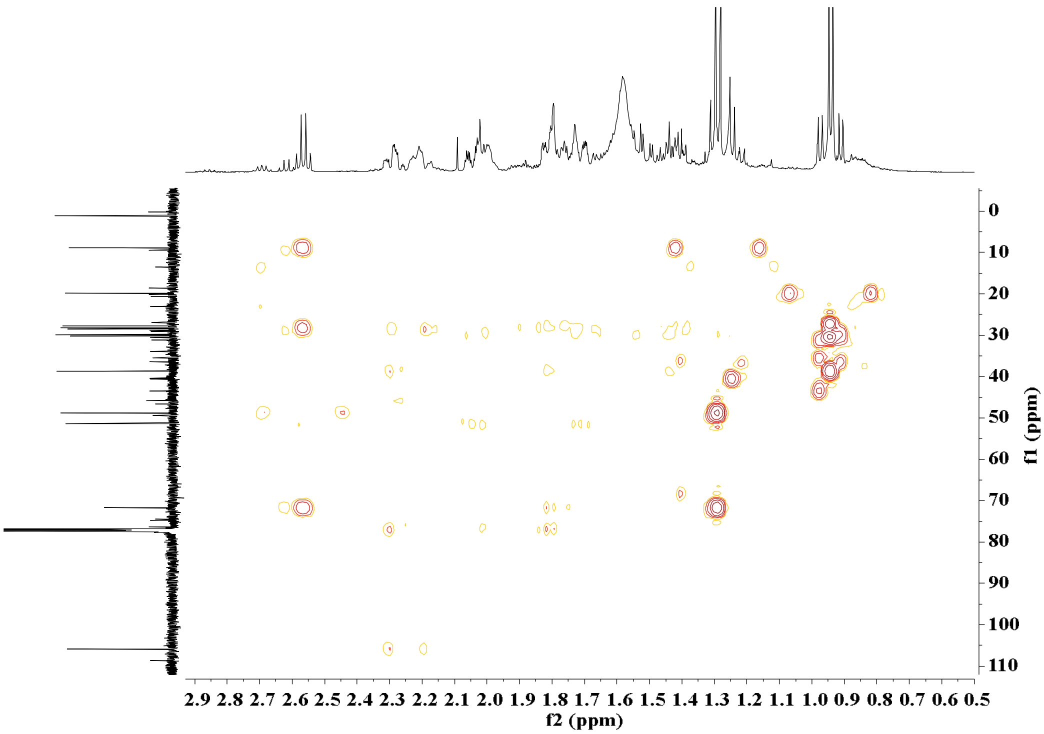

Figure S 14. A Segment of HMBC spectrum of compound 1 in $CDCl_3$

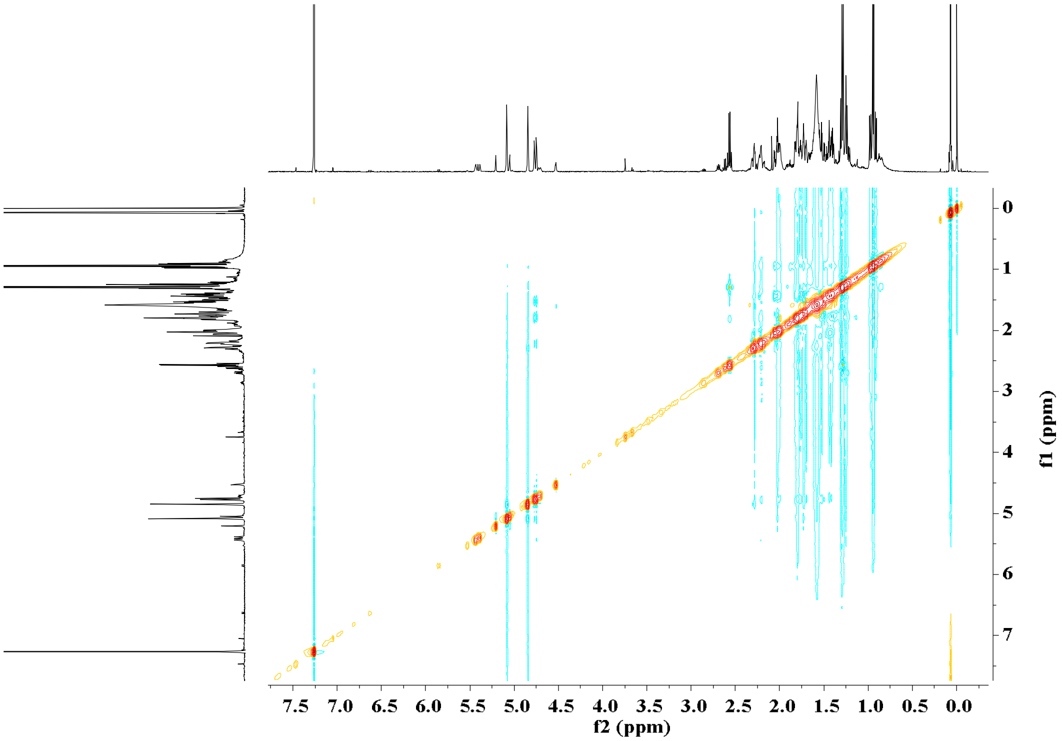

Figure S 15. NOESY spectrum of compound 1 in $CDCl_3$

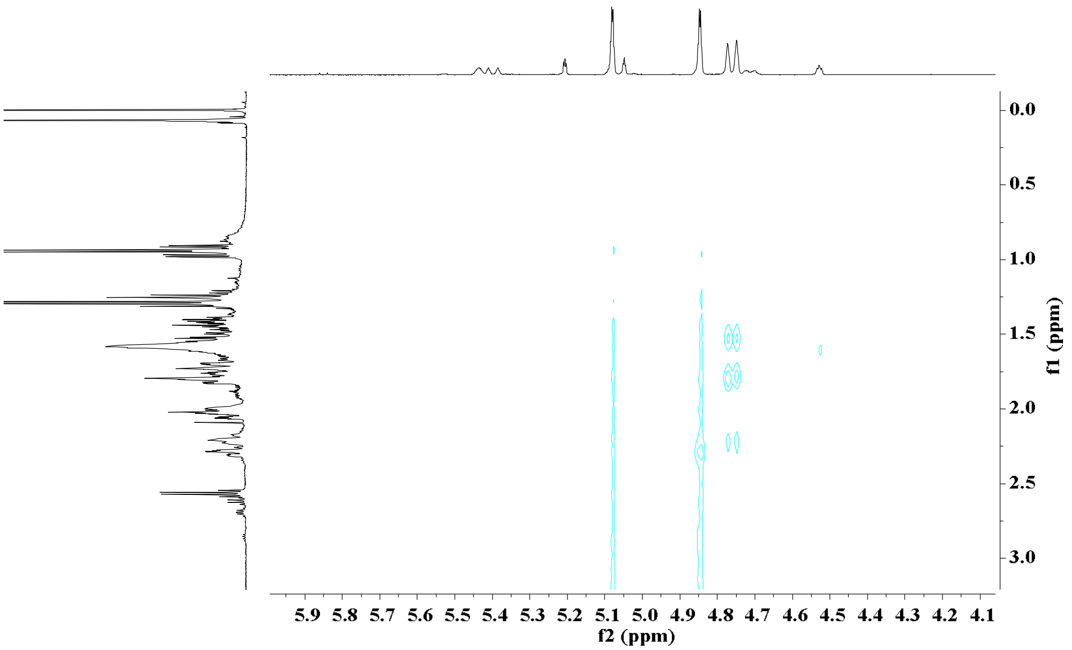

Figure S 16. A Segment of NOESY spectrum of compound 1 in $CDCl_3$

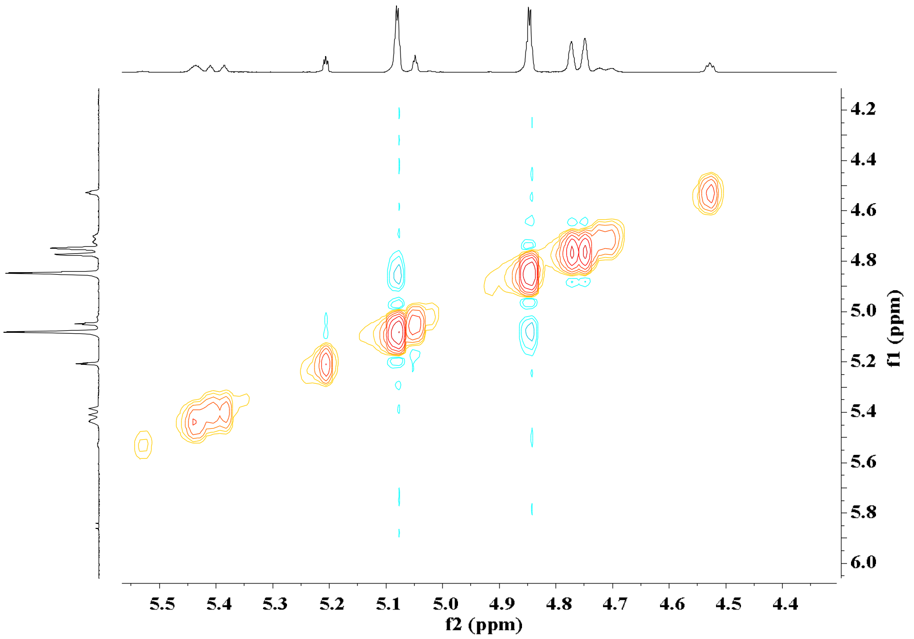

Figure S 17. A Segment of NOESY spectrum of compound 1 in $CDCl_3$

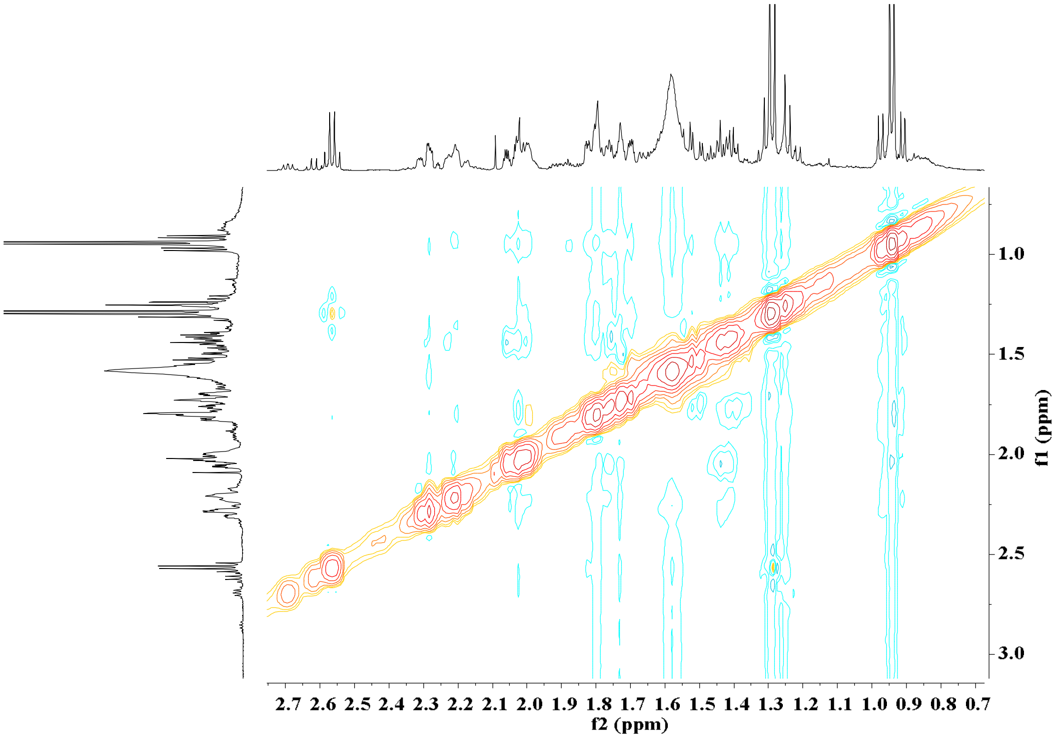

Figure S 18. A Segment of NOESY spectrum of compound 1 in $CDCl_3$

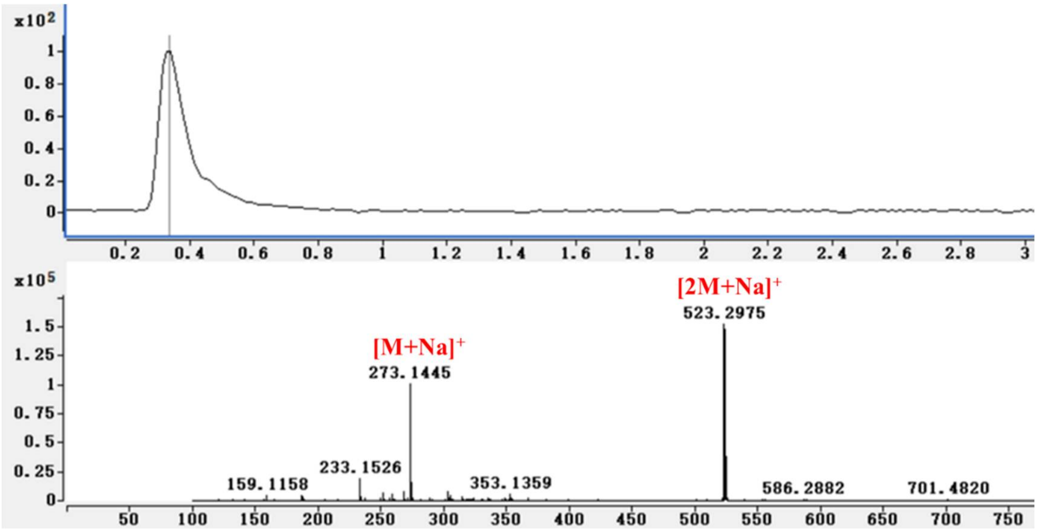

Figure S 19. HRESIMS spectrum of compound 2

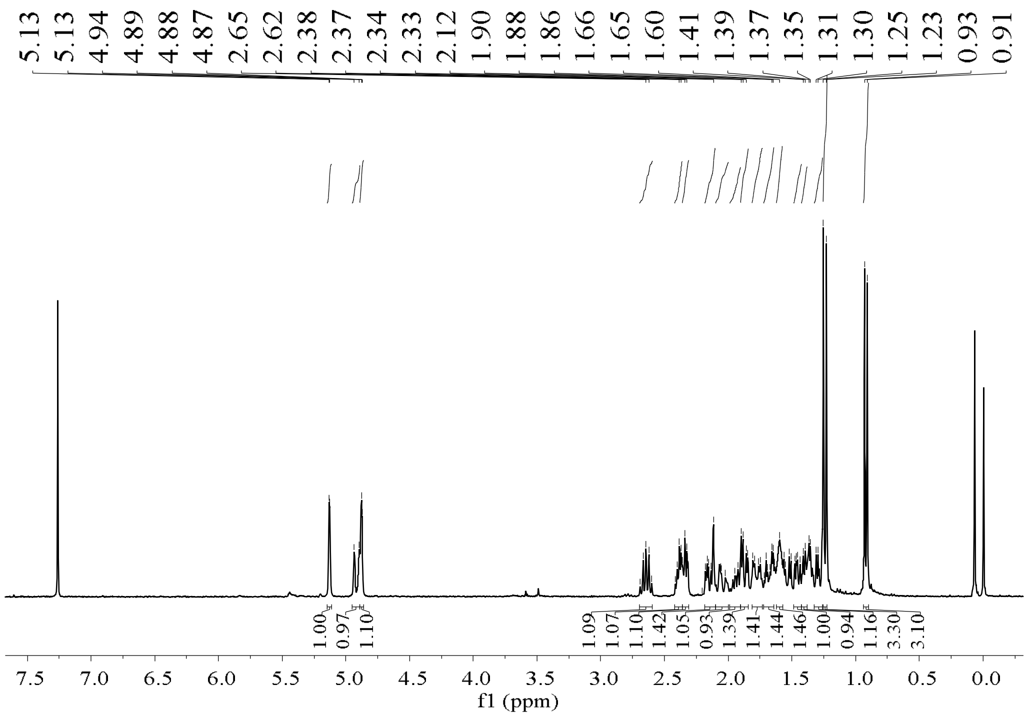

Figure S 20. ^{1}**H NMR spectrum of compound 2 in** $CDCl_3$

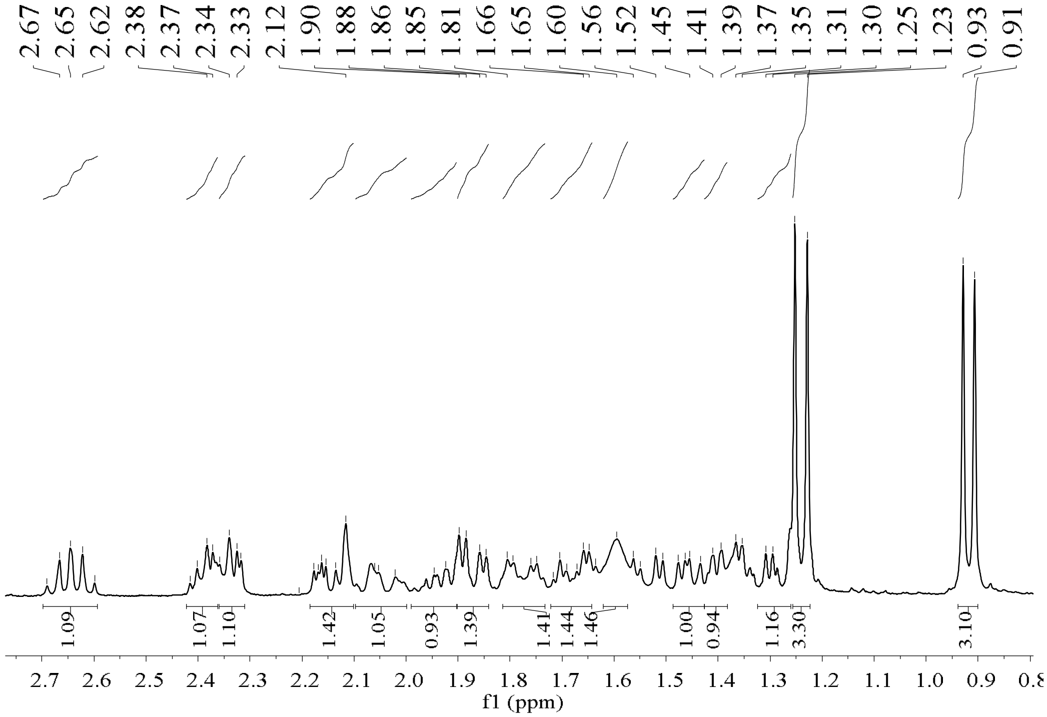

Figure S 21. Enlarged ^{1}**H NMR spectrum of compound 2 in** $CDCl_3$

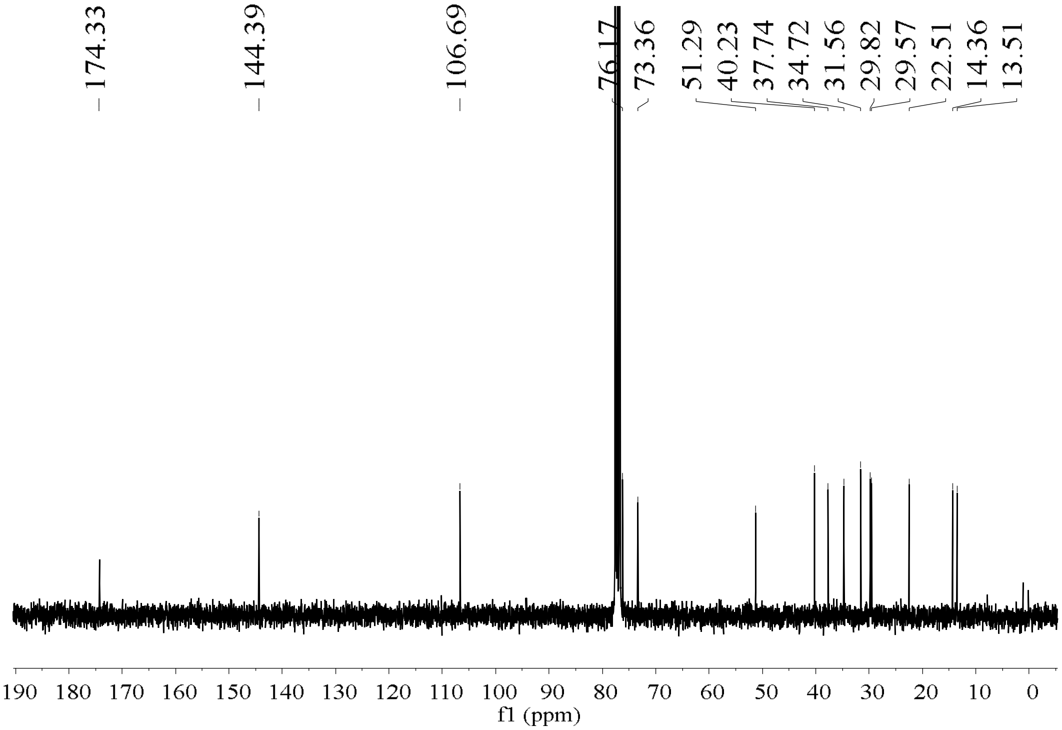

Figure S 22. ^{13}C NMR spectrum of compound 2 in $CDCl_3$

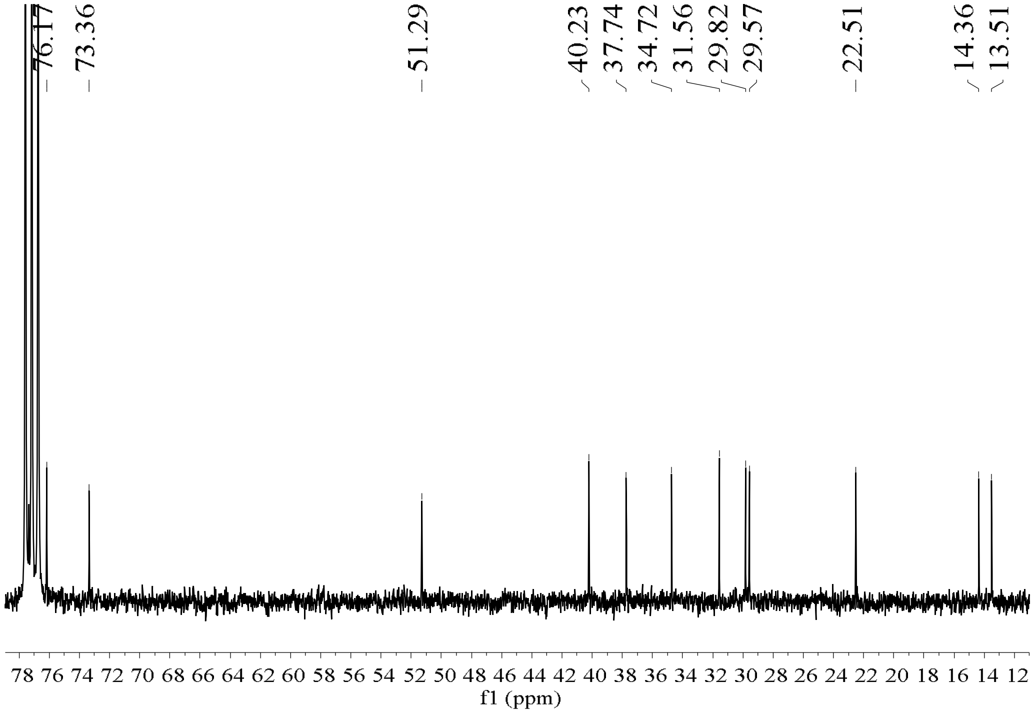

Figure S 23. A Segment of ^{13}C NMR spectrum of compound 2 in $CDCl_3$

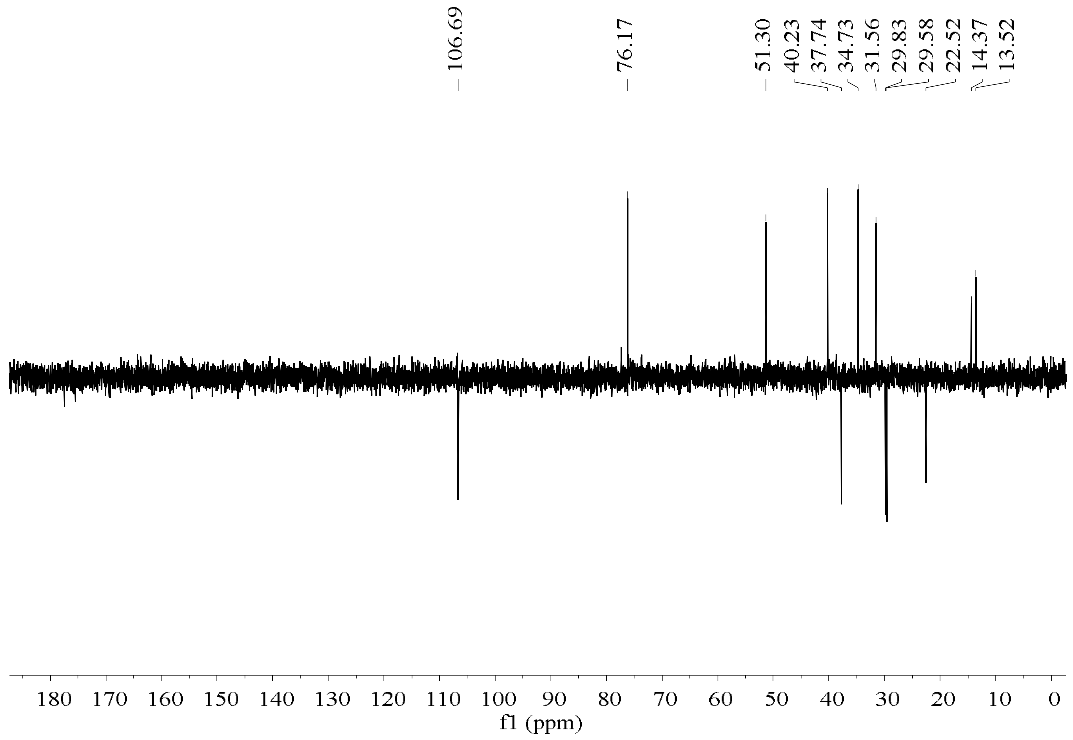

Figure S 24. DEPT135 of compound 2 in $CDCl_3$

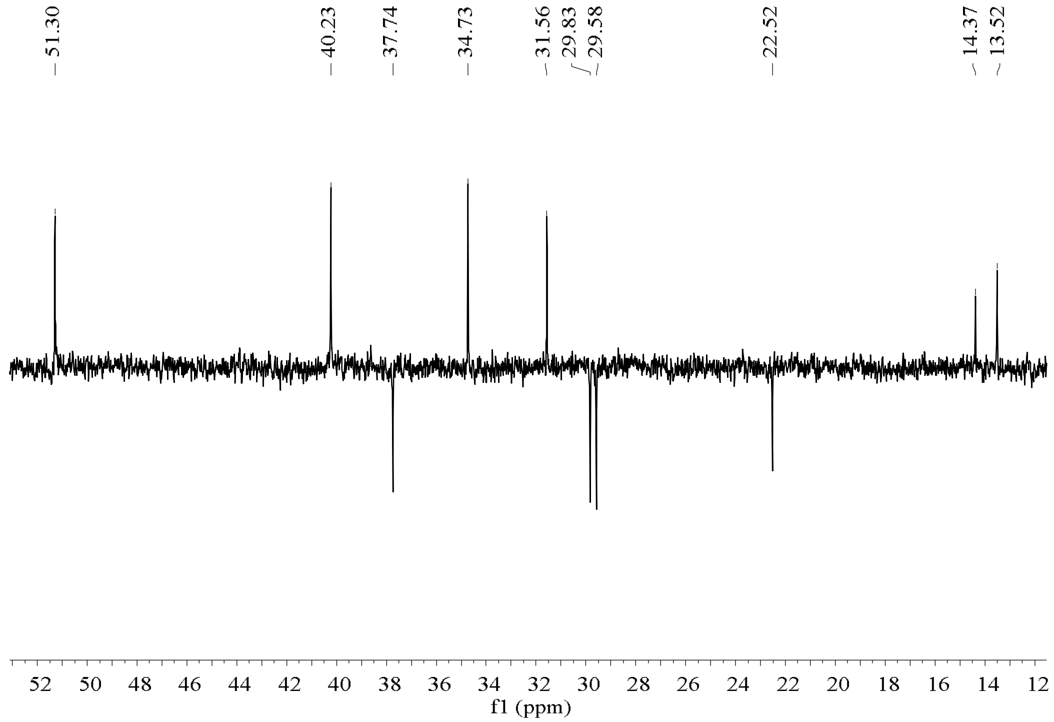

Figure S 25. A Segment of DEPT135 of compound 2 in $CDCl_3$

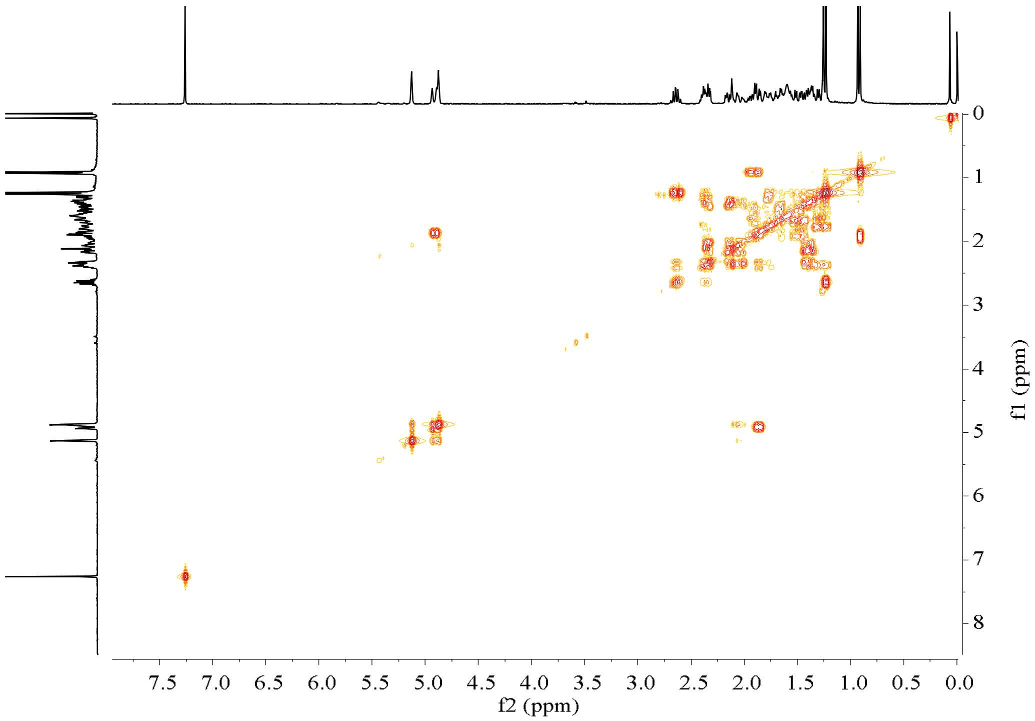

Figure S 26. ^{1}H-^{1}H COSY spectrum of compound 2 in $CDCl_3$

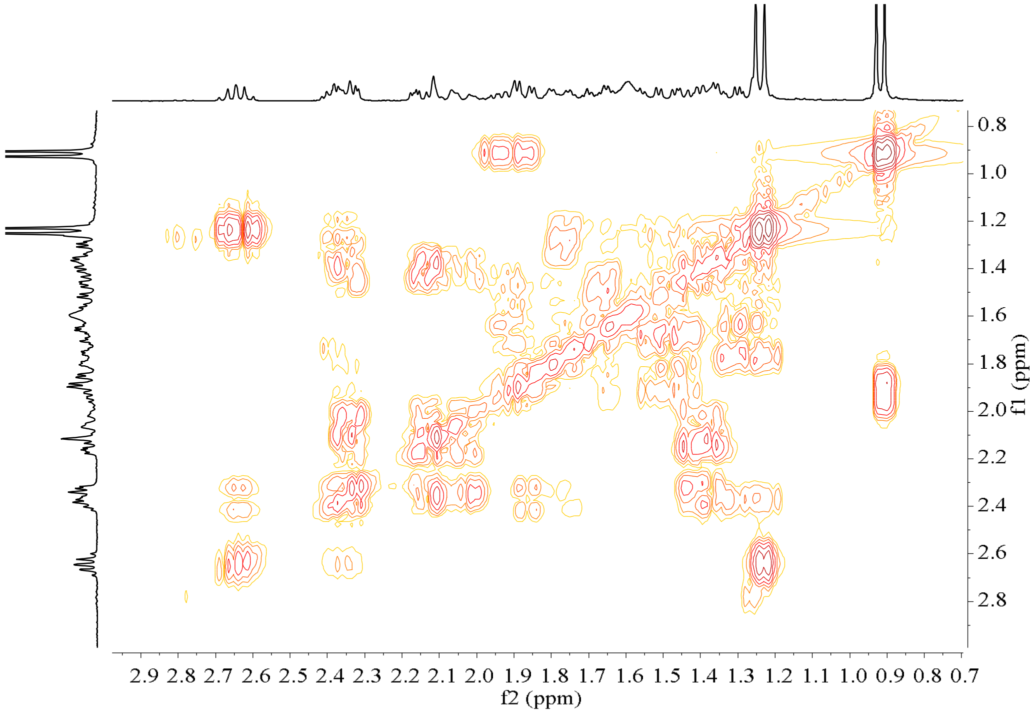

Figure S 27. A Segment of ^{1}H-^{1}H COSY spectrum of compound 2 in $CDCl_3$

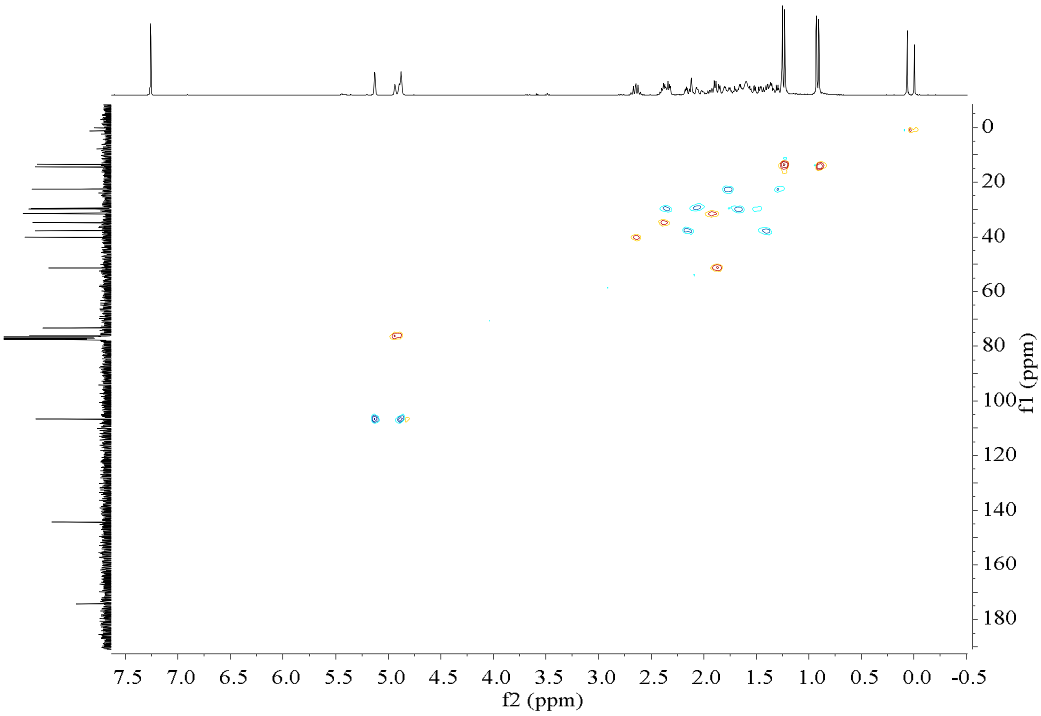

Figure S 28. HSQC spectrum of compound 2 in $CDCl_3$

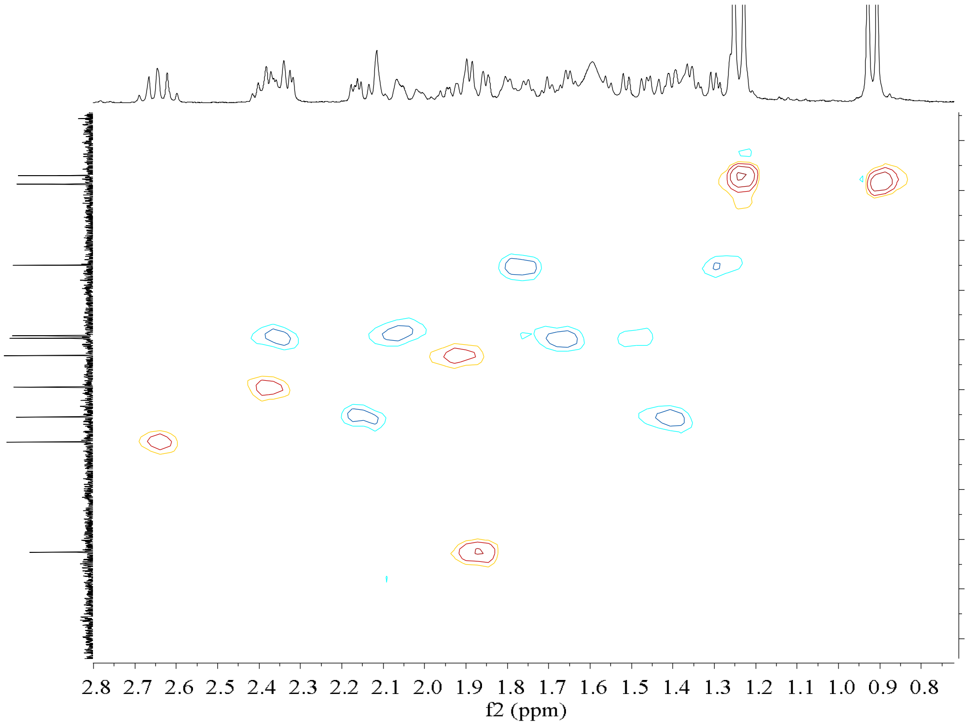

Figure S 29. A Segment of HSQC spectrum of compound 2 in $CDCl_3$

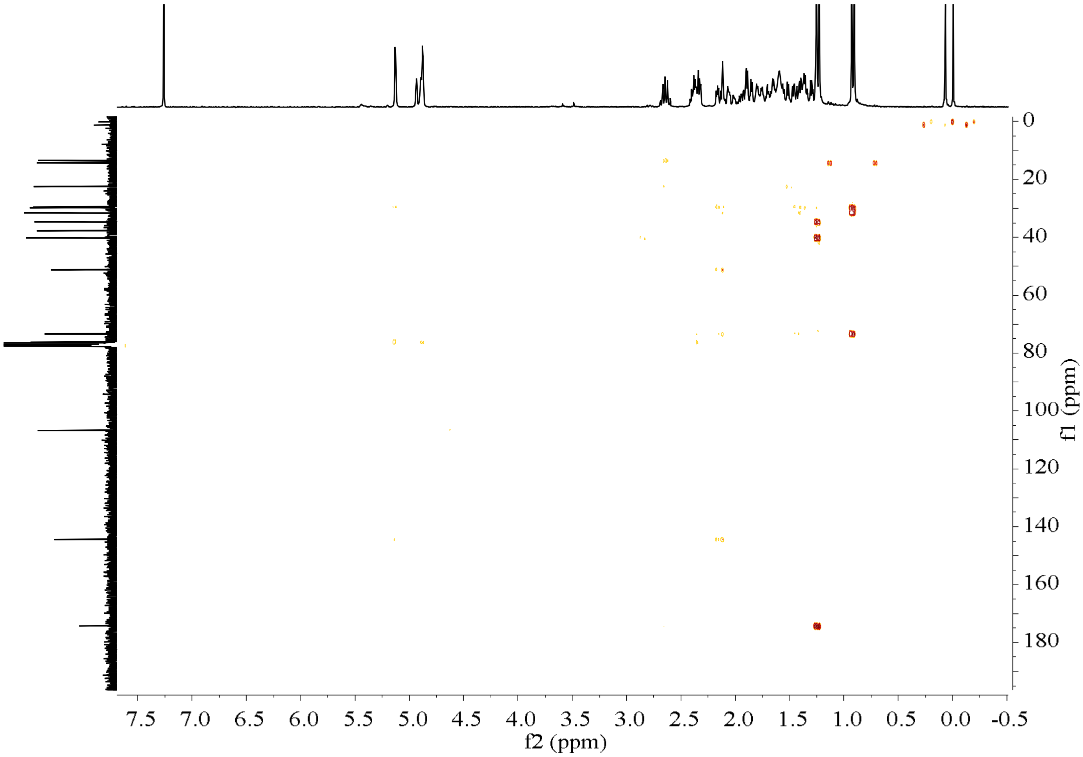

Figure S 30. HMBC spectrum of compound 2 in $CDCl_3$

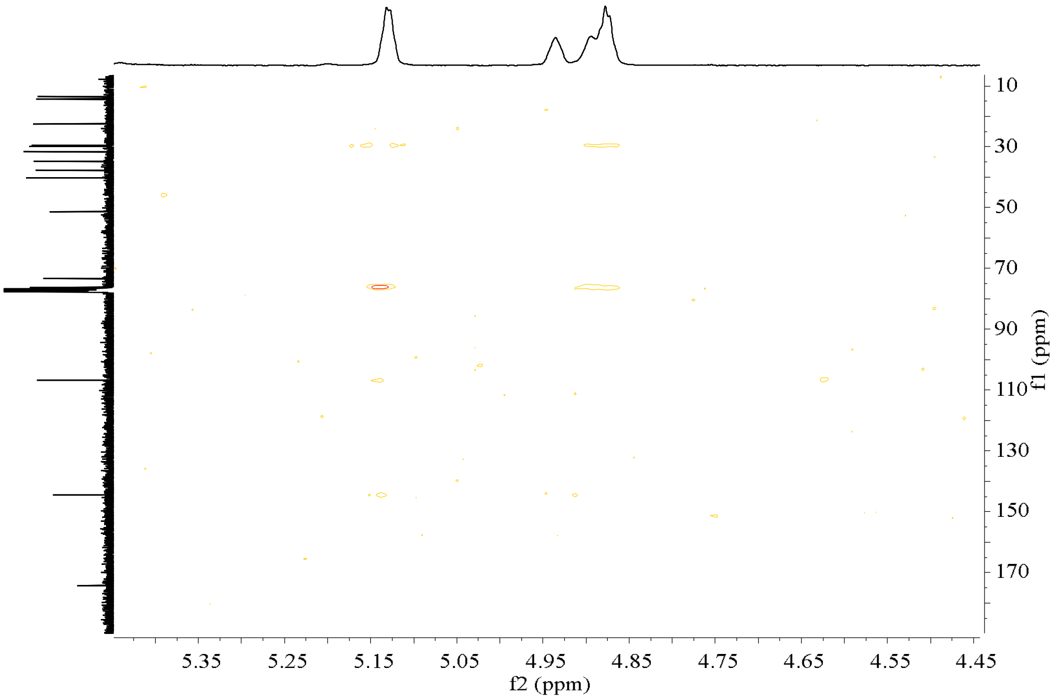

Figure S 31. A Segment of HMBC spectrum of compound 2 in $CDCl_3$

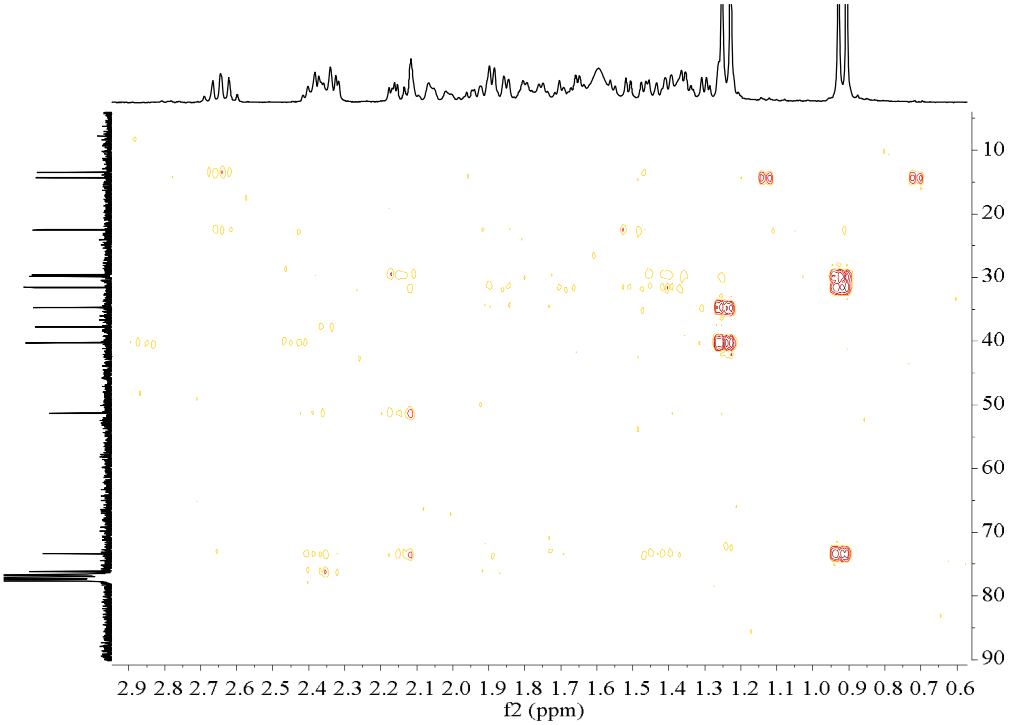

Figure S 32. A Segment of HMBC spectrum of compound 2 in $CDCl_3$

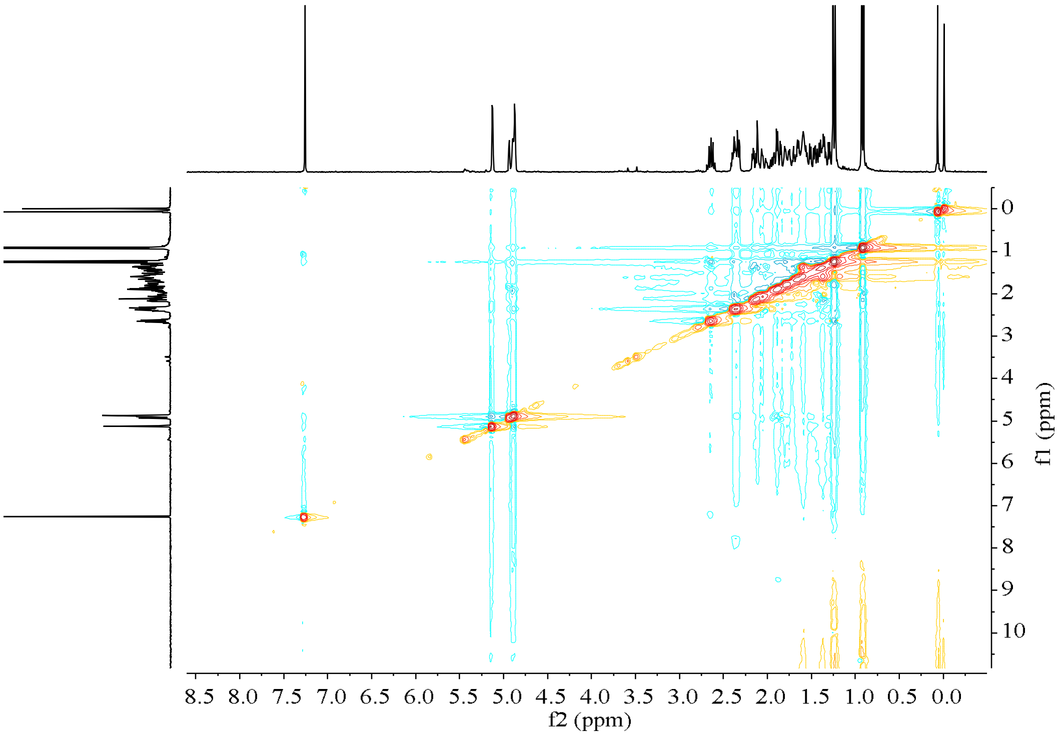

Figure S 33. NOESY spectrum of compound 2 in $CDCl_3$

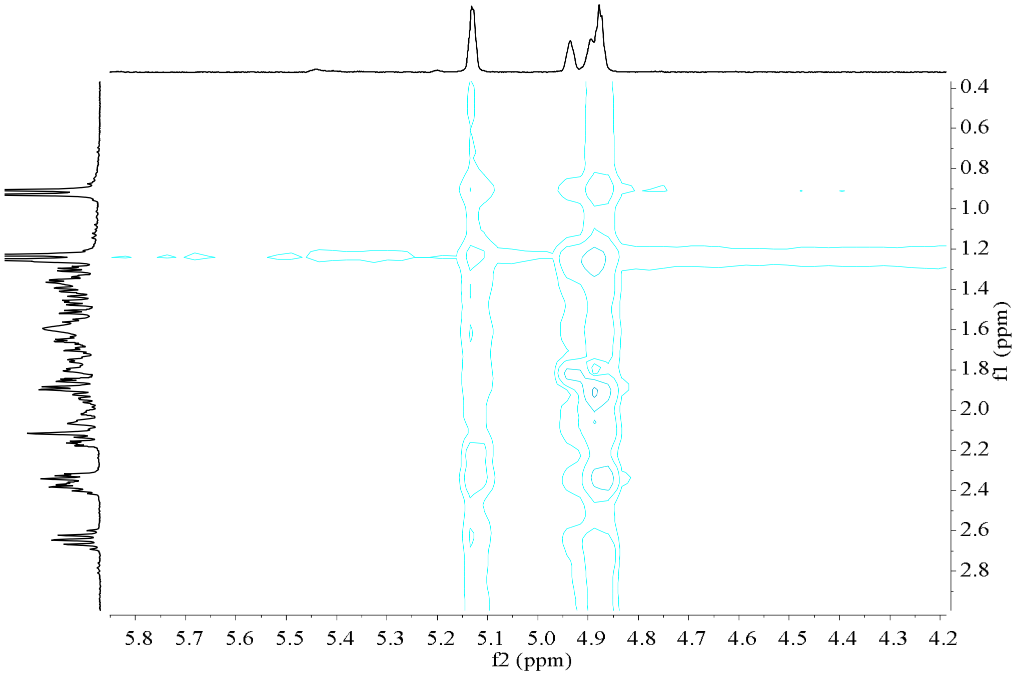

77

78 **Figure S 34. A Segment of NOESY spectrum of compound 2 in $CDCl_3$**

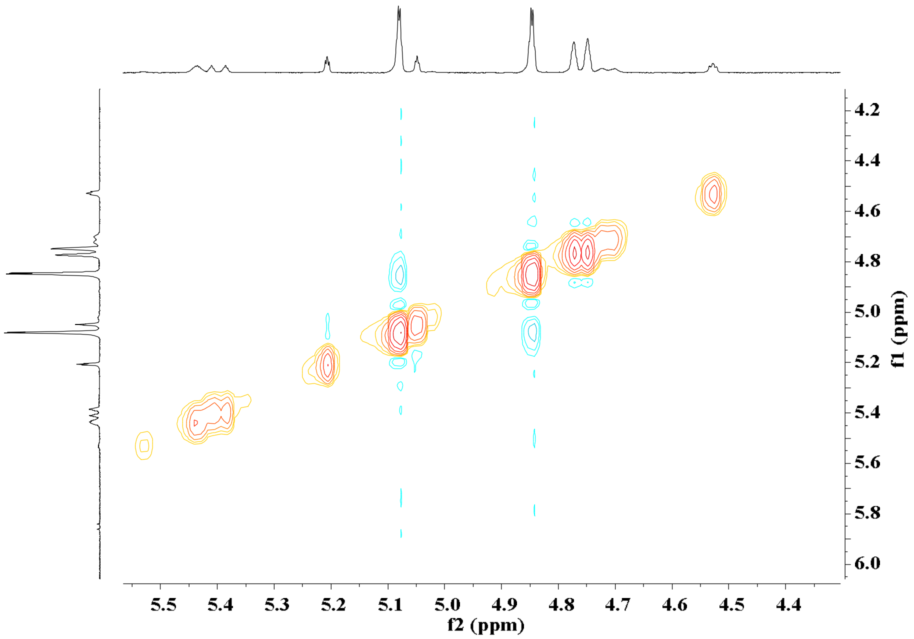

79

80 **Figure S 35. A Segment of NOESY spectrum of compound 2 in $CDCl_3$**

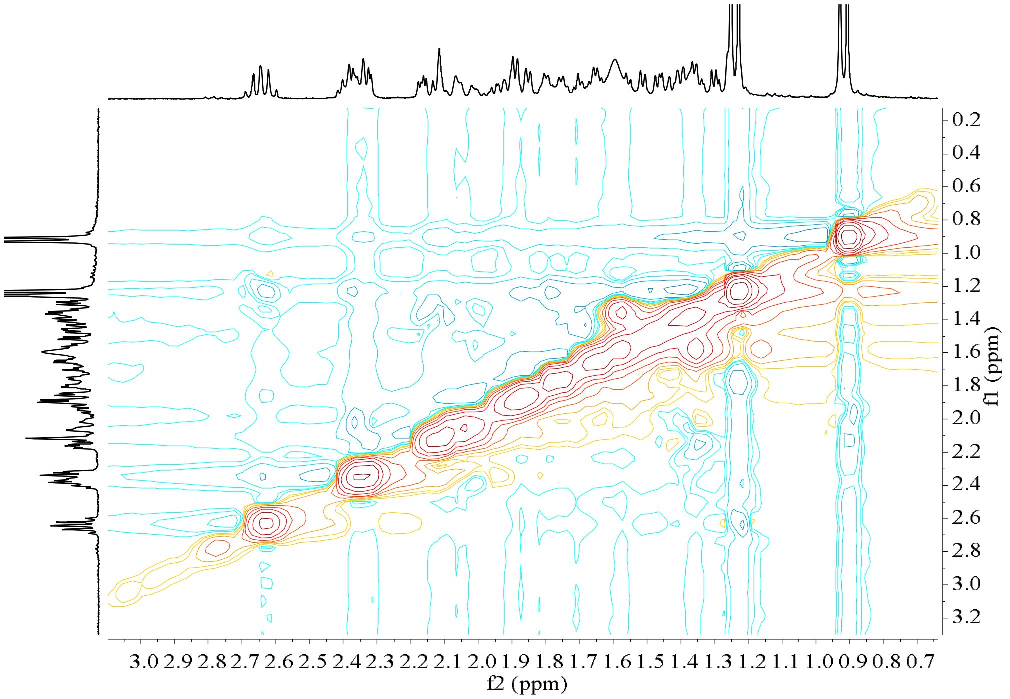

Figure S 36. A Segment of NOESY spectrum of compound 2 in $CDCl_3$

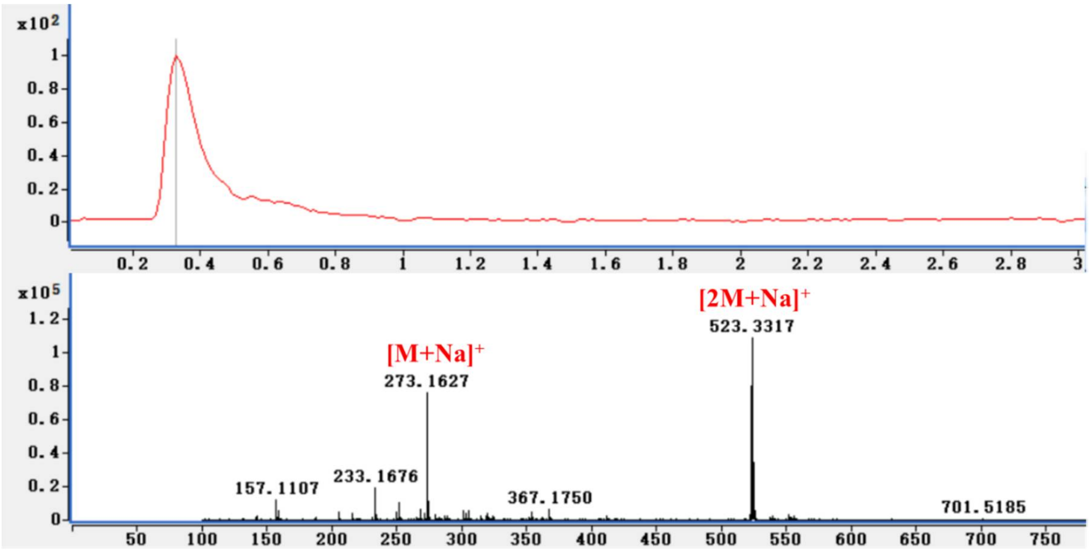

Figure S 37. HRESIMS spectrum of compound 3

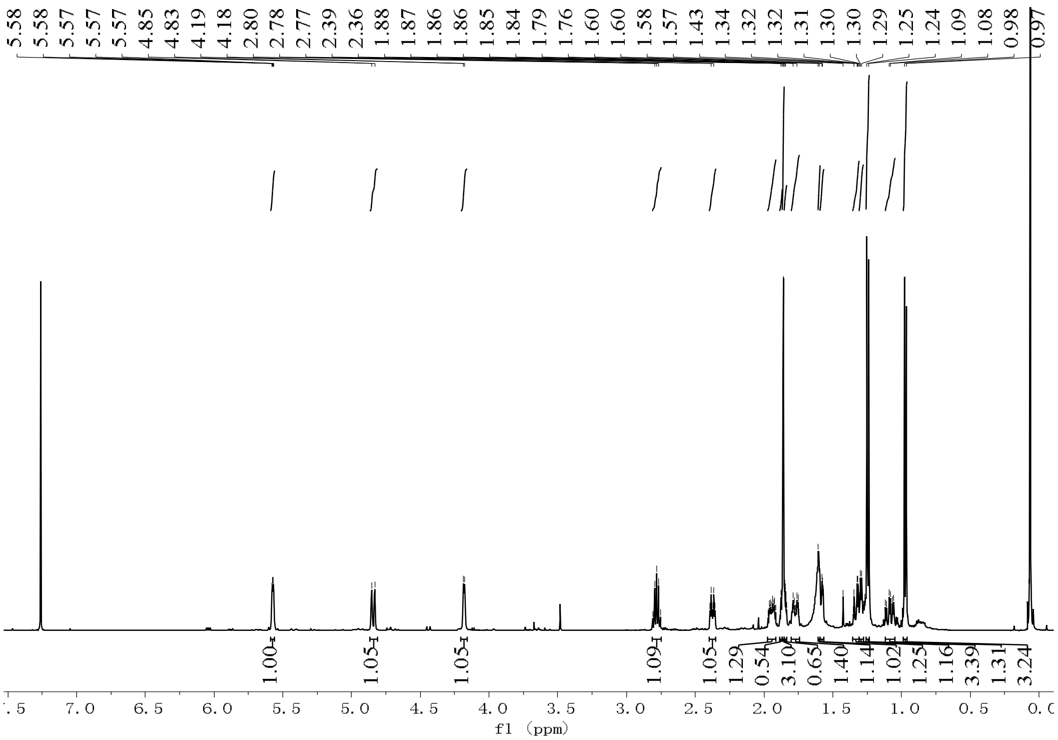

Figure S 38. ^{1}H **NMR spectrum of compound 3 in** $CDCl_3$

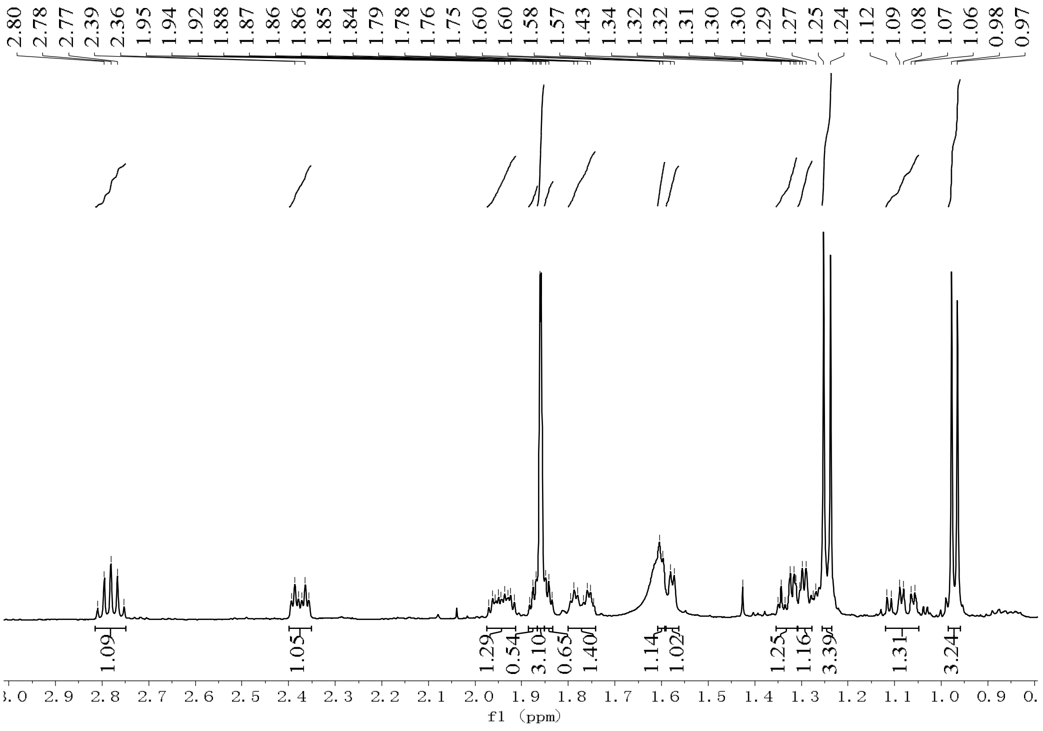

Figure S 39. Enlarged ^{1}H **NMR spectrum of compound 3 in** $CDCl_3$

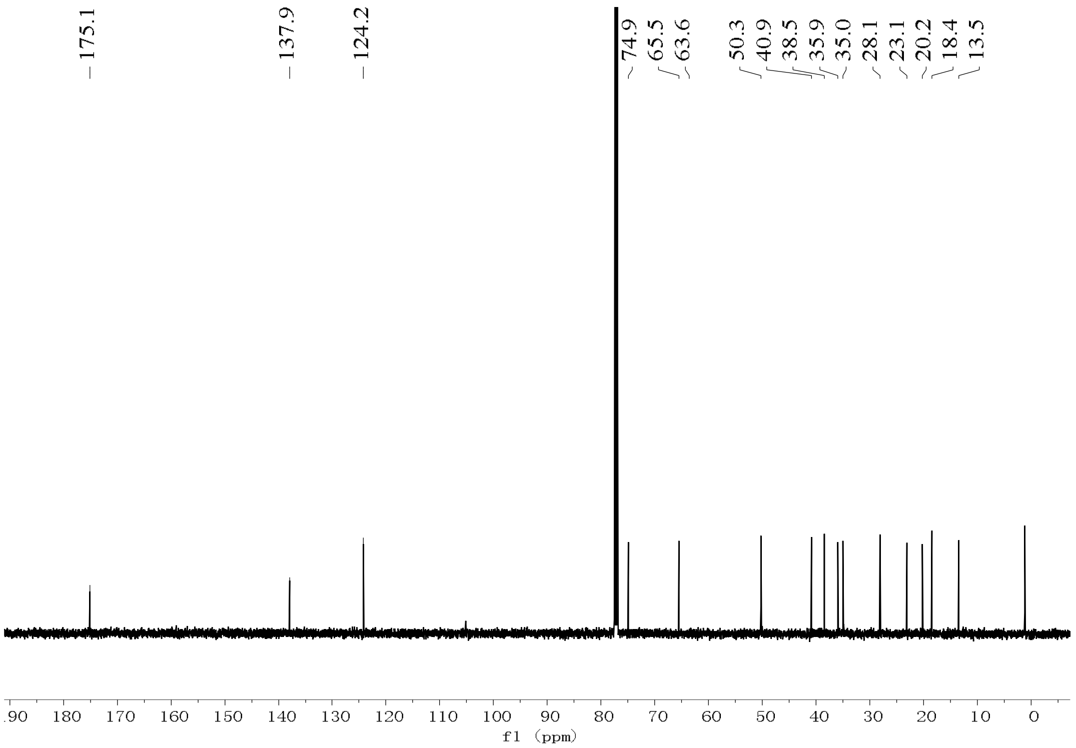

Figure S 40. ^{13}**C NMR spectrum of compound 3 in CDCl**$_3$

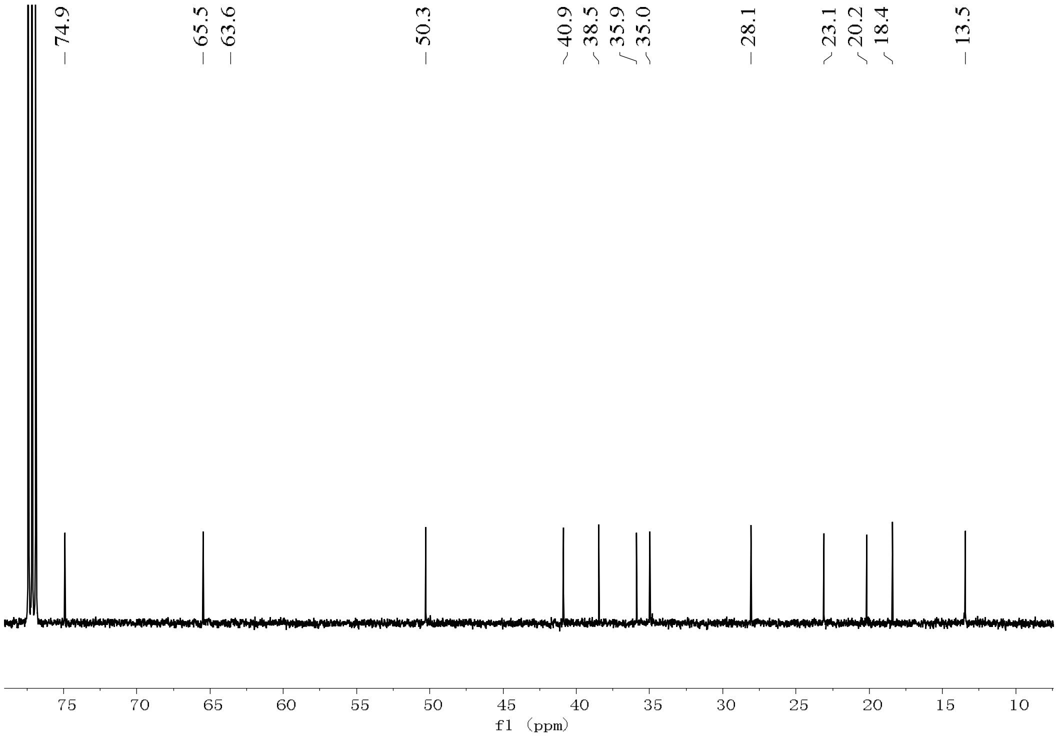

Figure S 41. A Segment of ^{13}**C NMR spectrum of compound 3 in CDCl**$_3$

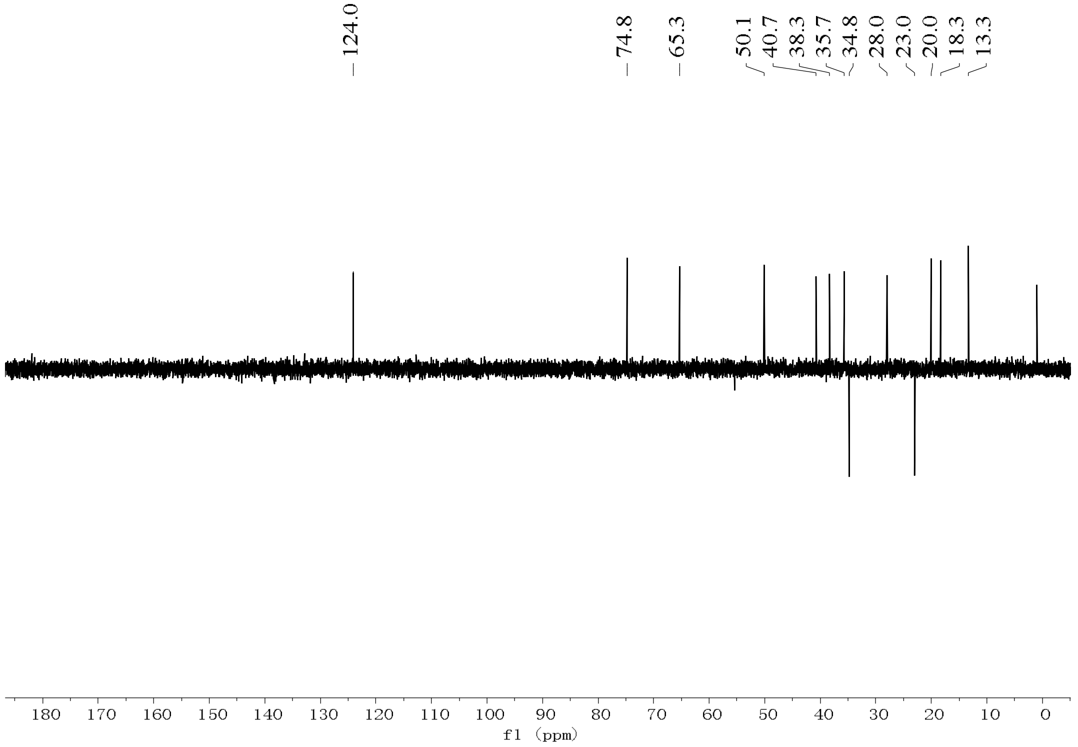

Figure S 42. DEPT135 of compound 3 in $CDCl_3$

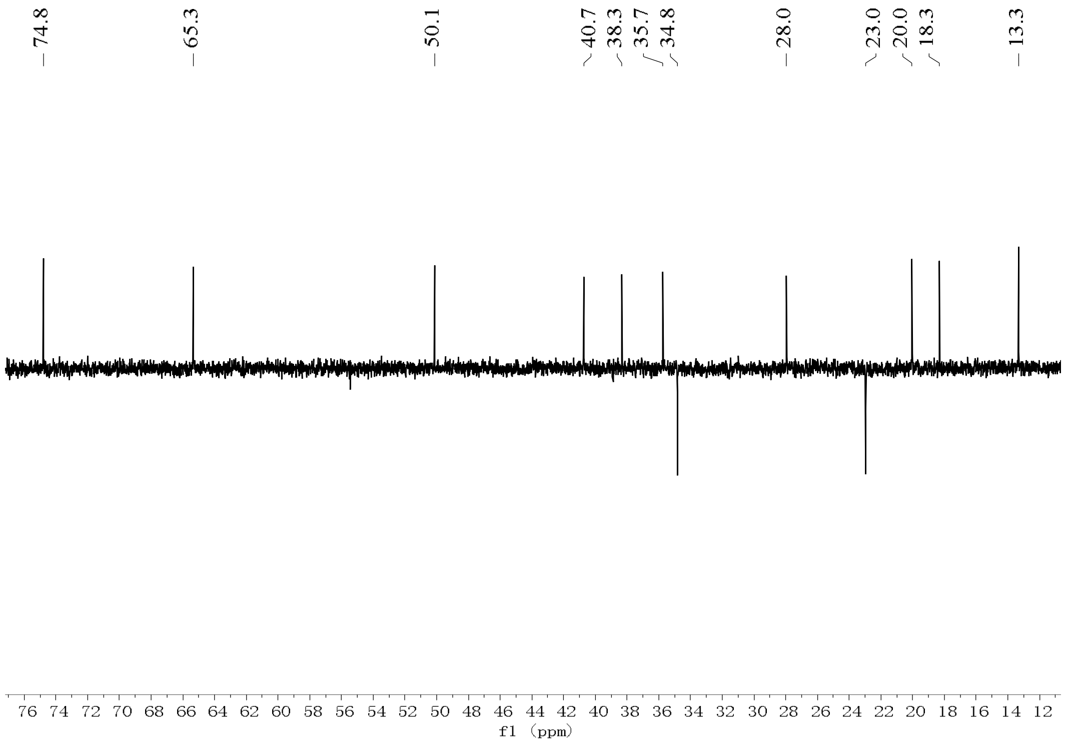

Figure S 43. A Segment of DEPT135 of compound 3 in $CDCl_3$

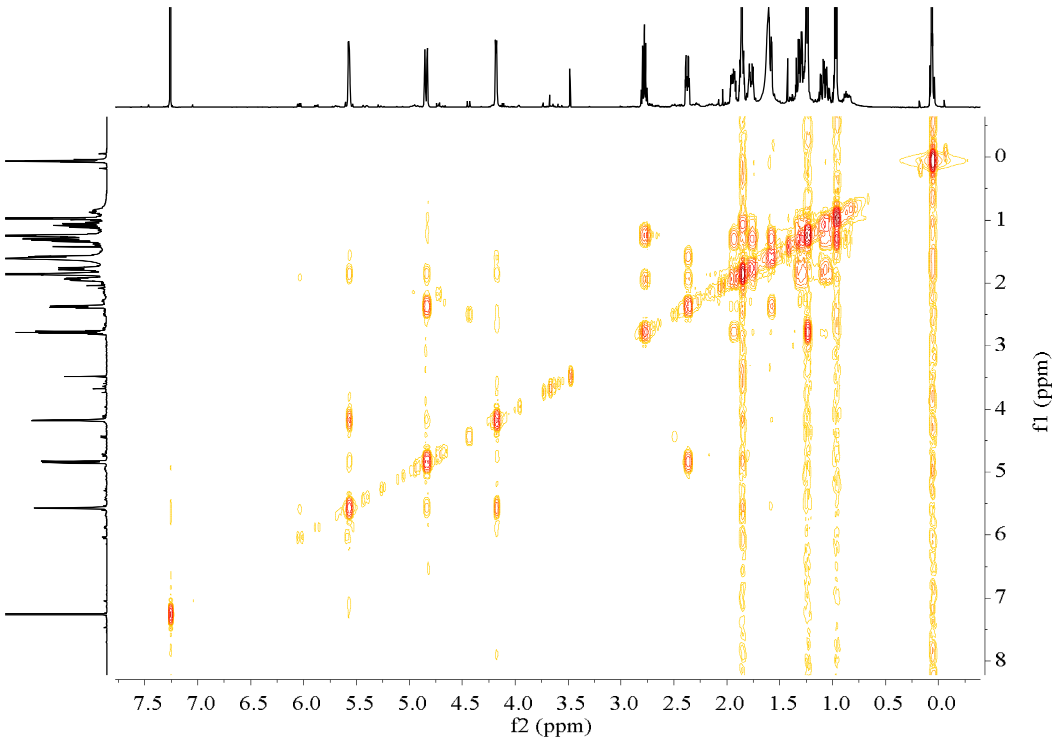

Figure S 44. ^{1}H-^{1}H COSY spectrum of compound 3 in $CDCl_3$

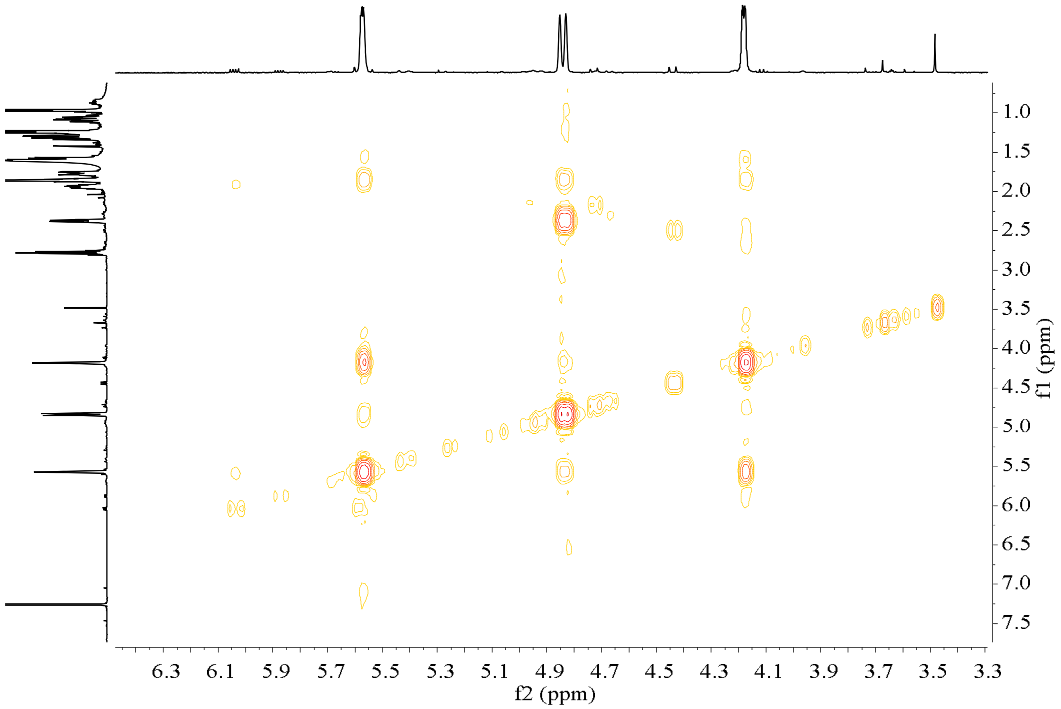

Figure S 45. A Segment of ^{1}H-^{1}H COSY spectrum of compound 3 in $CDCl_3$

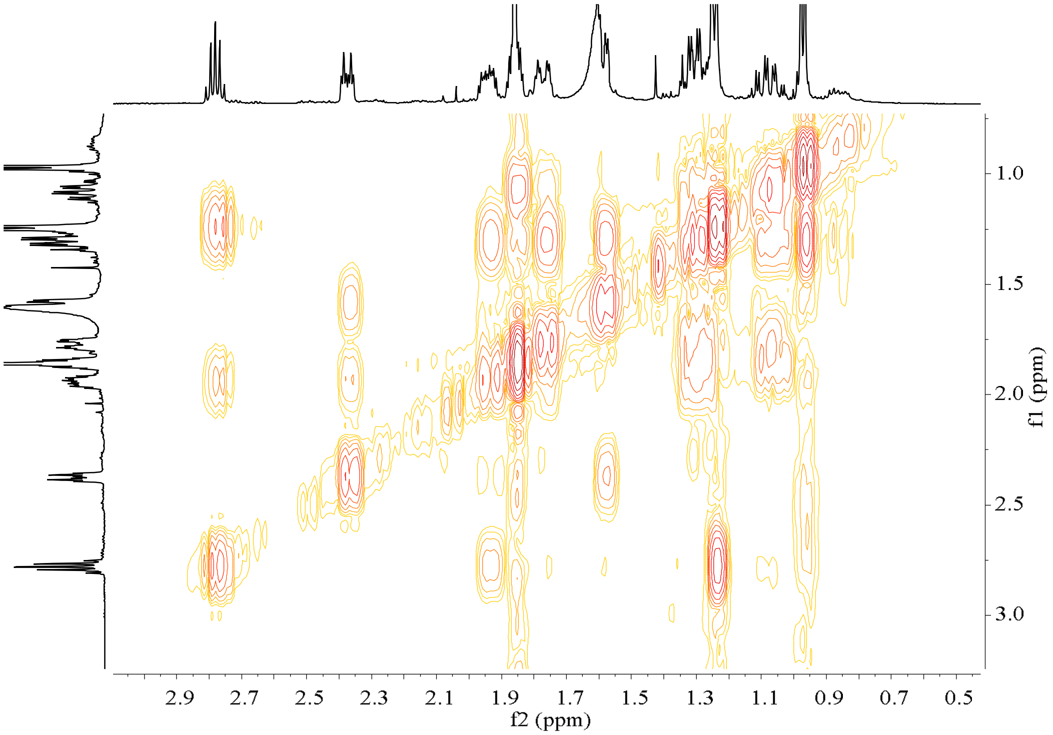

Figure S 46. A Segment of ^{1}H-^{1}H COSY spectrum of compound 3 in $CDCl_3$

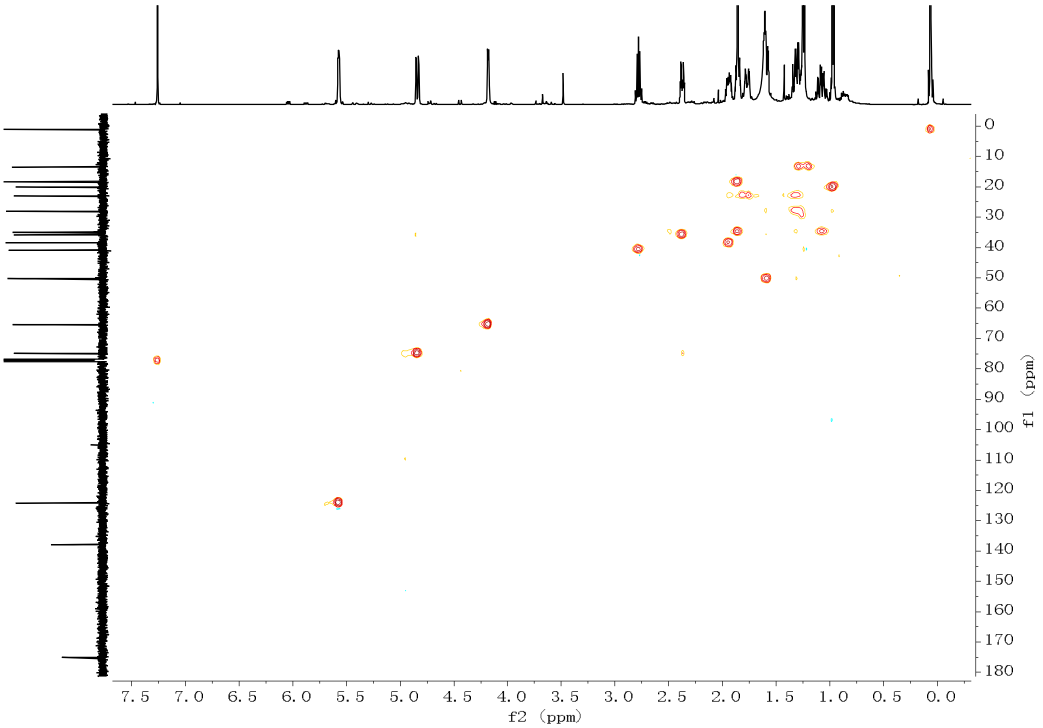

Figure S 47. HSQC spectrum of compound 3 in $CDCl_3$

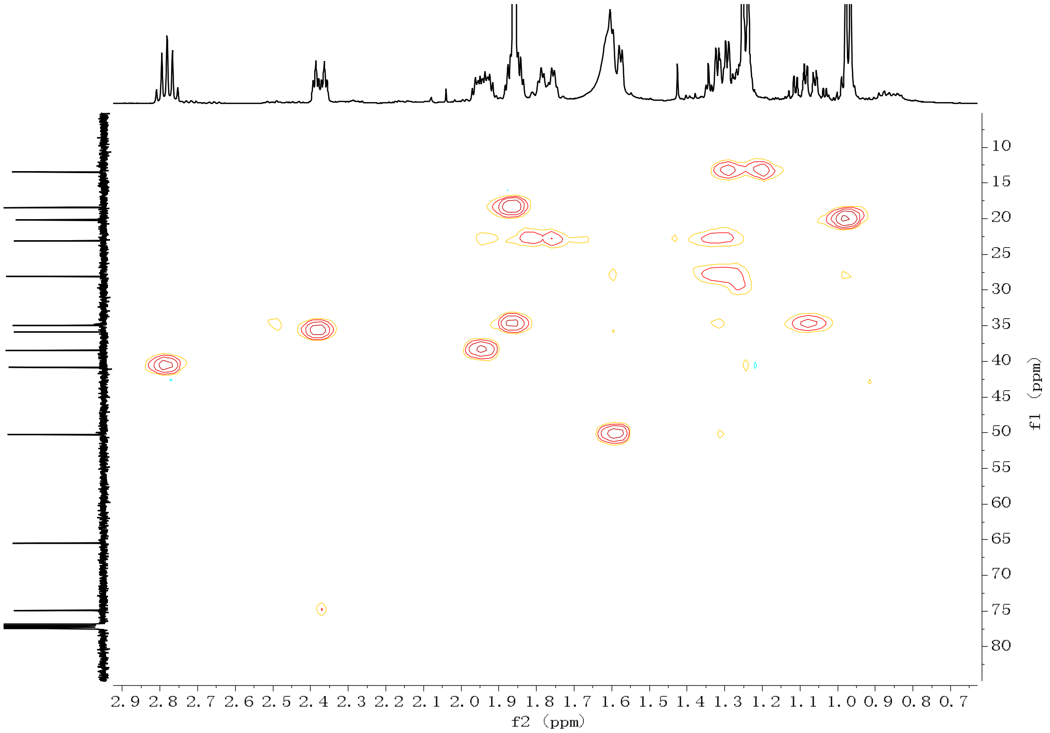

Figure S 48. A Segment of HSQC spectrum of compound 3 in $CDCl_3$

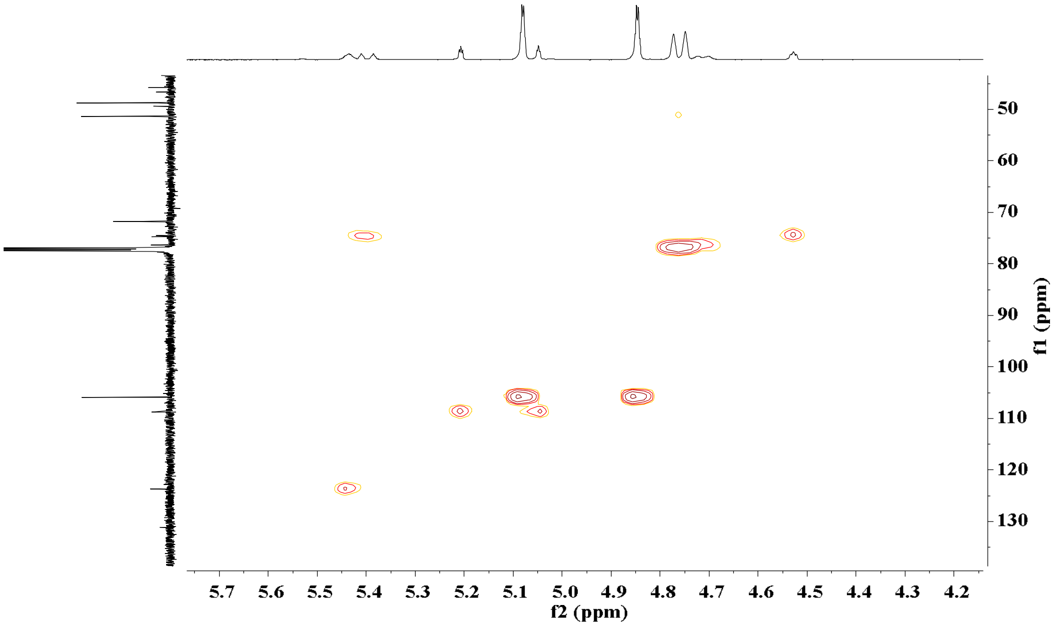

Figure S 49. A Segment of HSQC spectrum of compound 3 in $CDCl_3$

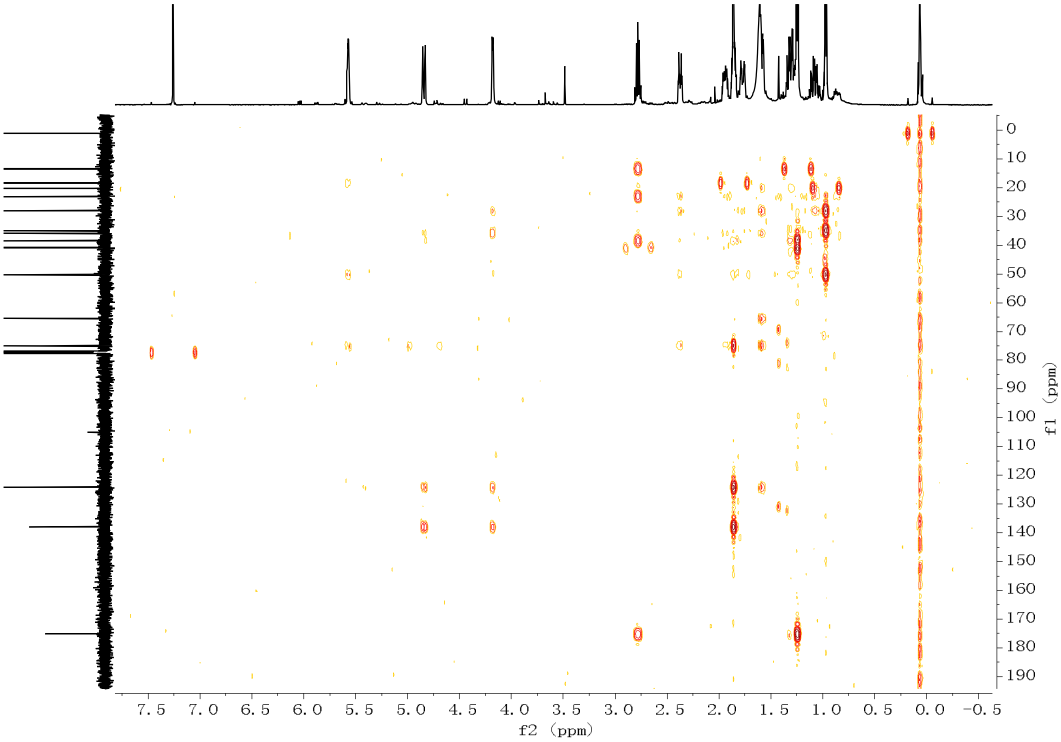

Figure S 50. HMBC spectrum of compound 3 in $CDCl_3$

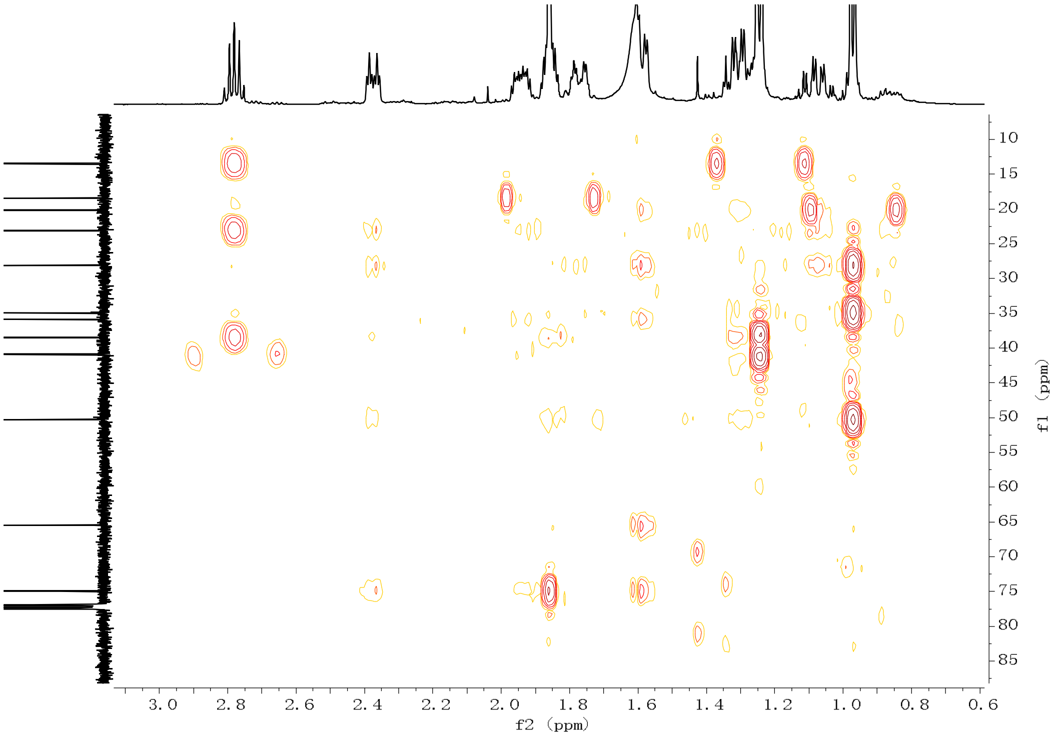

Figure S 51. A Segment of HMBC spectrum of compound 3 in $CDCl_3$

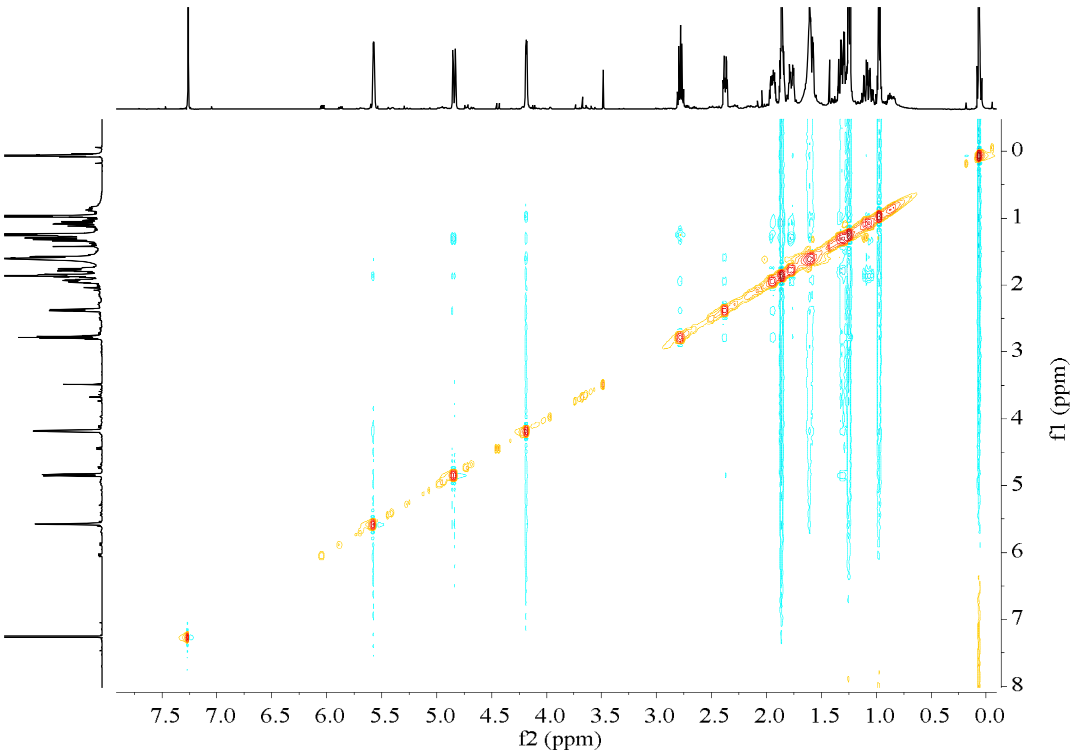

Figure S 52. NOESY spectrum of compound 3 in $CDCl_3$

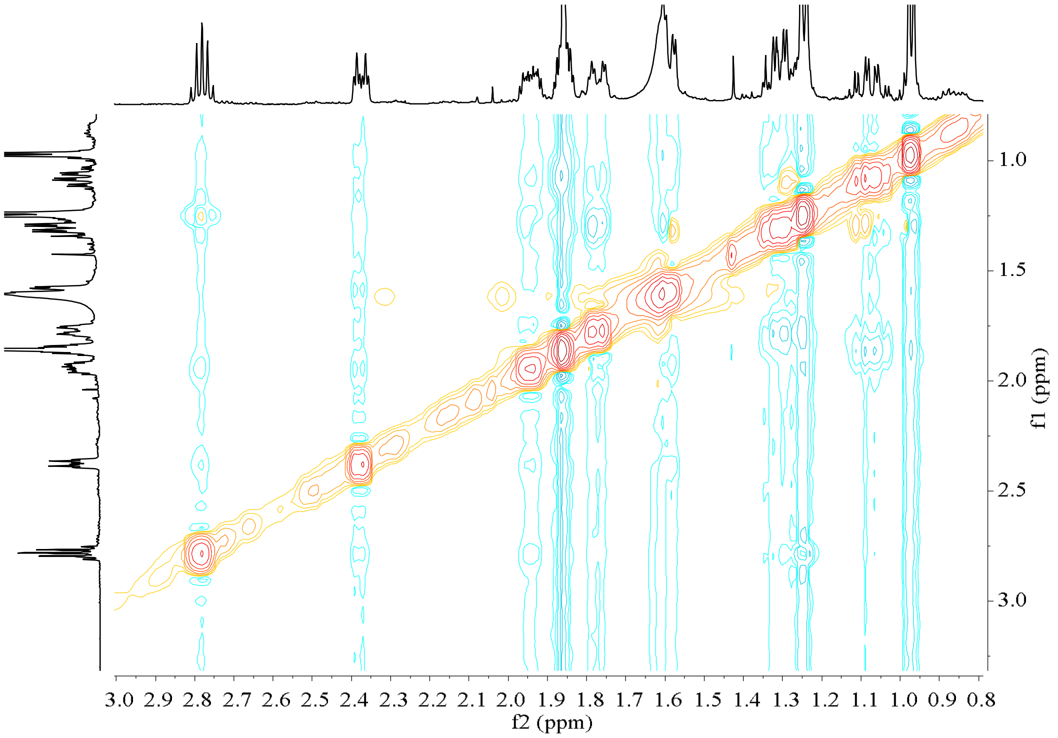

Figure S 53. A Segment of NOESY spectrum of compound 3 in $CDCl_3$

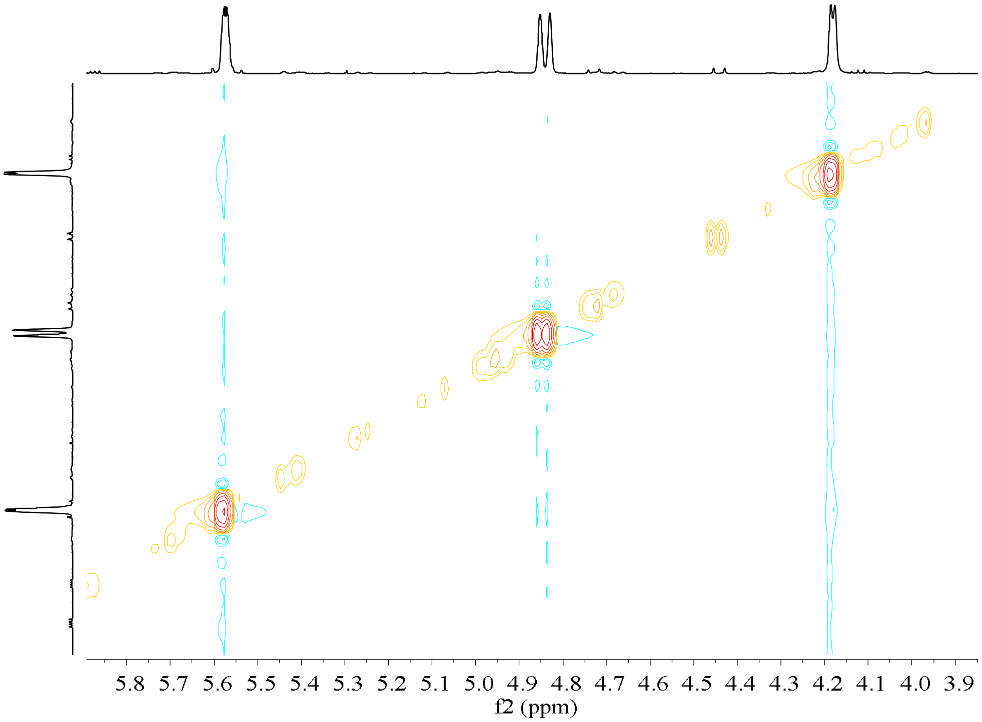

Figure S 54. A Segment of NOESY spectrum of compound 3 in $CDCl_3$

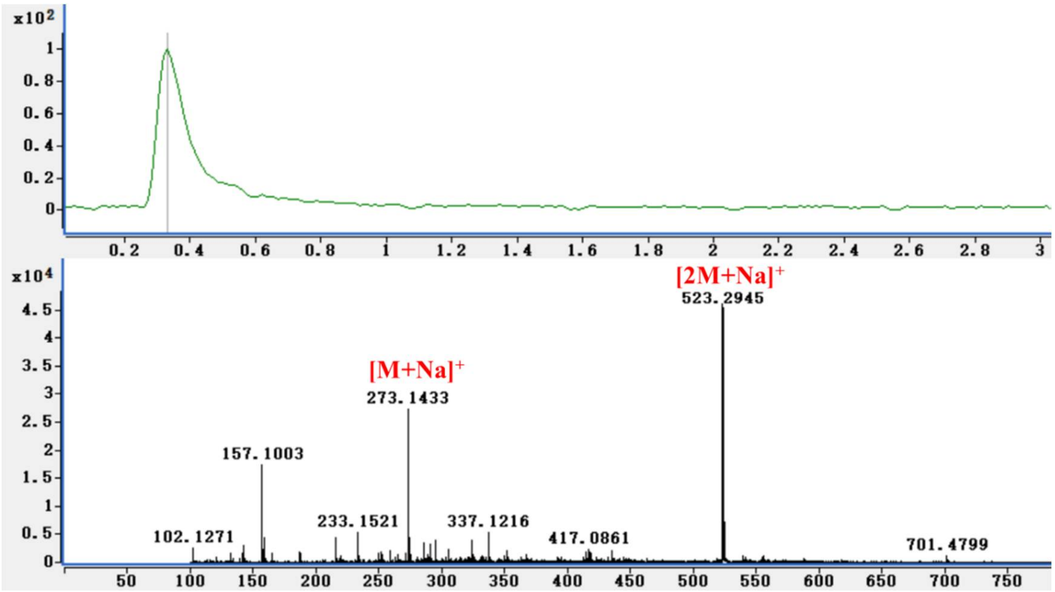

Figure S 55. HRESIMS spectrum of compound 4

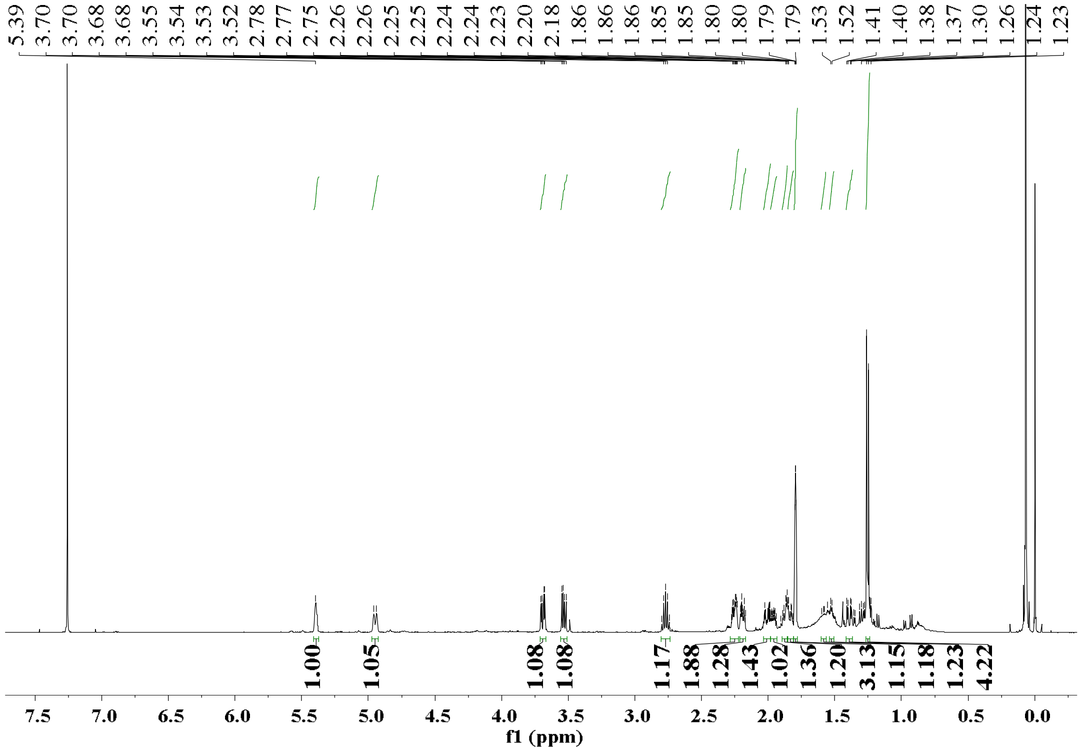

Figure S 56. ^{1}**H NMR spectrum of compound 4 in** $CDCl_3$

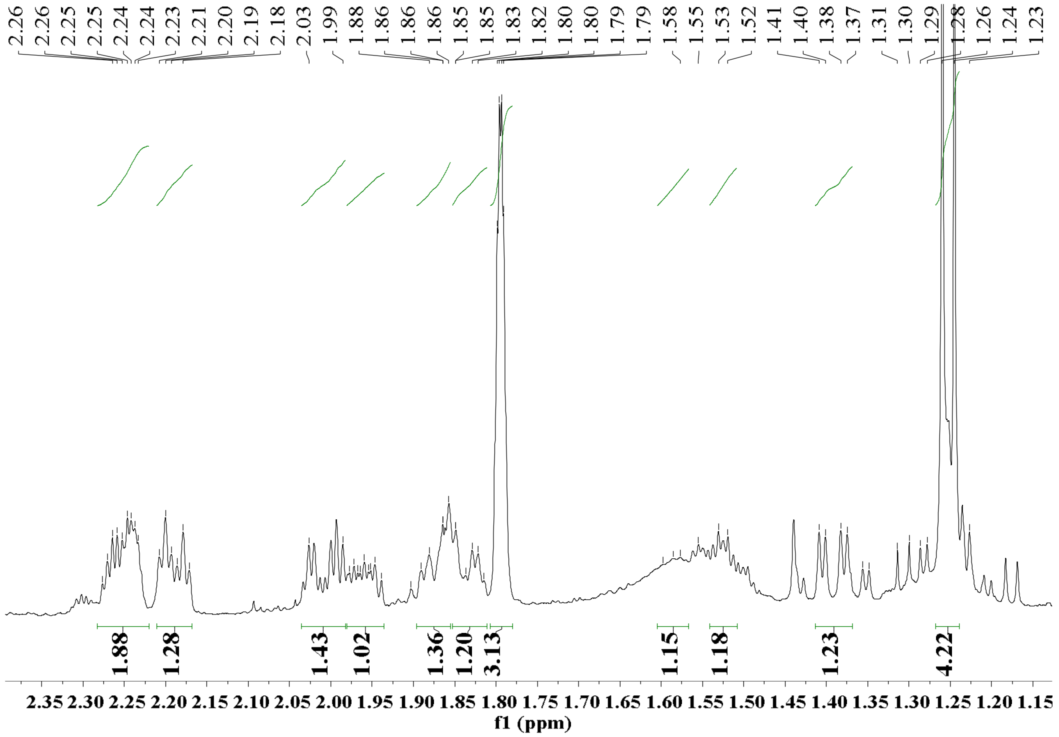

Figure S 57. A Segment of ^{1}**H NMR spectrum of compound 4 in** $CDCl_3$

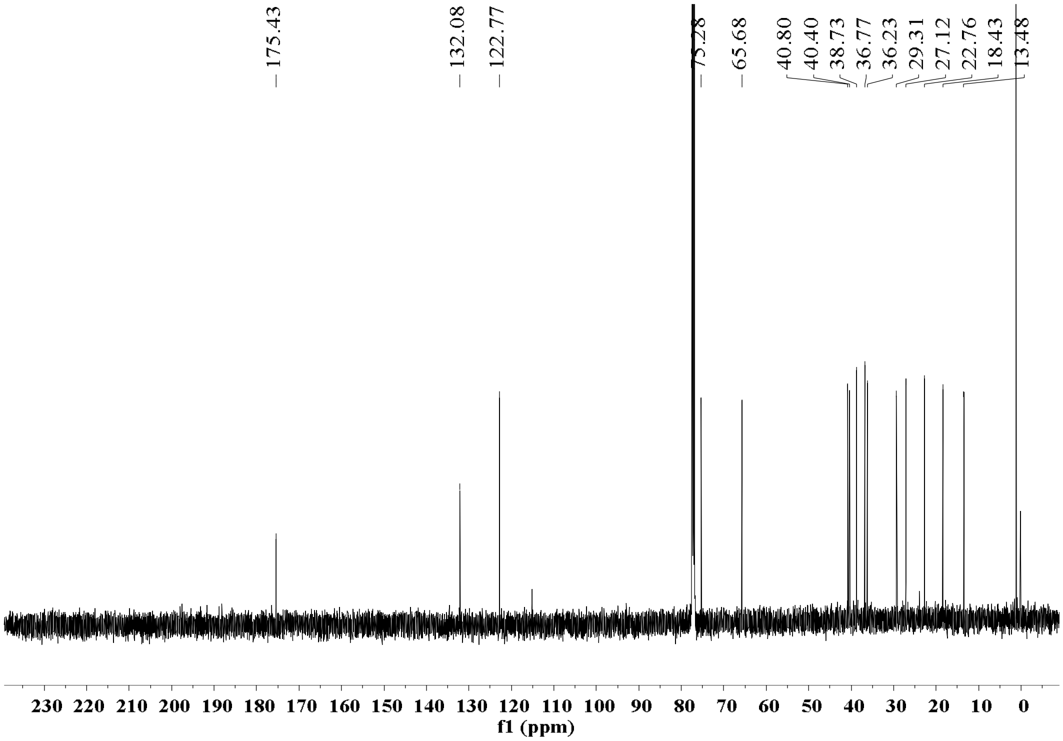

Figure S 58. ^{13}C NMR spectrum of compound 4 in $CDCl_3$

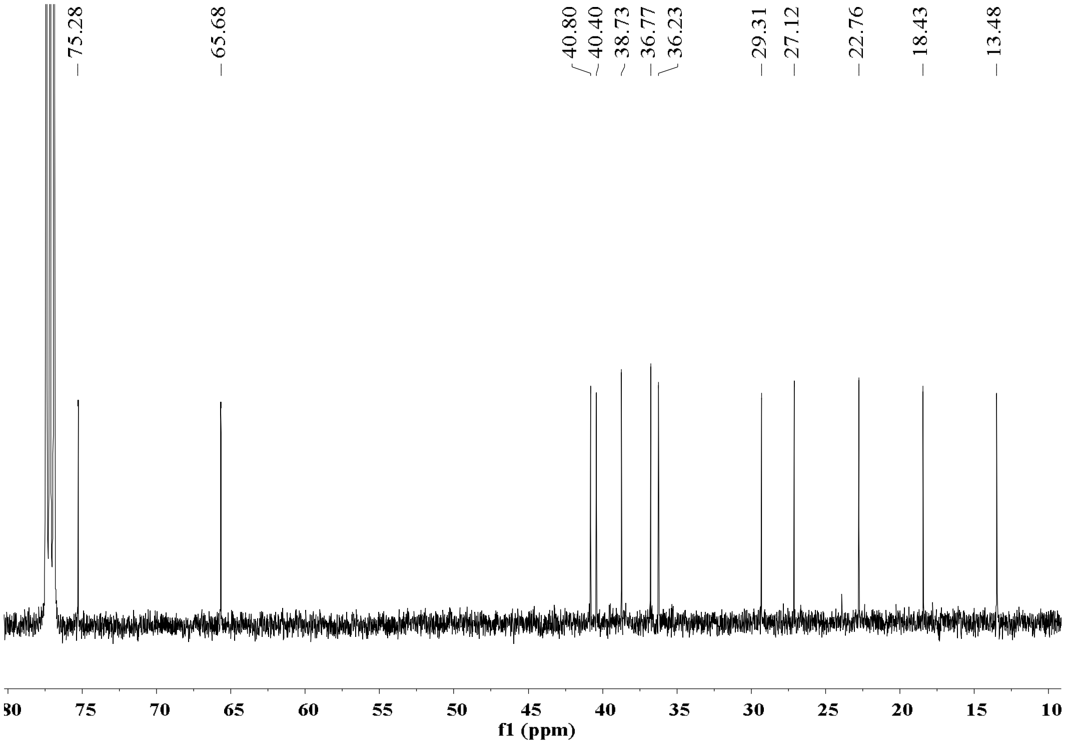

Figure S 59. A Segment of ^{13}C NMR spectrum of compound 4 in $CDCl_3$

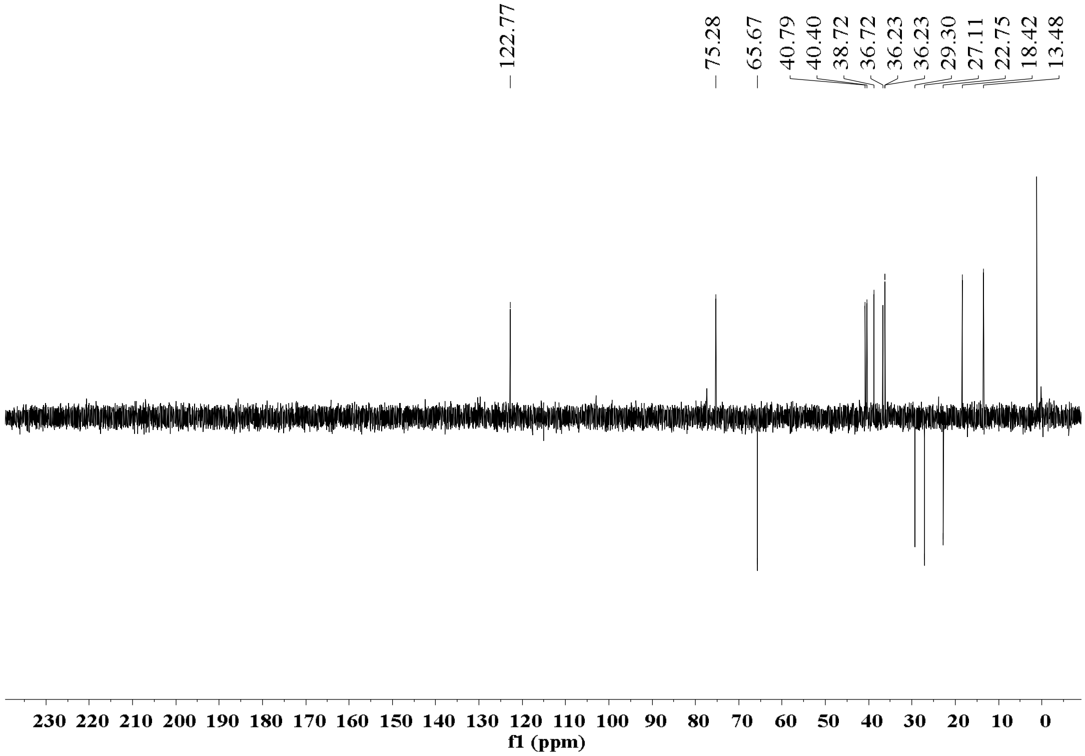

Figure S 60. DEPT135 of compound 4 in $CDCl_3$

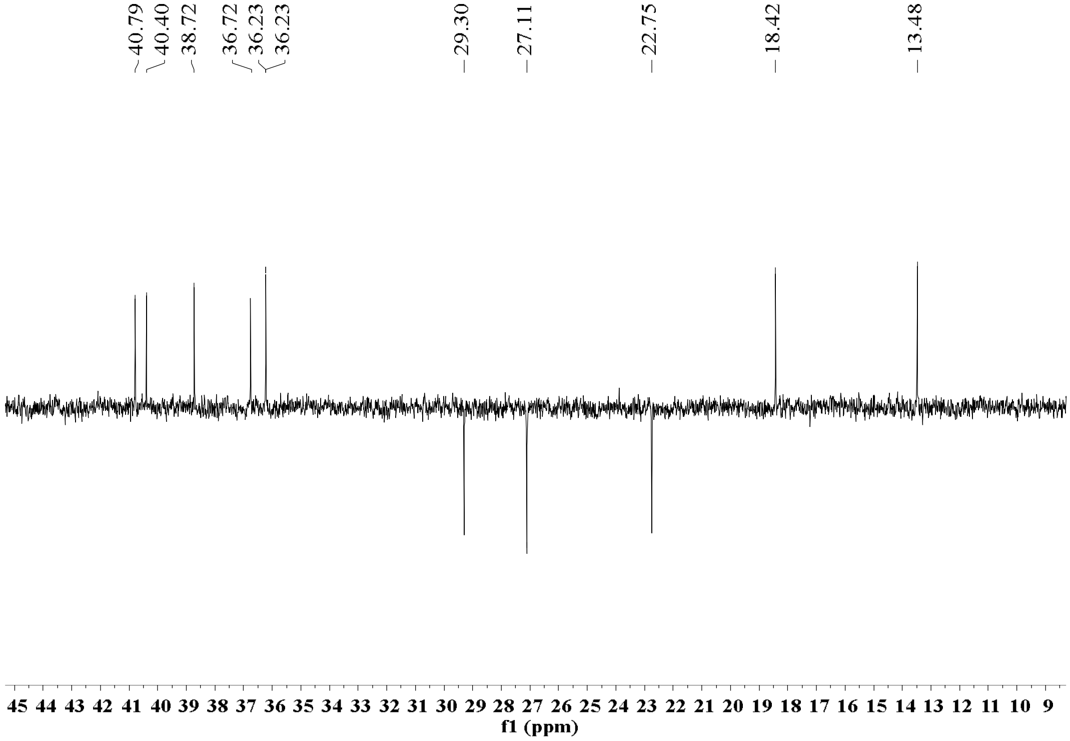

Figure S 61. A Segment of DEPT135 of compound 4 in $CDCl_3$

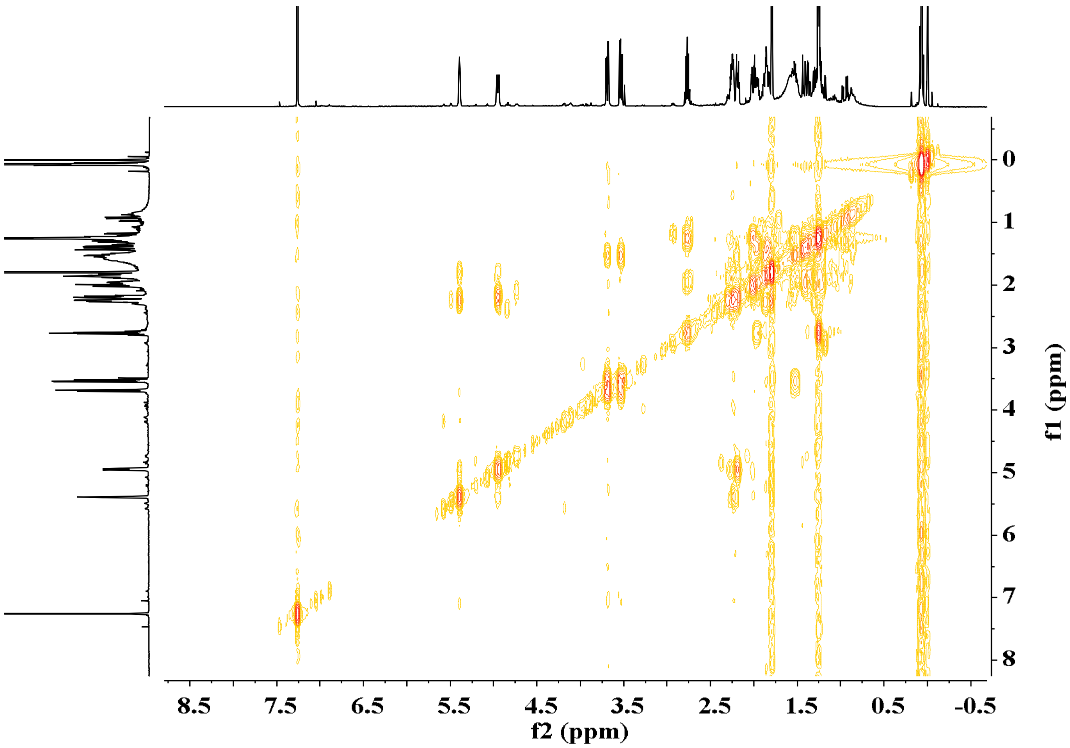

Figure S 62. ^{1}H-^{1}H COSY spectrum of compound 4 in $CDCl_3$

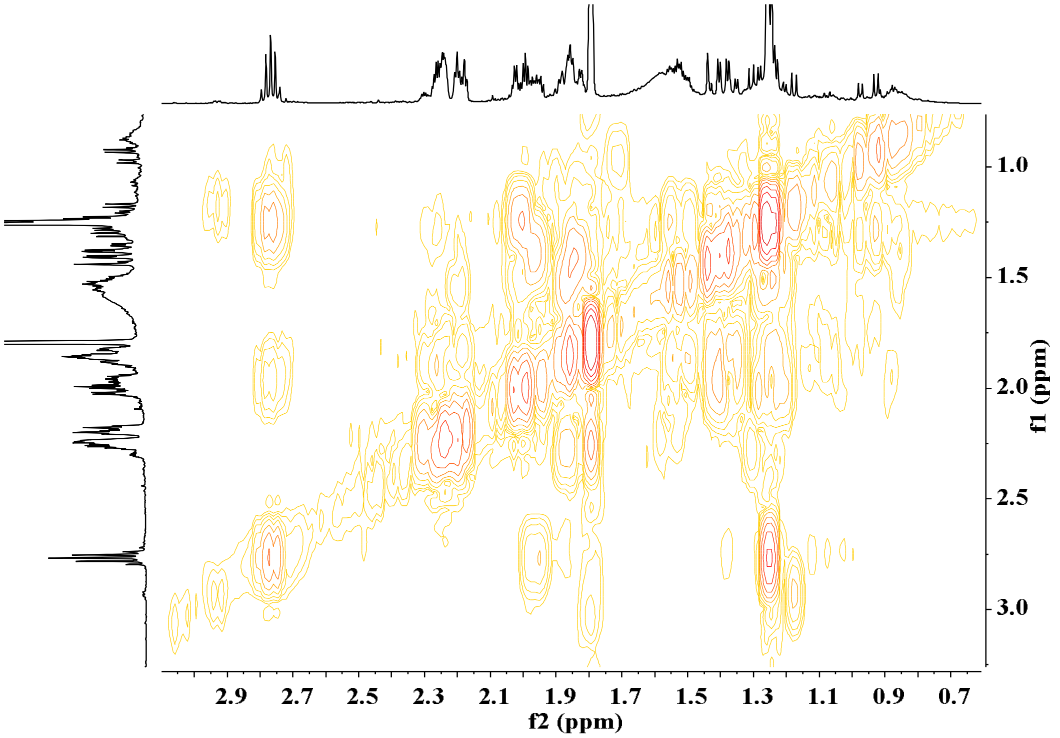

Figure S 63. A Segment of ^{1}H-^{1}H COSY spectrum of compound 4 in $CDCl_3$

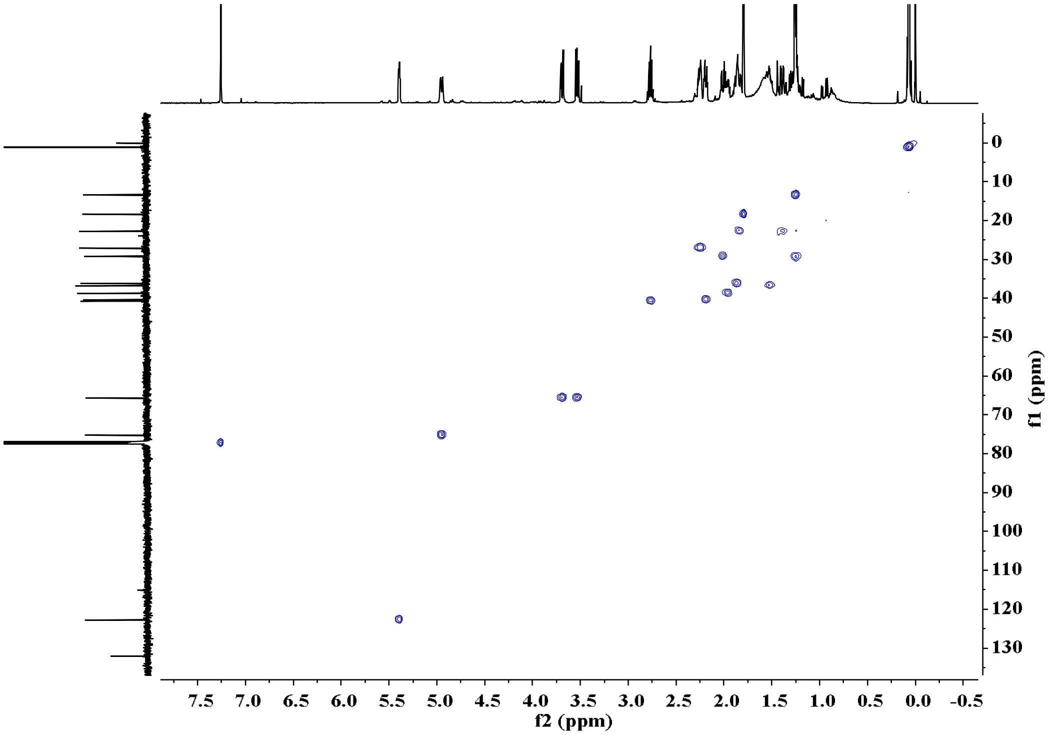

Figure S 64. HSQC spectrum of compound 4 in $CDCl_3$

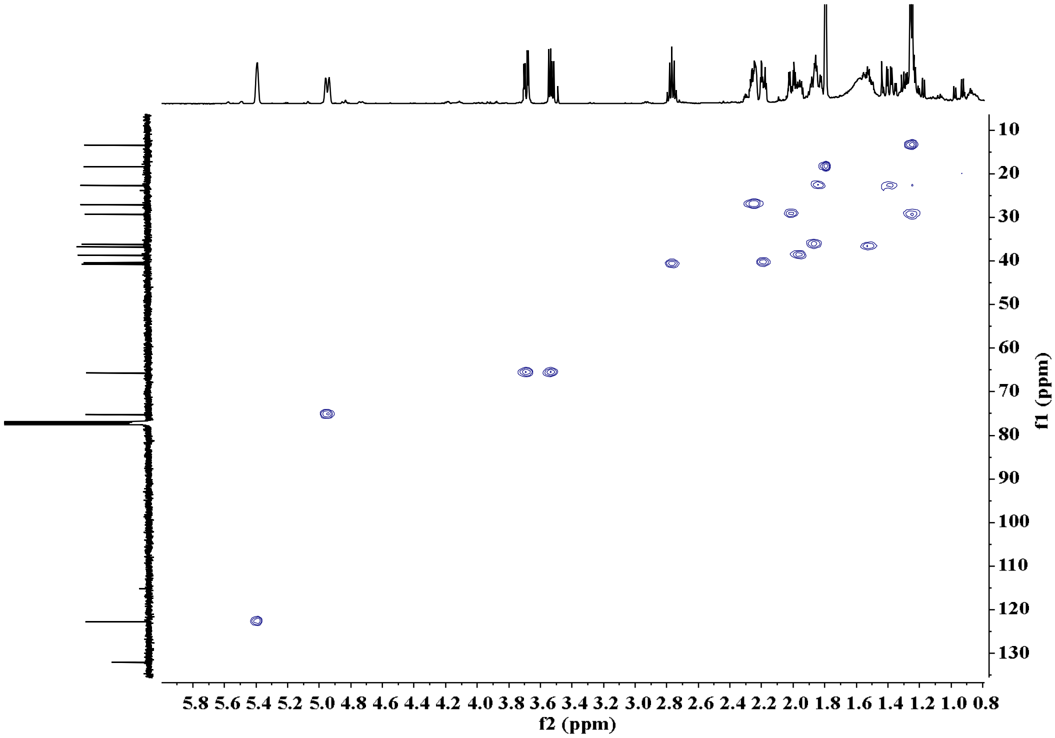

Figure S 65. A Segment of HSQC spectrum of compound 4 in $CDCl_3$

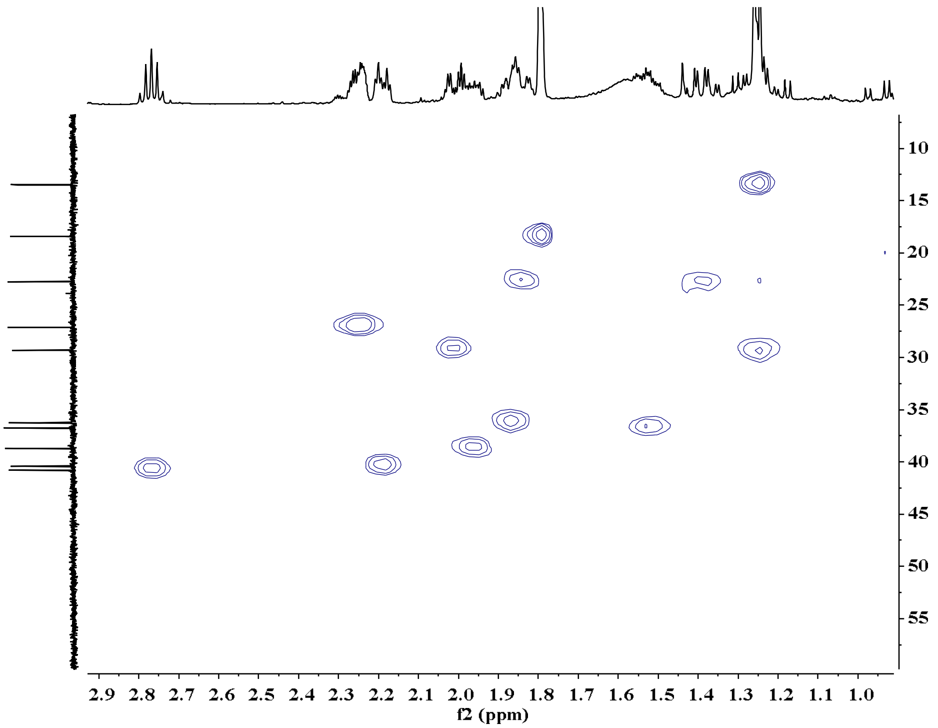

Figure S 66. A Segment of HSQC spectrum of compound 4 in $CDCl_3$

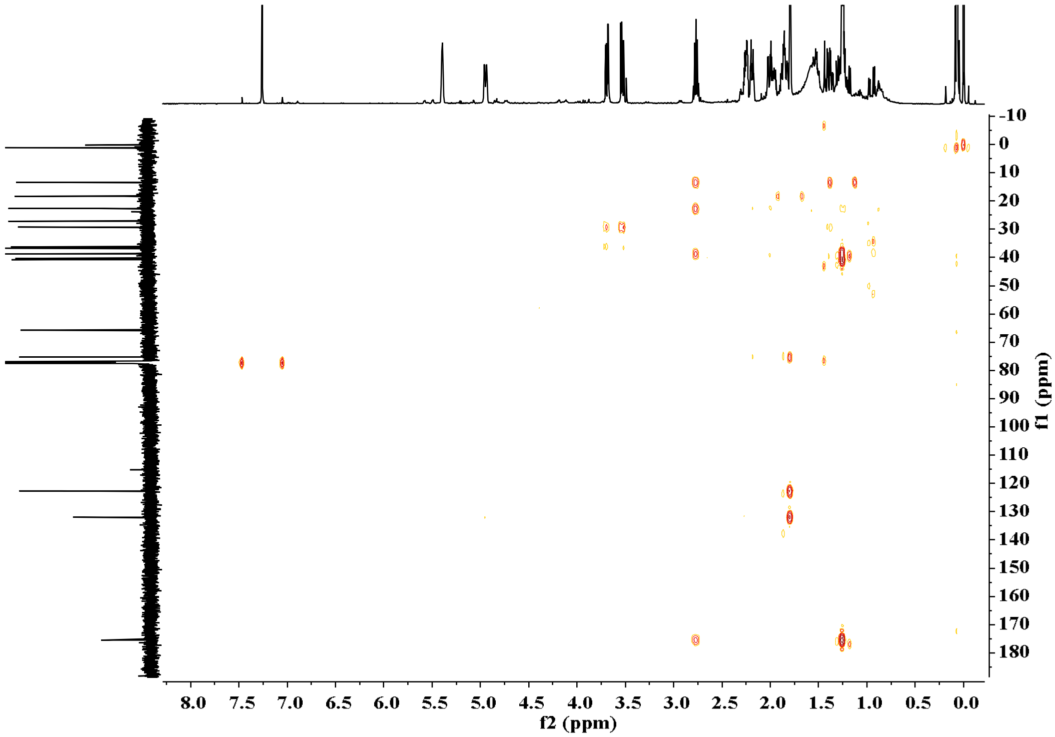

Figure S 67. HMBC spectrum of compound 4 in $CDCl_3$

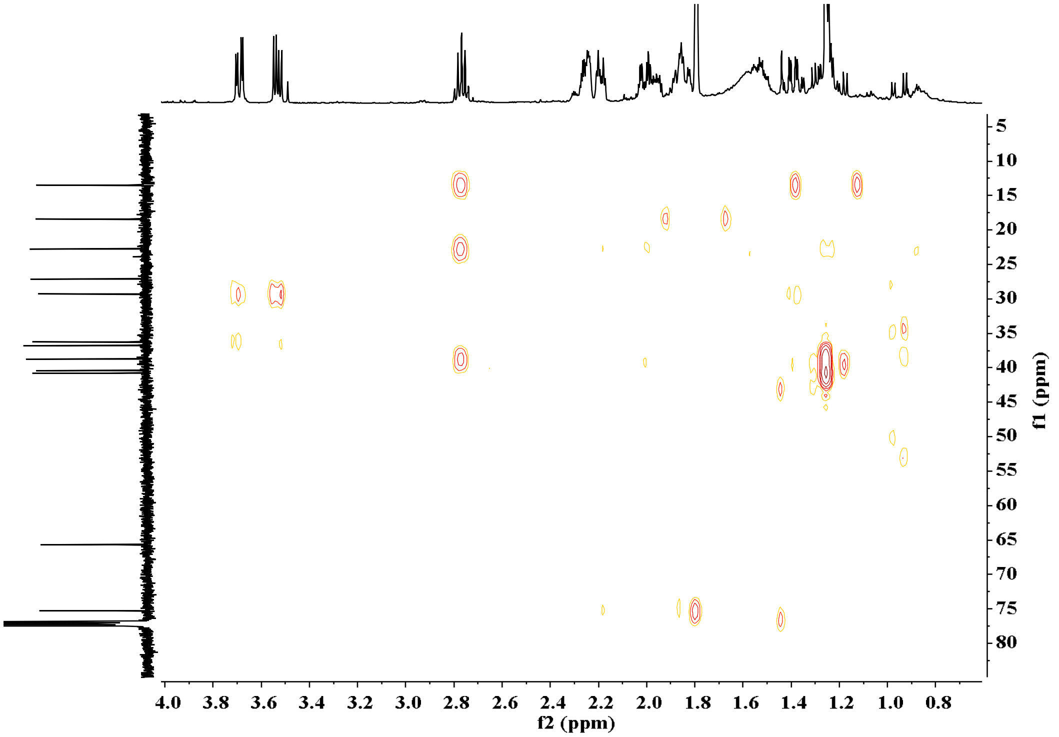

Figure S 68. A Segment of HMBC spectrum of compound 4 in $CDCl_3$

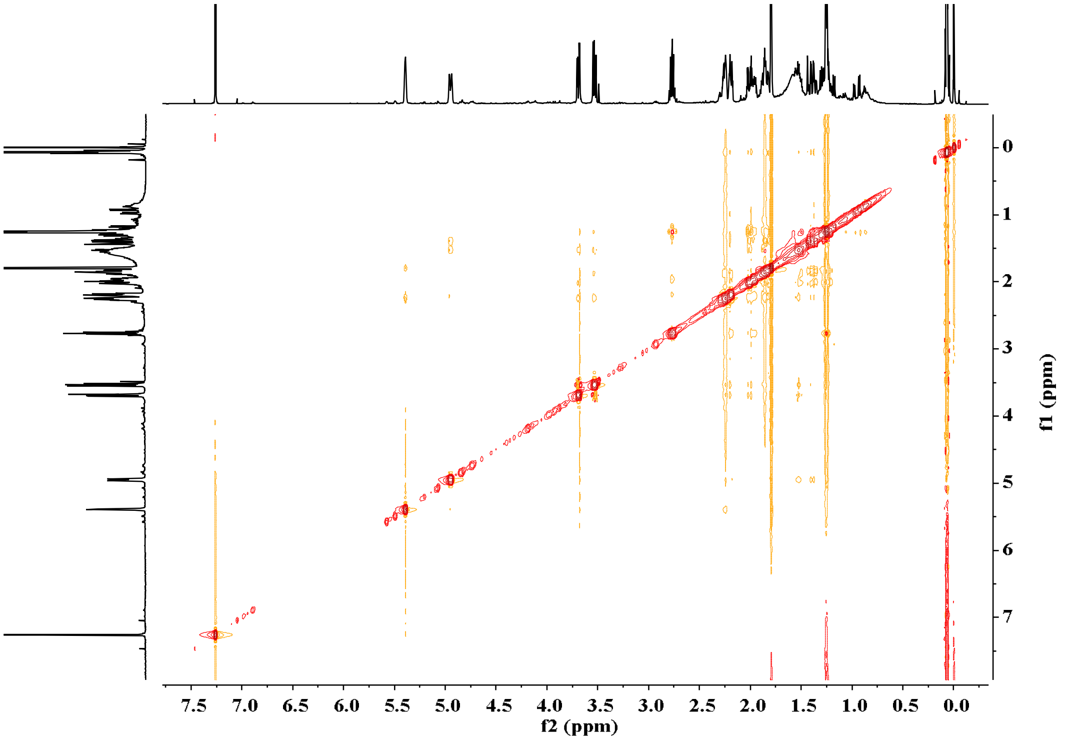

Figure S 69. NOESY spectrum of compound 4 in $CDCl_3$

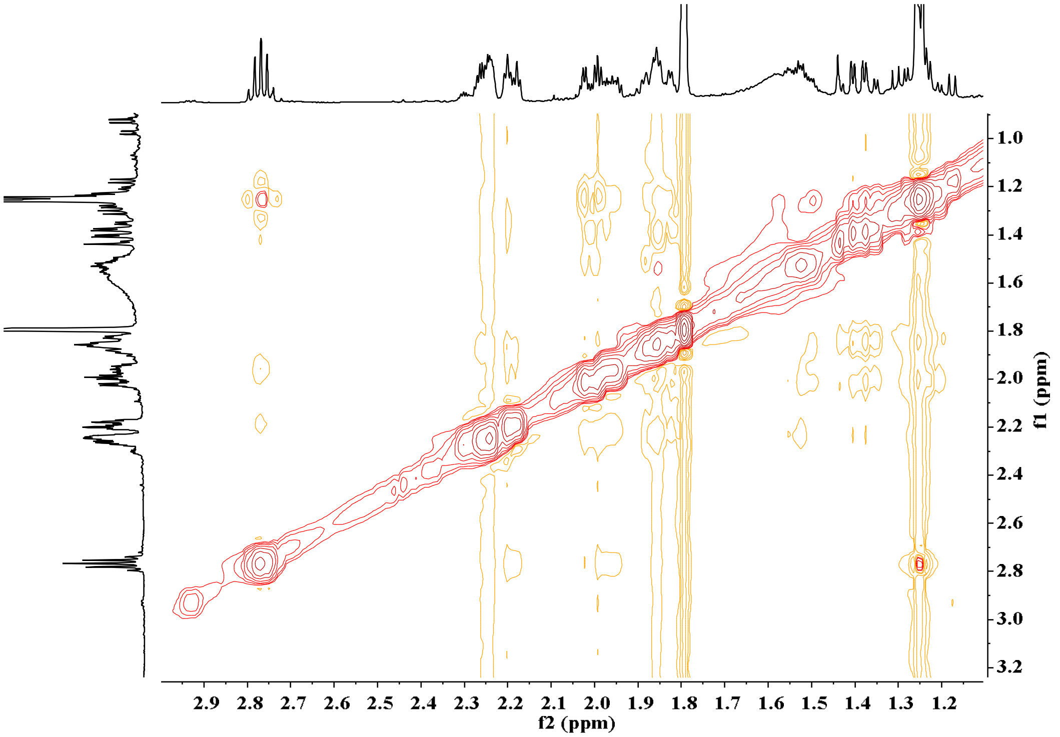

Figure S 70. A Segment of NOESY spectrum of compound 4 in $CDCl_3$

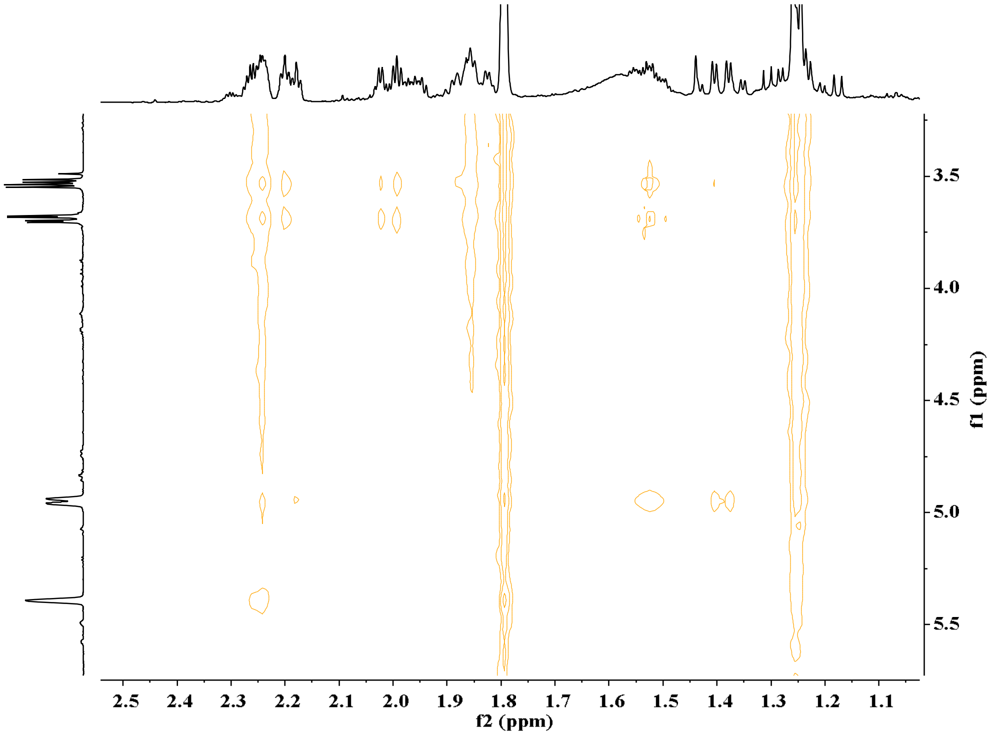

Figure S 71. A Segment of NOESY spectrum of compound 4 in $CDCl_3$

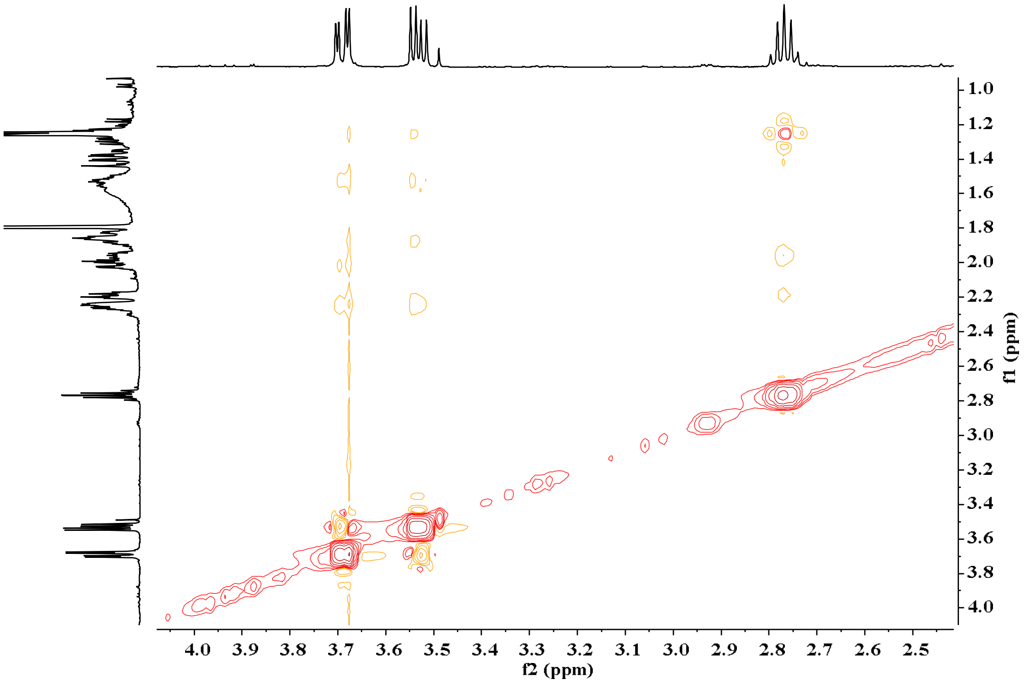

Figure S 72. A Segment of NOESY spectrum of compound 4 in $CDCl_3$
